# 3-nitroimidazo[1,2-*b*]pyridazine as a novel scaffold for antiparasitics with sub-nanomolar anti-*Giardia lamblia* activity

**DOI:** 10.1016/j.ijpddr.2022.05.004

**Published:** 2022-05-26

**Authors:** Yang Zheng, Joachim Müller, Stefan Kunz, Marco Siderius, Louis Maes, Guy Caljon, Norbert Müller, Andrew Hemphill, Geert Jan Sterk, Rob Leurs

**Affiliations:** a Amsterdam Institute for Molecules, Medicines and Systems, Division of Medicinal Chemistry, Faculty of Science, Vrije Universiteit Amsterdam, De Boelelaan 1108, 1081 HZ, Amsterdam, the Netherlands; b Vetsuisse Faculty, Institute of Parasitology, University of Bern, Länggass-Strasse 122, CH-3012, Bern, Switzerland; c Laboratory of Microbiology, Parasitology and Hygiene (LMPH), University of Antwerp, Universiteitsplein 1, Wilrijk, 2610, Belgium

**Keywords:** 3-nitroimidazo[1,2-*b*]pyridazine, Synthesis, *Giardia lamblia*, 3′,5′-cyclic nucleotide phosphodiesterase, *In vitro*

## Abstract

As there is a continuous need for novel anti-infectives, the present study aimed to fuse two modes of action into a novel 3-nitroimidazo[1,2-*b*]pyridazine scaffold to improve antiparasitic efficacy. For this purpose, we combined known structural elements of phosphodiesterase inhibitors, a target recently proposed for *Trypanosoma brucei* and *Giardia lamblia*, with a nitroimidazole scaffold to generate nitrosative stress. The compounds were evaluated *in vitro* against a panel of protozoal parasites, namely *Giardia lamblia*, *Trypanosoma brucei*, *T. cruzi*, *Leishmania infantum* and *Plasmodium falciparum* and for cytotoxicity on MRC-5 cells. Interestingly, selective sub-nanomolar activity was obtained against *G. lamblia*, and by testing several analogues with and without the nitro group, it was shown that the presence of a nitro group, but not PDE inhibition, is responsible for the low IC_50_ values of these novel compounds. Adding the favourable drug-like properties (low molecular weight, cLogP (1.2–4.1) and low polar surface area), the key compounds from the 3-nitroimidazo[1,2-*b*]pyridazine series can be considered as valuable hits for further anti-giardiasis drug exploration and development.

## Highlights

• Analogues fusing a nitroimidazole moiety and a PDE inhibitor scaffold were prepared.
• These compounds were tested *in vitro* against a panel of protozoal parasites.
• Against *Giardia lamblia*, sub-nanomolar IC_50_ values were determined.
• PDE inhibition provided no significant contribution to the anti-*Giardia* potency.
• High potency with drug-like properties warrants further study of this hit series.

## Graphical abstract

**Figure:**
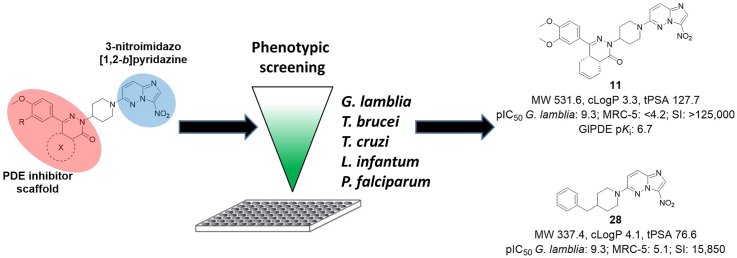

## 1 Introduction

Antibiotic, antiviral, antifungal and antiprotozoal resistance is on the rise worldwide and poses a formidable challenge in achieving a Universal Health Coverage, as highlighted in a recent report from the Interagency Coordination Group on Antimicrobial resistance to the UN Secretary-General. In a worst-case scenario, this is projected to lead to 10 million deaths annually by 2050 (World Health Organisation, 2019). As such, there is a continuous need for novel antimicrobials, which are not only valid for bacterial infections but also for parasitic diseases. An estimated disease burden for sixteen of the most common parasitic infections, most of them on the WHO list of neglected tropical diseases, came to about 3 billion cases with 1 out of every 6 persons worldwide experiencing 1 or more infections each year. In DALYs (Disability Adjusted Life Years), this adds up to 25 million with over 500,000 deaths annually (Herricks et al., 2017). The high disease burden combined with the threat of drug resistance calls for urgent and continuous drug discovery efforts for parasitic diseases (Cui et al., 2015; Nabarro et al., 2015; Vanaerschot et al., 2014).

**Fig. 1:**
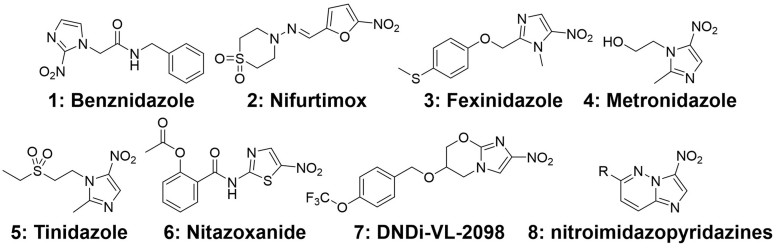
Nitro-group containing heterocyclic aromatic compounds with antiparasitic activities.

Heterocyclic aromatic nitro-compounds (imidazoles, furans and thiazoles, Fig. 1) are known for their potential in treating parasitic infections and their mode-of-action is considered to be the radical damage caused by the reactive and toxic species that are obtained from reduction of the nitro group by parasitic reductases (Ang et al., 2017). Benznidazole (**1**) and nifurtimox (**2**) are in clinical use for Chagas disease (Ribeiro et al., 2020). Fexinidazole (**3**) and nifurtimox combination with eflornithine (NECT), are used for treating the African sleeping sickness (de Koning, 2020) while metronidazole (**4**), tinidazole (**5**) and nitazoxanide (**6**) have a role in the treatment of giardiasis (Mørch and Hanevik, 2020). The compound DNDi-VL-2098 (**7**) is in development for visceral leishmaniasis (Gupta et al., 2015), while nitroimidazopyridazines (**8**) have been reported to be effective against a broad range of parasites growing under anaerobic or semi aerobic conditions (Tomcufcik et al., 1974).

**Fig. 2:**
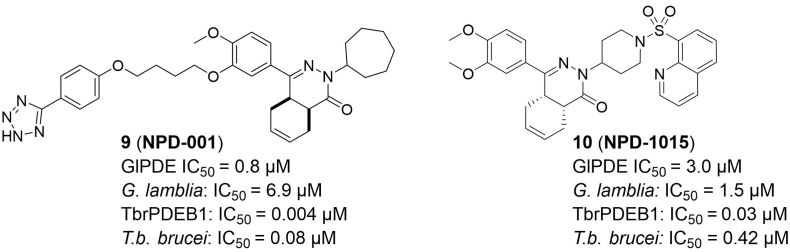
Tetrahydrophthalazinones **9**, **10** and their reported IC_50_ values against *G. lamblia* and *T. b. brucei* and relevant parasitic PDEs (Blaazer et al., 2018; Kunz et al., 2017b).

The 3′,5′-cyclic nucleotide phosphodiesterases (PDEs) have a critical role in cellular signal transduction: they hydrolyse the second messengers cyclic adenosine-3′,5′-monophosphate (cAMP) and cyclic guanosine-3′,5′-monophosphate (cGMP) to AMP and GMP, respectively. So far, eleven families of class-I PDEs were found (Maurice et al., 2014) and have been described as potential targets for the treatment of several parasitic diseases such as malaria (Howard et al., 2015) and Chagas disease (Sharon et al., 2010). Moreover, this class of enzymes has been proposed as potential therapeutic targets for sleeping sickness (de Koning et al., 2012; Oberholzer et al., 2007) and more recently also for giardiasis (Kunz et al., 2017a). For these last two parasitic diseases, tetrahydrophthalazinones were identified as an interesting hit series (**9** and **10**, Fig. 2), and initial hit optimization strategies towards parasitic PDE-selective compounds have been reported (Blaazer et al., 2018; Kunz et al., 2017a).

Previous studies have shown hybrid antimalarial compounds as a promising strategy to overcome the emergence of resistant parasite strains (Oliveira et al., 2015). Moreover, nitro-drugs have been reported to suffer from parasite resistance (Patterson and Wyllie, 2014), whereas resistance for hybrid anti-parasitic drugs has not been reported for PDE inhibitor scaffolds so far. In this study, we hypothesized that by merging a tetrahydrophthalazinone scaffold with a nitroimidazopyridazine (**8**), compounds with a putative dual mode-of-action could be generated, making them potentially more effective against a range of parasites. To test this hypothesis, we synthesized a series of hybrid molecules that were tested against the protozoal parasites including *G. lamblia*, *T. brucei*, *T. cruzi*, *L. infantum* and *P. falciparum*.

## 2 Materials and methods

### 2.1 Synthetic procedures

All chemicals were obtained from commercial suppliers without further purification. Reaction progress was monitored using thin-layer chromatography (TLC) and LC-MS analysis. For TLC analysis, Merck F254 alumina silica plates were used and visualized using UV. Silicycle UltraPure silica gel was used for manual purification columns. Automatic columns were performed using Biotage equipment. LC-MS analysis was performed on a Shimadzu LC-20AD liquid chromatograph pump system, equipped with an Xbridge (C18) 5 μm column (50 mm, 4.6 mm), connected to a Shimadzu SPD-M20A diode array detector, and MS detection using a Shimadzu LC-MS-2010EV mass spectrometer. The LC-MS conditions were as follows: solvent B (acetonitrile with 0.1% formic acid) and solvent A (water with 0.1% formic acid), flow rate of 1.0 mL/min, start with 5% B, linear gradient to 90% B in 4.5 min, then 1.5 min at 90% B, then linear gradient to 5% B in 0.5 min, then 1.5 min at 5% B; total run time of 8 min. Reverse-phase column chromatography purifications were performed using Buchi PrepChrom C-700 equipment with a discharge deuterium lamp ranging from 200 to 600 nm to detect compounds using solvent B (acetonitrile with 0.1% formic acid), solvent A (water with 0.1% formic acid), flow rate of 15.0 mL/min, and a gradient (start 95% A for 3.36 min, then linear gradient to 5% A in 30 min, then at 5% A for 3.36 min, then linear gradient to 95% A in 0.5 min, and then 1.5 min at 95% A). The purity of a compound was determined by calculating the peak area percentage by UV detection at 254 nm and was in all cases >95% except for some intermediates (indicated in the characterization). A Bruker 500 or 600 MHz spectrometer was used to record ^1^H, ^13^C and 2D NMR spectra. Chemical shifts (δ in ppm) and coupling constants (*J* in Hz) are reported with residual solvent as internal standard (δ ^1^H NMR, CDCl_3_ 7.26, CD_2_Cl_2_ 5.32, DMSO-*d*_6_ 2.50; δ ^13^C NMR, CDCl_3_ 77.16, CD_2_Cl_2_ 53.84, DMSO-*d*_6_ 39.52). Abbreviations used for ^1^H NMR descriptions are as follows: s = singlet, d = doublet, t = triplet, q = quintet, hept = heptet, dd = doublet of doublets, dt = doublet of triplets, tt = triplet of triplets, m = multiplet, app = apparent, br = broad signal. For HRMS analysis, a Bruker micrOTOF mass spectrometer was used using ESI in positive ion mode. All reactions were carried out under an inert nitrogen atmosphere.

#### 2.1.1 General methods

Method A: A mixture of 6-chloro-3-nitroimidazo[1,2-*b*]pyridazine (2.1 mmol, 0.42 g), an amine (2.0 mmol) and K_2_CO_3_ (4.7 mmol, 0.65 g) in DMF (20 mL) was heated for 2–24 h at 60 °C after which the mixture was poured into water. The aqueous mixture was extracted with DCM and the combined organic layers were dried over MgSO_4_ and evaporated. The residue was purified by silica gel column chromatography (eluents of EtOAc and *n*-heptane) or reverse phase chromatography.

Method B: A mixture of the benzyl chloride (1.0 mmol), **12c** (1.0 mmol, 0.52 g) and K_2_CO_3_ (2.0 mmol, 0.28 g) in DMF (15 mL) was heated for 4 h at 60 °C, after which the mixture was poured into water. The aqueous mixture was extracted with DCM and the combined organic layers were dried over MgSO_4_ and evaporated. The residue was purified by silica gel column chromatography (eluents of EtOAc and *n*-heptane).

##### 2.1.1.1 The intermediates are presented as supplemental data

***cis*-6-(3,4-Dimethoxybenzoyl)cyclohex-3-ene-1-carboxylic acid** (**11a**) and **(1*R*,6*S*)-6-(3,4-dimethoxybenzoyl)cyclohex-3-ene-1-carboxylic acid** (**11a’**) Prepared as described (van der Mey et al., 2002).

**(4a*S*,8a*R*)-4-(3,4-Dimethoxyphenyl)-2-(piperidin-4-yl)-4a,5,8,8a-tetrahydrophthalazin-1(2*H*)-one·HCl** (**11b**) Prepared as described (Sterk et al., 2004a).

***cis*-6-(3-(Cyclopentyloxy)-4-methoxybenzoyl)cyclohex-3-ene-1-carboxylic acid** (**12a**) Prepared as described (van der Mey et al., 2002).

***cis*-4-(3-(Cyclopentyloxy)-4-methoxyphenyl)-2-(piperidin-4-yl)-4a,5,8,8a-tetrahydrophthalazin-1(2*H*)-one·HCl** (**12b**) Prepared from **12a** (20 mmol, 6.9 g) and 4-hydrazinopiperidine·2HCl (20 mmol, 3.8 g) as described previously (van der Mey et al., 2002). Yield: 74%; LC-MS-ESI^+^ *m/z* 424 [M+H]^+^; purity 92%.

***cis*-4-(3-Hydroxy-4-methoxyphenyl)-2-(1-(3-nitroimidazo[1,2-*b*]pyridazin-6-yl)piperidin-4-yl)-4a,5,8,8a-tetrahydrophthalazin-1(2*H*)-one** (**12c**) Prepared from **12** by hydrolysing the cyclopentyl ether with 4-toluene sulfonic acid in a Dean-Stark apparatus as described previously (van der Mey et al., 2002). Yield 74%; LC-MS-ESI^+^ *m/z* 518 [M+H]^+^; purity 94%.

**4-(3,4-Dimethoxyphenyl)-4-oxobutanoic acid** (**18a**) Prepared as described (van der Mey et al., 2001).

**6*-*(3,4-Dimethoxyphenyl)-2-(piperidin-4-yl)-4,5-dihydropyridazin-3(2*H*)-one·HCl** (**18b**) Prepared as described (Sterk et al., 2004b).

**4-(3-Chloro-4-methoxyphenyl)-4-oxobutanoic acid** (**19a**) Prepared from 2-chloroanisole and succinic anhydride as described for the 3,4-dimethoxy analogue (van der Mey et al., 2002). Yield: 84%; LC-MS-ESI^+^ *m/z* 243 [M+H]^+^; purity 94%.

**6-(3-Chloro-4-methoxyphenyl)-2-(piperidin-4-yl)-4,5-dihydropyridazin-3(2*H*)-one·HCl** (**19b**) Prepared from 4-(3-chloro-4-methoxyphenyl)-4-oxobutanoic acid and 4-hydrazinopiperidine·2HCl as described for the 3,4-dimethoxy analogue (Sterk et al., 2004a); Yield: 67%; LC-MS-ESI^+^ *m/z* 322 [M+H]^+^; purity 91%.

##### 2.1.1.2 Final compounds (chemical analysis spectra shown in Figs. S1–S66)

**(4a*S*,8a*R*)-4-(3,4-Dimethoxyphenyl)-2-(1-(3-nitroimidazo[1,2-*b*]pyridazin-6-yl)piperidin-4-yl)-4a,5,8,8a-tetrahydrophthalazin-1(2*H*)-one** (**11**) Prepared from **11b** (2.0 mmol, 0.81 g) by method A; Yield 72%. ^1^H NMR (500 MHz, CDCl_3_) δ 8.39 (s, 1H), 7.85 (d, *J* = 10.0 Hz, 1H), 7.39–7.35 (m, 1H), 7.29–7.24 (m, 1H), 7.16 (d, *J* = 10.1 Hz, 1H), 6.85 (d, *J* = 8.4 Hz, 1H), 5.81 (app d, *J* = 9.9 Hz, 1H), 5.70 (app d, *J* = 10.0 Hz, 1H), 4.96 (tt, *J* = 11.4, 3.9 Hz, 1H), 4.53–4.39 (m, 2H), 3.90 (s, 3H), 3.82 (s, 3H), 3.37–3.30 (m, 1H), 3.25–3.12 (m, 2H), 3.06–2.98 (m, 1H), 2.80 (app t, *J* = 5.8 Hz, 1H), 2.32–2.16 (m, 3H), 2.10–1.95 (m, 2H), 1.91–1.84 (m, 2H). ^13^C NMR (126 MHz, CDCl_3_) δ 167.1, 155.7, 154.5, 151.0, 149.3, 137.7, 134.5, 134.3, 127.7, 126.5, 126.1, 124.0, 119.4, 113.9, 110.7, 108.4, 56.1, 56.0, 52.4, 45.61, 45.57, 34.9, 31.3, 29.5, 28.8, 23.5, 22.5. HR-MS: calc. for [M+H]^+^. 532.2303, found 532.2311.

***cis*-4-(3-(Cyclopentyloxy)-4-methoxyphenyl)-2-(1-(3-nitroimidazo[1,2-*b*]pyridazin-6-yl)piperidin-4-yl)-4a,5,8,8a-tetrahydrophthalazin-1(2*H*)-one** (**12**) Prepared from **12b** (2.2 mmol, 0.92 g) by method A. Purified by crystallization from MeOH. Yield: 84%; ^1^H NMR (500 MHz, CDCl_3_) δ 8.40 (s, 1H), 7.90 (d, *J* = 10.1 Hz, 1H), 7.36 (d, *J* = 2.0 Hz, 1H), 7.26–7.22 (m, 1H), 7.19 (d, *J* = 10.1 Hz, 1H), 6.84 (d, *J* = 8.4 Hz, 1H), 5.84–5.78 (m, 1H), 5.76–5.66 (m, 1H), 4.97 (tt, *J* = 11.6, 4.2 Hz, 1H), 4.77–4.69 (m, 1H), 4.52–4.40 (m, 2H), 3.86 (s, 3H), 3.31 (dt, *J* = 11.6, 5.8 Hz, 1H), 3.27–3.14 (m, 2H), 3.06–2.97 (m, 1H), 2.79 (app t, *J* = 5.9 Hz, 1H), 2.31–2.15 (m, 3H), 2.11–1.95 (m, 4H), 1.91–1.70 (m, 6H), 1.55–1.42 (m, 2H). ^13^C NMR (126 MHz, CDCl_3_) δ 167.1, 155.6, 154.6, 152.1, 147.8, 137.3, 133.4, 127.5, 126.2, 126.1, 124.1, 119.2, 114.1, 112.3, 111.3, 80.9, 56.2, 52.3, 45.6, 34.9, 32.91, 32.88, 31.3, 29.6, 28.9, 24.19, 24.17, 23.5, 22.5. HR-MS: calc. for [M+H]^+^. 586.2772, found 586.2775.

***cis*-4-(4-Methoxy-3-((3-nitrobenzyl)oxy)phenyl)-2-(1-(3-nitroimidazo[1,2-*b*]pyridazin-6-yl)piperidin-4-yl)-4a,5,8,8a-tetrahydrophthalazin-1(2*H*)-one** (**13**) Prepared from 3-nitrobenzylchloride (1.0 mmol, 0.17 g) by method B; Yield 45%. ^1^H NMR (500 MHz, CDCl_3_) δ 8.38 (s, 1H), 8.31 (s, 1H), 8.14 (d, *J* = 8.1 Hz, 1H), 7.89 (d, *J* = 10.0 Hz, 1H), 7.76 (d, *J* = 7.6 Hz, 1H), 7.55 (d, *J* = 7.9 Hz, 1H), 7.42 (s, 1H), 7.34–7.29 (m, 1H), 7.24 (d, *J* = 10.1 Hz, 1H), 6.91 (d, *J* = 8.5 Hz, 1H), 5.81 (app d, *J* = 9.3 Hz, 1H), 5.70 (app d, *J* = 9.5 Hz, 1H), 5.23 (s, 2H), 4.94 (ddd, *J* = 15.2, 11.2, 3.8 Hz, 1H), 4.53–4.40 (m, 2H), 3.95 (s, 3H), 3.29 (dt, *J* = 11.5, 5.7 Hz, 1H), 3.25–3.10 (m, 2H), 3.02 (app d, *J* = 17.1 Hz, 1H), 2.78 (app t, *J* = 5.8 Hz, 1H), 2.33–2.09 (m, 4H), 2.03–1.80 (m, 3H). ^13^C NMR (126 MHz, CDCl_3_) δ 167.0, 155.8, 154.0, 151.8, 148.5, 147.7, 139.3, 137.7, 134.5, 134.1, 133.4, 129.8, 127.7, 126.4, 126.1, 123.9, 123.2, 122.3, 120.6, 114.1, 112.0, 111.4, 70.4, 56.2, 52.3, 45.6, 45.5, 34.8, 31.2, 29.6, 28.9, 23.4, 22.4. HR-MS: calc. for [M+H]^+^. 653.2467, found 653.2477.

***cis*-4-(4-Methoxy-3-((3-methoxybenzyl)oxy)phenyl)-2-(1-(3-nitroimidazo[1,2-*b*]pyridazin-6-yl)piperidin-4-yl)-4a,5,8,8a-tetrahydrophthalazin-1(2*H*)-one** (**14**) Prepared from 3-methoxybenzylchloride (1.0 mmol, 0.16 g) by method B; Yield 51%. ^1^H NMR (500 MHz, CDCl_3_) δ 8.39 (s, 1H), 7.93 (d, *J* = 9.8 Hz, 1H), 7.41 (d, *J* = 1.9 Hz, 1H), 7.29–7.21 (m, 3H), 7.00–6.93 (m, 2H), 6.88 (d, *J* = 8.4 Hz, 1H), 6.80 (d, *J* = 8.0 Hz, 1H), 5.84–5.78 (m, 1H), 5.72–5.66 (m, 1H), 5.17–5.09 (m, 2H), 4.94 (tt, *J* = 11.4, 4.1 Hz, 1H), 4.55–4.42 (m, 2H), 3.93 (s, 3H), 3.78 (s, 3H), 3.30–3.13 (m, 3H), 3.06–2.98 (m, 1H), 2.77 (app t, *J* = 6.0 Hz, 1H), 2.28–2.17 (m, 2H), 2.15–2.06 (m, 1H), 2.04–1.78 (m, 4H). ^13^C NMR (126 MHz, CDCl_3_) δ 167.1, 160.0, 155.7, 154.2, 151.5, 148.2, 138.6, 137.5, 134.5, 133.7, 129.7, 127.5, 126.3, 126.0, 124.0, 119.7, 119.6, 114.1, 113.5, 113.0, 111.5, 111.1, 71.3, 56.2, 55.4, 52.2, 45.6, 45.5, 34.8, 31.2, 29.6, 28.9, 23.4, 22.4. HR-MS: calc. for [M+H]^+^. 638.2722, found 638.2731.

***cis*-4-(4-Methoxy-3-(pyridin-3-ylmethoxy)phenyl)-2-(1-(3-nitroimidazo[1,2-*b*]pyridazin-6-yl)piperidin-4-yl)-4a,5,8,8a-tetrahydrophthalazin-1(2*H*)-one** (**15**) Prepared from 3-(chloromethyl)pyridine·HCl (1.0 mmol, 0.16 g) by method B; Yield 42%. ^1^H NMR (500 MHz, CDCl_3_) δ 8.71 (s, 1H), 8.56 (d, *J* = 6.2 Hz, 1H), 8.36 (s, 1H), 7.98 (d, *J* = 7.9 Hz, 1H), 7.83 (d, *J* = 10.0 Hz, 1H), 7.51–7.46 (m, 1H), 7.43 (d, *J* = 2.0 Hz, 1H), 7.32–7.27 (m, 2H), 6.89 (d, *J* = 8.5 Hz, 1H), 5.83–5.77 (m, 1H), 5.71–5.65 (m, 1H), 5.19 (s, 2H), 4.93 (tt, *J* = 11.4, 4.2 Hz, 1H), 4.54–4.41 (m, 2H), 3.91 (s, 3H), 3.32–3.26 (m, 1H), 3.23–3.12 (m, 2H), 3.05–2.98 (m, 1H), 2.77 (app t, *J* = 5.8 Hz, 1H), 2.27–2.11 (m, 3H), 2.06–1.92 (m, 3H), 1.88–1.82 (m, 1H). ^13^C NMR (126 MHz, CDCl_3_) δ 167.0, 155.8, 153.9, 151.7, 147.6, 146.8, 146.1, 138.1, 137.9, 134.9, 134.6, 134.3, 127.7, 126.7, 126.1, 124.6, 123.9, 120.7, 114.1, 112.1, 111.4, 68.6, 56.2, 52.4, 45.6, 34.8, 31.2, 29.7, 28.9, 23.4, 22.4. HR-MS: calc. for [M+H]^+^. 609.2568, found 609.2587.

**c*is*-4-(4-Methoxy-3-((2-methoxybenzyl)oxy)phenyl)-2-(1-(3-nitroimidazo[1,2-*b*]pyridazin-6-yl)piperidin-4-yl)-4a,5,8,8a-tetrahydrophthalazin-1(2*H*)-one** (**16**) Prepared from 2-methoxybenzylchloride (1.0 mmol, 0.16 g) by method B; Yield 72%. ^1^H NMR (500 MHz, CDCl_3_) δ 8.37 (s, 1H), 7.89 (d, *J* = 10.1 Hz, 1H), 7.40 (d, *J* = 7.4 Hz, 1H), 7.35 (s, 1H), 7.32 (d, *J* = 8.5 Hz, 1H), 7.25–7.16 (m, 2H), 6.92 (app t, *J* = 7.5 Hz, 1H), 6.90–6.82 (m, 2H), 5.81 (d, *J* = 9.7 Hz, 1H), 5.69 (d, *J* = 9.7 Hz, 1H), 5.26–5.17 (m, 2H), 4.93 (tt, *J* = 11.3, 3.8 Hz, 1H), 4.50 (app d, *J* = 13.4 Hz, 1H), 4.41 (app d, *J* = 13.3 Hz, 1H), 3.92 (s, 3H), 3.81 (s, 3H), 3.29–3.23 (m, 1H), 3.23–3.11 (m, 2H), 3.02 (app d, *J* = 16.7 Hz, 1H), 2.78 (app t, *J* = 5.8 Hz, 1H), 2.28–2.08 (m, 4H), 2.05–1.80 (m, 3H). ^13^C NMR (126 MHz, CDCl_3_) δ 167.0, 156.8, 155.6, 154.4, 151.5, 148.4, 137.5, 134.5, 133.8, 129.1, 128.9, 127.5, 126.3, 126.0, 125.1, 124.1, 120.8, 119.4, 113.8, 111.7, 111.3, 110.4, 66.2, 56.2, 55.6, 52.2, 45.41, 45.39, 34.8, 31.2, 29.5, 28.9, 23.4, 22.4. HR-MS: calc. for [M+H]^+^. 638.2722, found 638.2737.

**c*is*-4-(4-Methoxy-3-(pyridin-2-ylmethoxy)phenyl)-2-(1-(3-nitroimidazo[1,2-*b*]pyridazin-6-yl)piperidin-4-yl)-4a,5,8,8a-tetrahydrophthalazin-1(2*H*)-one** (**17**) Prepared from 2-chloromethylpyridine·HCl (1.0 mmol, 0.16 g) by method B; Yield 62%. ^1^H NMR (500 MHz, CD_2_Cl_2_) δ 8.49 (d, *J* = 4.7 Hz, 1H), 8.31 (s, 1H), 7.83 (d, *J* = 10.1 Hz, 1H), 7.74 (app t, *J* = 6.5 Hz, 1H), 7.54 (d, *J* = 6.8 Hz, 1H), 7.47 (d, *J* = 2.0 Hz, 1H), 7.30 (dd, *J* = 8.5, 2.0 Hz, 1H), 7.23 (app d, *J* = 10.0 Hz, 2H), 6.89 (d, *J* = 8.5 Hz, 1H), 5.79–5.74 (m, 1H), 5.71–5.65 (m, 1H), 5.20 (s, 2H), 4.90 (tt, *J* = 11.5, 4.0 Hz, 1H), 4.52–4.40 (m, 2H), 3.88 (s, 3H), 3.35–3.27 (m, 1H), 3.22–3.10 (m, 2H), 2.97–2.89 (m, 1H), 2.76 (app t, *J* = 5.9 Hz, 1H), 2.26–2.08 (m, 3H), 2.04–1.89 (m, 3H), 1.84–1.78 (m, 1H). ^13^C NMR (126 MHz, CD_2_Cl_2_) δ 167.1, 157.1, 156.1, 154.1, 151.6, 149.0, 148.3, 138.5, 137.8, 134.9, 134.8, 128.0, 126.9, 126.2, 124.4, 123.3, 122.2, 120.1, 114.2, 111.44, 111.40, 71.6, 56.3, 52.5, 45.8, 45.7, 35.1, 31.3, 29.7, 29.1, 23.5, 22.7. HR-MS: calc. for [M+H]^+^. 609.2568, found 609.2583.

**6-(3,4-Dimethoxyphenyl)-2-(1-(3-nitroimidazo[1,2-*b*]pyridazin-6-yl)piperidin-4-yl)-4,5-dihydropyridazin-3(2*H*)-one** (**18**) Prepared from **18b** (2.0 mmol, 0.71 g) by method A; Yield 72%. ^1^H NMR (500 MHz, CDCl_3_) δ 8.39 (s, 1H), 7.83 (d, *J* = 10.1 Hz, 1H), 7.32 (d, J = 2.3, 1H), 7.22 (dd, *J* = 8.4, 2.3 Hz, 1H), 7.16 (d, *J* = 9.9 Hz, 1H), 6.85 (d, *J* = 8.5 Hz, 1H), 4.94 (tt, *J* = 11.6, 4.2 Hz, 1H), 4.46 (d, *J* = 13.4 Hz, 2H), 3.90 (s, 3H), 3.82 (s, 3H), 3.24–3.13 (m, 3H), 2.97–2.86 (m, 2H), 2.61 (app t, *J* = 8.2 Hz, 2H), 2.15 (app qd, J = 12.6, 4.4, 2H), 1.93 (app dd, *J* = 13.5, 3.9 Hz, 2H). ^13^C NMR (126 MHz, CDCl_3_) δ 165.3, 155.6, 150.9, 150.6, 149.1, 137.9, 134.7, 128.6, 126.6, 119.4, 113.8, 110.6, 108.6, 77.2, 56.1, 56.0, 52.1, 45.6, 29.2, 27.6, 22.4. HR-MS: calc. for [M+H]^+^. 480.1990, found 480.1990.

**6-(3-Chloro-4-methoxyphenyl)-2-(1-(3-nitroimidazo[1,2-*b*]pyridazin-6-yl)piperidin-4-yl)-4,5-dihydropyridazin-3(2*H*)-one** (**19**) Prepared from **19b** (2.0 mmol, 0.72 g) by method A; Yield 72%. ^1^H NMR (500 MHz, CDCl_3_) δ 8.40 (s, 1H), 7.89 (d, *J* = 10.0 Hz, 1H), 7.78–7.74 (m, 1H), 7.56 (dd, *J* = 8.6, 1.7 Hz, 1H), 7.19 (d, *J* = 10.1 Hz, 1H), 6.91 (d, *J* = 8.7 Hz, 1H), 4.95 (tt, *J* = 11.5, 3.8 Hz, 1H), 4.48 (app d, *J* = 13.4 Hz, 2H), 3.92 (s, 3H), 3.17 (app t, *J* = 12.5 Hz, 2H), 2.89 (t, *J* = 8.2 Hz, 2H), 2.62 (t, *J* = 8.2 Hz, 2H), 2.15 (app td, *J* = 12.5, 3.9 Hz, 2H), 1.92 (app d, *J* = 11.1 Hz, 2H). ^13^C NMR (126 MHz, CDCl_3_) δ 165.0, 156.2, 155.5, 149.1, 137.4, 133.7, 132.5, 129.0, 127.7, 126.3, 125.5, 122.9, 113.9, 111.6, 56.3, 52.0, 45.3, 29.1, 27.3, 22.2. HR-MS: calc. for [M+H]^+^. 484.1495, found 484.1500.

**6-(3,4-Dimethoxyphenyl)-2-(1-(imidazo[1,2-*b*]pyridazin-6-yl)piperidin-4-yl)-4,5-dihydropyridazin-3(2*H*)-one** (**20**) Prepared from **18b** (2.0 mmol, 0.71 g) and 6-chloroimidazo[1,2-*b*]pyridazine (2.1 mmol, 0.32 g) by method A, but heating at 60 °C for 24 h; Yield 19%. ^1^H NMR (600 MHz, CDCl_3_) δ 7.89 (d, *J* = 9.9 Hz, 1H), 7.69 (s, 1H), 7.58 (s, 1H), 7.35 (d, *J* = 1.9 Hz, 1H), 7.24 (dd, *J* = 8.4, 2.0 Hz, 1H), 6.97 (d, *J* = 10.0 Hz, 1H), 6.87 (d, *J* = 8.4 Hz, 1H), 4.94 (tt, *J* = 11.6, 4.1 Hz, 1H), 4.29 (app d, *J* = 13.4 Hz, 2H), 3.92 (s, 3H), 3.79 (s, 3H), 3.16–3.05 (m, 2H), 2.93 (t, *J* = 8.2 Hz, 2H), 2.69–2.58 (m, 2H), 2.17 (app qd, *J* = 12.6, 4.1 Hz, 2H), 1.94–1.85 (m, 2H). ^13^C NMR (151 MHz, CDCl_3_) δ 165.3, 155.2, 150.9, 150.4, 149.2, 135.6, 129.9, 128.6, 125.3, 119.3, 116.7, 111.6, 110.7, 108.5, 56.1, 55.9, 52.2, 46.1, 29.1, 27.6, 22.3. HR-MS: calc. for [M+H]^+^. 435.2139, found 435.2153.

***N*,*N*-Dimethyl-3-nitroimidazo[1,2-*b*]pyridazin-6-amine** (**21**) Prepared from dimethylamine hydrochloride by method A; Yield 71%. ^1^H NMR (500 MHz, CDCl_3_) δ 8.37 (s, 1H), 7.82 (d, *J* = 10.0 Hz, 1H), 7.03 (d, *J* = 10.0 Hz, 1H), 3.22 (s, 6H). ^13^C NMR (126 MHz, CDCl_3_) δ 155.8, 137.7, 134.5, 134.2, 126.3, 112.8, 38.7. HR-MS: calc. for [M+H]^+^. 208.0829, found 208.0829.

**3-Nitro-6-(pyrrolidin-1-yl)imidazo[1,2-*b*]pyridazine** (**22**) Prepared from pyrrolidine by method A; Yield 65%. ^1^H NMR (500 MHz, CDCl_3_) δ 8.35 (s, 1H), 7.78 (d, *J* = 9.9 Hz, 1H), 6.86 (d, *J* = 9.9 Hz, 1H), 3.63–3.54 (m, 4H), 2.13–2.04 (m, 4H). ^13^C NMR (126 MHz, CDCl_3_) δ 153.8, 138.0, 134.4, 134.0, 126.2, 113.8, 47.4, 25.5. HR-MS: calc. for [M+H]^+^. 234.0986, found 234.0996. Spectral data are in agreement with a previous report (Sridhar et al., 2017).

**3-Nitro-6-(piperidin-1-yl)imidazo[1,2-*b*]pyridazine** (**23**) Prepared from piperidine by method A; Yield 61%. ^1^H NMR (600 MHz, CDCl_3_) δ 8.37 (s, 1H), 7.81 (d, *J* = 10.0 Hz, 1H), 7.12 (d, *J* = 10.0 Hz, 1H), 3.65 (app s, 4H), 1.72 (app s, 6H). ^13^C NMR (151 MHz, CDCl_3_) δ 156.1, 138.0, 134.8, 134.4, 126.4, 114.2, 47.3, 25.7, 24.6. HR-MS: calc. for [M+H]^+^. 248.1142, found 248.1149. Spectral data are in agreement with a previous report (Sridhar et al., 2017).

**6-(4-Methylpiperazin-1-yl)-3-nitroimidazo[1,2-*b*]pyridazine formate** (**24**) Prepared from *N*-methylpiperazine by method A; Yield 45%. ^1^H NMR (500 MHz, CDCl_3_) δ 8.41 (s, 1H), 8.24 (s, 1H), 7.88 (d, *J* = 10.0 Hz, 1H), 7.10 (d, *J* = 10.0 Hz, 1H), 5.42 (s, 1H), 3.81 (t, *J* = 5.2 Hz, 4H), 2.84 (t, *J* = 5.1 Hz, 4H), 2.51 (s, 3H). ^13^C NMR (126 MHz, CDCl_3_) δ 165.5, 155.5, 138.0, 134.9, 133.9, 127.0, 113.4, 53.5, 45.0, 44.8. HR-MS: calc. for [M+H]^+^. 263.1251, found 263.1257.

**3-Nitro-6-(4-(pyrrolidin-1-yl)piperidin-1-yl)imidazo[1,2-*b*]pyridazine formate** (**25**) Prepared from 4-(pyrrolidin-1-yl)-piperidine by method A; Yield 46%. ^1^H NMR (500 MHz, CDCl_3_) δ 8.43 (s, 1H), 8.39 (s, 1H), 7.84 (d, *J* = 10.0 Hz, 1H), 7.11 (d, *J* = 10.0 Hz, 1H), 6.11 (s, 1H), 4.41 (app d, *J* = 13.6 Hz, 2H), 3.17 (app s, 4H), 3.02 (app t, *J* = 12.1 Hz, 3H), 2.20 (app d, *J* = 12.2 Hz, 2H), 2.09–1.90 (m, 6H). ^13^C NMR (126 MHz, CDCl_3_) δ 167.9, 155.4, 138.0, 135.0, 134.6, 126.9, 113.7, 61.5, 50.6, 44.9, 28.5, 23.4. HR-MS: calc. for [M+H]^+^. 317.1721, found 317.1732.

**3-Nitro-6-(4-phenylpiperidin-1-yl)imidazo[1,2-*b*]pyridazine** (**26**) Prepared from 4-phenylpiperidine by method A; Yield 46%. ^1^H NMR (500 MHz, CDCl_3_) δ 8.41 (s, 1H), 7.87 (d, *J* = 10.0 Hz, 1H), 7.35 (t, *J* = 7.6 Hz, 2H), 7.27–7.23 (m, 3H), 7.20 (d, *J* = 10.1 Hz, 1H), 4.50 (app d, *J* = 13.4 Hz, 2H), 3.16 (app td, *J* = 13.1, 2.1 Hz, 2H), 2.86 (tt, *J* = 12.2, 3.4 Hz, 1H), 2.06 (app d, *J* = 13.1 Hz, 2H), 1.90–1.79 (m, 2H). ^13^C NMR (126 MHz, CDCl_3_) δ 155.8, 145.1, 137.6, 134.5, 133.8, 128.8, 126.9, 126.8, 126.3, 114.2, 46.9, 42.6, 32.8. HR-MS: calc. for [M+H]^+^. 324.1455, found 324.1440.

**3-Nitro-6-(4-phenylpiperazin-1-yl)imidazo[1,2-*b*]pyridazine** (**27**) Prepared from *N*-phenylpiperazine by method A; Yield 49%. ^1^H NMR (500 MHz, CDCl_3_) δ 8.41 (s, 1H), 7.87 (d, *J* = 10.0 Hz, 1H), 7.32 (app t, *J* = 7.8 Hz, 2H), 7.16 (d, *J* = 10.0 Hz, 1H), 7.02 (app d, *J* = 6.2 Hz, 2H), 6.95 (t, *J* = 6.9 Hz, 1H), 3.86 (app s, 4H), 3.41–3.33 (m, 4H). ^13^C NMR (126 MHz, CDCl_3_) δ 155.7, 150.9, 138.1, 134.9, 134.6, 129.5, 126.9, 120.8, 116.7, 113.4, 49.2, 45.9. HR-MS: calc. for [M+H]^+^. 325.1408, found 325.1405. Spectral data are in agreement with a previous report (Sridhar et al., 2017).

**6-(4-Benzylpiperidin-1-yl)-3-nitroimidazo[1,2-*b*]pyridazine** (**28**) Prepared from 4-benzylpiperidine by method A; Yield 67%. ^1^H NMR (500 MHz, CDCl_3_) δ 8.37 (s, 1H), 7.79 (d, *J* = 10.0 Hz, 1H), 7.34–7.28 (m, 2H), 7.22 (t, *J* = 7.4 Hz, 1H), 7.16 (d, *J* = 7.4 Hz, 2H), 7.10 (d, *J* = 10.0 Hz, 1H), 4.31 (app d, *J* = 13.6 Hz, 2H), 3.02–2.90 (m, 2H), 2.59 (d, *J* = 6.8 Hz, 2H), 1.91–1.78 (m, 3H), 1.40–1.28 (m, 2H). ^13^C NMR (126 MHz, CDCl_3_) δ 155.7, 139.9, 137.7, 134.2, 129.2, 128.5, 126.31, 126.26, 114.0, 77.2, 46.4, 43.1, 38.1, 31.6. HR-MS: calc. for [M+H]^+^. 338.1612, found 338.1614.

**(1-(3-Nitroimidazo[1,2-*b*]pyridazin-6-yl)piperidin-4-yl)(phenyl)methanone** (**29**) Prepared from 4-benzoylpiperidine by method A; Yield 59%. ^1^H NMR (500 MHz, CDCl_3_) δ 8.39 (s, 1H), 7.97 (d, *J* = 7.3 Hz, 2H), 7.86 (d, *J* = 10.0 Hz, 1H), 7.60 (t, *J* = 7.4 Hz, 1H), 7.51 (t, *J* = 7.7 Hz, 2H), 7.17 (d, *J* = 10.1 Hz, 1H), 4.36 (app d, *J* = 13.5 Hz, 2H), 3.60 (tt, *J* = 10.8, 3.8 Hz, 1H), 3.32–3.20 (m, 2H), 2.12–2.02 (m, 2H), 2.00–1.87 (m, 2H). ^13^C NMR (126 MHz, CDCl_3_) δ 201.7, 155.7, 137.8, 135.7, 134.5, 134.3, 133.5, 129.0, 128.4, 126.5, 114.0, 45.8, 43.1, 28.0. HR-MS: calc. for [M+H]^+^. 352.1404, found 352.1417.

***N*-(4-Methoxybenzyl)-*N*-methyl-3-nitroimidazo[1,2-*b*]pyridazin-6-amine** (**30**) Prepared from *N*-(4-methoxybenzyl)methylamine by method A; Yield 62%. ^1^H NMR (500 MHz, CDCl_3_) δ 8.38 (s, 1H), 7.79 (d, *J* = 10.0 Hz, 1H), 7.28 (d, *J* = 8.5 Hz, 2H), 6.99 (d, *J* = 10.0 Hz, 1H), 6.87 (d, *J* = 8.5 Hz, 2H), 4.74 (s, 2H), 3.79 (s, 3H), 3.26 (s, 3H). ^13^C NMR (126 MHz, CDCl_3_) δ 159.3, 155.4, 137.7, 134.4, 134.1, 128.9, 128.6, 126.4, 114.3, 113.1, 55.4, 53.9, 37.3. HR-MS: calc. for [M+H]^+^. 314.1248, found 314.1247.

**6-(Piperidin-1-yl)imidazo[1,2-*b*]pyridazine** (**31**) Prepared from piperidine and 6-chloroimidazo[1,2-*b*]pyridazine instead of 6-chloro-3-nitroimidazo[1,2-*b*]pyridazine by method A, but heating at 60 °C for 24 h; Yield 21%. ^1^H NMR (500 MHz, CDCl_3_) δ 7.68 (s, 1H), 7.66 (s, 1H), 7.51 (s, 1H), 6.81 (d, *J* = 9.9 Hz, 1H), 3.47 (app s, 4H), 1.67 (app s, 6H). ^13^C NMR (126 MHz, CDCl_3_) δ 155.4, 136.4, 131.7, 125.8, 116.6, 110.7, 47.6, 25.5, 24.6. HR-MS: calc. for [M+H]^+^. 203.1291, found 203.1291.

### 2.2 *In vitro* tests

*G. lamblia* trophozoites (WBC6) were seeded into 96-well-plates in 0.2 mL Keister-medium containing 10^3^ trophozoites per well in the presence of the compounds dissolved in dimethyl sulfoxide (DMSO, final concentration 0.25%) or with DMSO as a solvent control and incubated for 72 h at 37 °C in an anaerobic chamber (80% N_2_, 10% H_2_, 10% CO_2_). Then, plates were centrifuged (2 min, 1000 rpm), medium was removed, and cells were washed once with PBS containing 1 g/L glucose. Then, PBS-glucose containing 10 mg/L resazurin was added, and plates were incubated for up to 5 h at 37 °C. Fluorescence reading (ex 530 nm/em 590 nm) was performed at various time points, and the linear increase of fluorescence was used as a marker for cell viability. IC_50_ values were calculated using the logit-log algorithm and are given as mean values with 95%-confidence intervals (Müller et al., 2020; Müller and Hemphill, 2013; Müller and Müller, 2019). The assays for growth inhibition of *T. b. brucei*, *T. cruzi*, *L. infantum, P. falciparum* and MRC-5 (human lung fibroblasts MRC-5_SV40_) were previously described (Blaazer et al., 2014). PDE activity was determined exactly as described (de Heuvel et al., 2021; Kunz et al., 2005, 2017b). All assays were carried out in triplicates and no more than 20% substrate was hydrolyzed in all reactions. Compounds were dissolved in DMSO and the final DMSO concentration was 1% in all reaction mixes. Control reactions with DMSO alone were always included. Data were analyzed using the GraphPad Prism software package (v7.0, GraphPad, San Diego).

## 3 Results and discussion

### 3.1 Synthesis of 3-nitroimidazo[1,2-*b*]pyridazine analogues

**Scheme 1:**
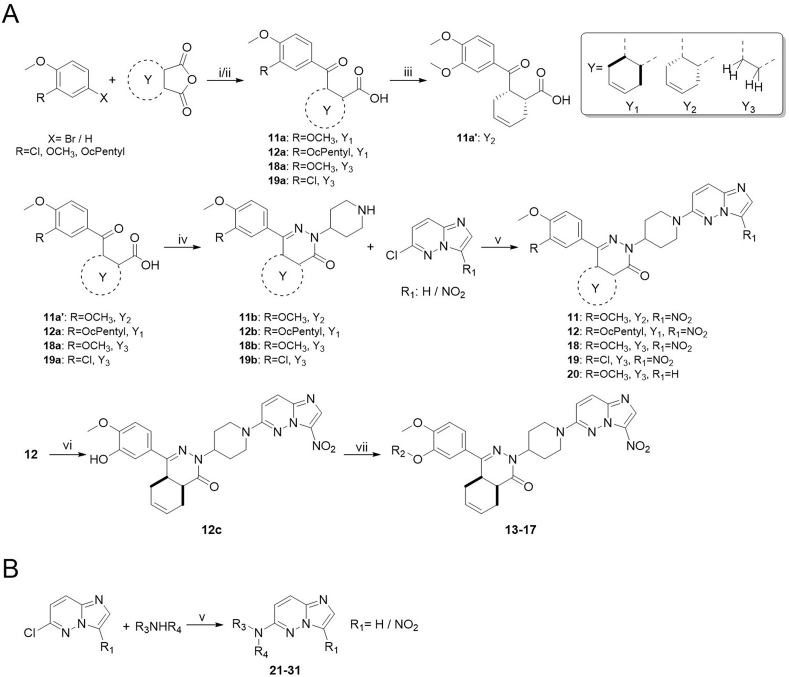
Synthesis of analogues **11**-**31**. Reagents and conditions: i for **11a**, **18a**, **19a**: AlCl_3_, DCM, rt, 4h; ii for **12a**: Mg, THF, rt followed by 2 h reflux; iii: crystallization (×3) with (*S*)-(−)-α-methylbenzylamine from EtOAc; iv: 4-hydrazinopiperidine·2HCl, TEA, EtOH, reflux, 16 h; v: DMF, K_2_CO_3_, 60 °C, 2–24 h; vi: Dean-Stark, 4-toluenesulfonic acid, toluene, reflux, 4 h; vii: R_2_Cl, K_2_CO_3_, DMF, 60 °C, 4 h.

Compounds **11**-**20** were prepared according to Scheme 1A. In the case R is MeO or Cl, X is H and the sequence started with a Friedel–Crafts acylation (step i) using succinic anhydride or tetrahydrophthalic anhydride. If R is a cyclopentyloxy group, X is Br and the sequence started with a Grignard reaction (step ii). In both cases γ-ketocarboxylic acids were obtained. Enantiomeric acid **11a’** was obtained through resolution with (*S*)-(−)-α-methylbenzylamine in EtOAc from **11a** (step iii). The next step (iv), condensation with 4-hydrazinopiperidine, was performed in refluxing ethanol and resulted in the substituted piperidines, which were further *N*-substituted with an imidazopyridazine in DMF using K_2_CO_3_ to scavenge the HCl. As described previously (van der Mey et al., 2002), the cyclopentyl group of **12** can be hydrolyzed selectively using 4-toluenesulfonic acid in a Dean-Stark apparatus and the resulting phenol **12c** was substituted with commercially available benzyl chlorides or pyridylmethyl chlorides under standard conditions. All final compounds were prepared as racemic mixtures of the *cis*-diastereomers (4a*S*,8a*R* and 4a*R*,8a*S*) except for compound **11** (4a*S*, 8a*R*). Scheme 1B depicts the synthesis of 6-amino-imidazopyridazine analogues (**21**-**31**) using conditions essentially identical to step v in Scheme 1A.

### 3.2 Antiparasitic activities of 3-nitroimidazo[1,2-*b*]pyridazine analogues

As PDE inhibitors and nitro heterocyclic compounds both have therapeutic potential in protozoal infections, we combined the tetrahydrophthalazinone scaffold with a nitroimidazopyridazine aiming for two modes of action into one hybrid molecule with improved efficacy. For this purpose, we first prepared the tetrahydrophthalazinones **11**-**17** with variations in R_2_, the *meta* position of the phenyl ring, and tested them against the panel of protozoa and for cytotoxicity on the human MRC-5 cell line (using tamoxifen as positive control) together with the benchmark compounds metronidazole, miltefosine, chloroquine, benznidazole and suramin. Activities of control antiparasitic agents were within previously described ranges (Bénéré et al., 2007; El Sayed et al., 2009; van Baelen et al., 2008; Venkatraj et al., 2014) and the results for **11**-**20** are summarized in Table 1 with representative dose-response curves shown in Fig. S67. All compounds show excellent anti-*Giardia* activity and no or very weak toxicity (Table 1) with high selectivity over other parasites (Table S1). Notably, all these novel tetrahydrophthalazinones have much higher anti-*Giardia* activity than the clinically used metronidazole (IC_50_ = 0.8 μM): a 40-fold (chlorine derivative **19**, IC_50_ = 19.0 nM) to an exceptional >1,000-fold (methoxy and 2-pyridylmethoxy derivatives **11** and **17**, IC_50_ = 0.5 nM for both).

**Table 1:**
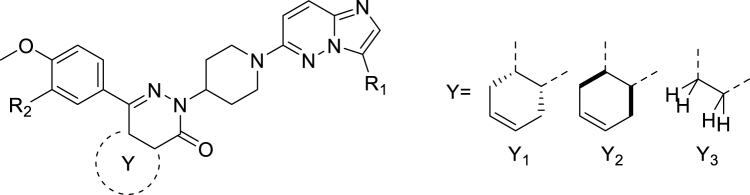
Anti-protozoal activity and toxicity of pyridazines and tetrahydrophthalazinones.

Cmpd	R_1_	R_2_	Y	M.W.	cLogP^a^	tPSA^a^	IC_50_ (nM)	
							*G.l.*^b^	MRC-5^c^
**11**	NO_2_	MeO	Y_1_	531.6	3.3	127.7	0.5 ± 0.2	>6.4 × 10^4^
**12**	NO_2_	cyclopentyloxy	Y_2_	585.7	4.6	127.7	9.3 ± 0.6	>6.4 × 10^4^
**13**	NO_2_		Y_2_	652.7	5.0	170.8	1.2 ± 0.4	>6.4 × 10^4^
**14**	NO_2_		Y_2_	637.7	4.9	136.9	1.0 ± 0.5	>6.4 × 10^4^
**15**	NO_2_		Y_2_	608.7	3.8	140.6	6.8 ± 1.5	15,000 ± 11,000
**16**	NO_2_		Y_2_	637.7	4.9	136.9	2.8 ± 0.8	49,000 ± 21,000
**17**	NO_2_		Y_2_	608.7	3.9	140.6	0.5 ± 0.1	>6.4 × 10^4^
**18**	NO_2_	MeO	Y_3_	479.5	2.1	127.7	0.2 ± 0.1	>6.4 × 10^4^
**19**	NO_2_	Cl	Y_3_	483.9	2.9	118.5	19.0 ± 3.2	>6.4 × 10^4^
**20**	H	MeO	Y_3_	434.5	2.1	84.5	930 ± 100	>6.4 × 10^4^
**3** (**MET**)	-	-	-	171.2	−0.5	81.2	800 ± 100	-
**Tamoxifen**	-	-	-	371.5	6.8	12.5	-	11,000 ± 5,000

MET: metronidazole; *G.l.*: *Giardia lamblia*.

a cLogP, tPSA are calculated using Collaborative Drug Discovery (CDD) Vault.

b mean values ± standard errors are given for quadruplicates, the standard errors correspond to the 95% confidence intervals as calculated via the logit-log algorithm.

c mean values ± standard deviations, n ≥ 2.

As mentioned earlier, tetrahydrophthalazinones are known PDE inhibitors of selected human (*e.g.* human PDE4) and parasitic enzymes (*e.g.* GlPDE). To investigate if PDE inhibition is contributing to the high anti-*Giardia* activity of **11**, we determined its activity as a GlPDE inhibitor (Table 2). Moreover, in light of the known interaction of tetrahydrophthalazinones with human PDE4, also the activity against the off-target human PDE4 (Houslay et al., 2005; Manallack et al., 2005; Tenor et al., 2011) was measured. As can be seen in Table 2, **11** is a moderate inhibitor of GlPDE (*K*_i_ = 0.5 μM, Fig. S69), but is 3,000-fold more active against human PDE4 (*K*_i_ = 0.16 nM, Fig. S68). As such potent human PDE4 inhibition will prevent any further development because of foreseen unwanted adverse effects (Souness et al., 2000; Tenor et al., 2011), we aimed at modifying the scaffold in such a way that the anti-*Giardia* activity would be retained while the potency against human PDE4 would be strongly reduced. Based on expert knowledge from previous structure-activity relationship (SAR) studies for PDE4 inhibition (van der Mey et al., 2001, van der Mey et al., 2002; Veerman et al., 2016), we decided to remove the cyclohexene ring Y_1_/Y_2_ (Table 1) and replace one of the ethers (R_2_, Table 1) by a chlorine, resulting in the pyridazinones **18** and **19**. Removing the cyclohexene ring as in **18** resulted in a 2.5-fold increase in anti-*Giardia* potency (Table 1, IC_50_ = 0.2 nM), while the hPDE4 affinity was 20-fold decreased (Table 2). An additional replacement of the *meta*-methoxy group by a chlorine (**19**) decreased the anti-*Giardia* activity 100-fold while the affinity for hPDE4B was 60-fold decreased compared to **18**. In this series of tetrahydrophthalazonones/pyridazinones, **18** is our most potent anti-*Giardia* compound with an IC_50_ value on growth inhibition of ∼200 pM.

**Table 2:**
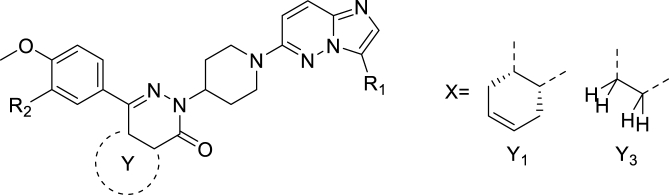
PDE inhibiting activity of selected compounds.

Cmpd	R_1_	R_2_	Y	M.W.	cLogP^a^	tPSA^a^	*G.l.* IC_50_ (nM)^b^	GlPDE *K*_i_ (nM)^c^	hPDE4B *K*_i_ (nM)^c^
**11**	NO_2_	MeO	Y_1_	531.6	3.3	127.7	0.5 ± 0.2	500 ± 300	0.16 ± 0.02
**18**	NO_2_	MeO	Y_3_	479.5	2.1	127.7	0.2 ± 0.1	>10^4^	3.2 ± 0.4
**19**	NO_2_	Cl	Y_3_	483.9	2.9	118.5	19.0 ± 3.2	>10^6^	200 ± 50
**20**	H	MeO	Y_3_	434.5	2.1	84.5	930 ± 100	>10^6^	5.0 ± 1.0

a cLogP, tPSA are calculated using CDD Vault.

b mean values ± standard errors are given for quadruplicates, the standard errors correspond to the 95% confidence intervals as calculated via the logit-log algorithm.

c mean values ± standard deviations, n ≥ 2.

For the compounds **11**, **18**–**20** we also measured their GlPDE activities (Table 2). From these data, one can conclude that it is highly unlikely that inhibition of this PDE significantly contributes to the anti-parasitic efficacy of these compounds against *Giardia*. This is especially evident for our most potent compound **18**, with only a *K*_i_ value of >10 μM on GlPDE, which is over 50,000-fold more than the IC_50_ value (0.2 nM) for *Giardia* growth inhibition.

To find out if this class of compounds has any other activity, independent of the nitro group, which might contribute to their anti-*Giardia* efficacy, we decided to prepare **20**, an analogue of **18** lacking the nitro group. As **20** is 4,500-fold less active than its nitro analogue **18**, we concluded that the main mode-of-action of **18** is directly related to the nitro group. The remaining anti-*Giardia* activity of **20** (IC_50_ = 0.93 μM) that is equipotent to metronidazole is also not related to GlPDE inhibition, since also **20** shows no GlPDE inhibition (Table 2), indicating that PDE inhibition should not be regarded as an additional mode-of-action of these anti-*Giardia* compounds. Meanwhile, imidazo[1,2-*b*]pyridazines have been previously reported as kinase inhibitors for *P. falciparum* and *Toxoplasma gondii*, which could serve as a potential mechanism for the remaining activity of **20** (Garrido et al., 2021)*.*

To better understand the potential of nitroimidazopyridazines in designing novel and potent compounds with anti-*Giardia* activity, the relatively “simple” analogues in Table 3 lacking the tetrahydrophthalazinone part associated with the off-target PDE4 inhibition were prepared and tested for antiparasitic efficacy and cytotoxicity (Table 3). Surprisingly, also in this series analogues with nano- to picomolar IC_50_ values against *G. lamblia* were identified. None of the analogues showed relevant activity against the other protozoa (Table S1). Anti-*Giardia* activities range from 144 nM (**24**, NR_3_R_4_ = *N*-methylpiperazine) to 500 pM (**28**, NR_3_R_4_ = 4-benzylpiperidine). Also for this series, the importance of the nitro group was investigated. Compound **31**, the non-nitro analogue of **23**, was prepared and proved to be inactive (Table 3). The difference between both compounds in inhibiting growth of *G. lamblia* is larger than 2,500 folds, providing strong evidence that the nitro group is of main importance for the activity of this scaffold. Like the compounds in Table 1, also the compounds in Table 3 have negligible toxicity on the human cell line MRC-5.

**Table 3:**
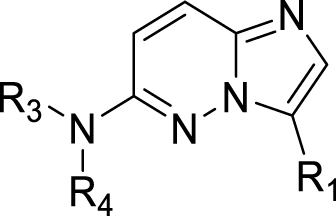
Antiprotozoal activity and toxicity of 6-substituted imidazo[1,2-*b*]pyridazines.

Cmpd	R_1_	NR_3_R_4_	M.W.	cLogP^a^	tPSA^a^	IC_50_ (nM)	
						*G.l.*^b^	MRC-5^c^
**21**	NO_2_		207.2	1.4	76.6	22.0 ± 3.0	>6.4 × 10^4^
**22**	NO_2_		233.2	1.8	76.6	2.3 ± 0.4	>6.4 × 10^4^
**23**	NO_2_		247.3	2.2	76.6	3.6 ± 0.8	28,100 ± 1,400
**24**^d^	NO_2_		308.3	1.2	79.8	144.0 ± 31.0	>6.4 × 10^4^
**25**^d^	NO_2_		316.4	1.8	79.8	5.8 ± 0.9	>6.4 × 10^4^
**26**	NO_2_		323.4	3.7	76.6	8.2 ± 1.0	>6.4 × 10^4^
**27**	NO_2_		324.3	3.1	79.8	0.7 ± 0.1	>6.4 × 10^4^
**28**	NO_2_		337.4	4.1	76.6	0.5 ± 0.1	7,960 ± 210
**29**	NO_2_		351.4	3.2	93.6	1.1 ± 0.2	>6.4 × 10^4^
**30**	NO_2_		313.3	2.9	85.8	10.3 ± 1.5	>6.4 × 10^4^
**31**	H		202.3	2.2	33.4	>10^4^	>6.4 × 10^4^
**2** (**MET**)	-		171.2	−0.5	81.2	800 ± 100	-

a cLogP, tPSA are calculated using CDD Vault.

b mean values ± standard errors are given for quadruplicates, the standard errors correspond to the 95% confidence intervals as calculated via the logit-log algorithm.

c mean values ± standard deviations, n ≥ 2.

d formate.

The importance of physicochemical properties of potential new drugs has become a mainstay in drug development since the formulation of the Lipinski rule of 5 (Ro5) (Lipinski et al., 1997). Still, the use of this Ro5 should be limited to oral availability of systemically active drugs. Moreover, it was clearly stated by McKerrow and Lipinski that this Ro5 is not applicable to potential anti-parasitic drugs (McKerrow and Lipinski, 2017). Despite these limitations of the Ro5, for our project we are convinced that physicochemical properties for targeting gastrointestinal parasites are important. Though the optimal values are not known, to our knowledge, it seems plausible that low lipophilicity (cLogP), low polar surface area (tPSA) and low molecular weight must be important, *e.g.* for proper solubility and membrane passage to enter parasites. Therefore, we believe that the result from this study, compounds which combine high anti-parasite potency with good calculated physicochemical properties (cLogP: 1.2–4.1; tPSA: 77-94 Å; molecular weight: 202.3–351.4 Dalton) warrants detailed evaluation of this class of compounds for their potential in the treatment of giardiasis. Importantly, the known nitro-resistance of a number of parasites, including *Giardia lamblia* (Nabarro et al., 2015), might limit the ultimate usefulness of this class of compounds and warrants future studies with resistant parasite strains.

In summary, we have identified novel nitroimidazopyrimidine compounds that have nanomolar to picomolar activity against *G. lamblia* without significant toxicity against human MRC-5 cells. Though this study was set up to find novel compounds with dual activity, their potency seems to rely mainly on the nitro group. In view of their good drug-like properties, especially low lipophilicity, low polar surface area and the low molecular weight, 3-nitroimidazo[1,2-*b*]pyridazines are promising candidates for further lead optimization studies.

## Declaration of competing interest

The authors declared that there is no conflict of interest.
