# Supplementary material for: 3-nitroimidazo[1,2-b]pyridazine as a novel scaffold for antiparasitics with sub-nanomolar anti-Giardia lamblia activity
Source: Int J Parasitol Drugs Drug Resist. 2022 May 26;19:47–55. doi: 10.1016/j.ijpddr.2022.05.004 (PMC9213561; doi:10.1016/j.ijpddr.2022.05.004)
Supplement: Multimedia component 1 [file mmc1.docx]

Supplementary data^[[1]](#footnote-2)^

3-nitroimidazo[1,2-*b*]pyridazine as a novel scaffold for antiparasitics with sub-nanomolar anti-*Giardia lamblia* activity

Yang Zheng ^a,1^, Joachim Müller ^b,1^, Stefan Kunz ^a,b^, Marco Siderius ^a^, Louis Maes ^c^, Guy Caljon ^c^, Norbert Müller ^b^, Andrew Hemphill ^b^, Geert Jan Sterk ^a^, Rob Leurs ^a,^*

^a^ Amsterdam Institute for Molecules, Medicines and Systems, Division of Medicinal Chemistry, Faculty of Science, Vrije Universiteit Amsterdam, De Boelelaan 1108, 1081 HZ Amsterdam, the Netherlands

^b^ Vetsuisse Faculty, Institute of Parasitology, University of Bern, Länggass-Strasse 122, CH-3012 Bern, Switzerland

^c^ Laboratory of Microbiology, Parasitology and Hygiene (LMPH), University of Antwerp, Universiteitsplein 1, 2610 Wilrijk, Belgium

^1^ These authors contributed equally.

* Corresponding author. E-mail address: r.leurs@vu.nl (R. Leurs).

**Table of contents**

Final compound chemical analyses (Figs. S1-S66) P2-P46

Representative dose-response curves of G. lamblia, hPDE4, GlPDE assays (Figs. S67-S69) P47-P48

Phenotypic activity of **11**-**31** against *T. brucei, T. cruzi, L. infantum, P. falciparum* and MRC-5 (Table S1) P49-P50


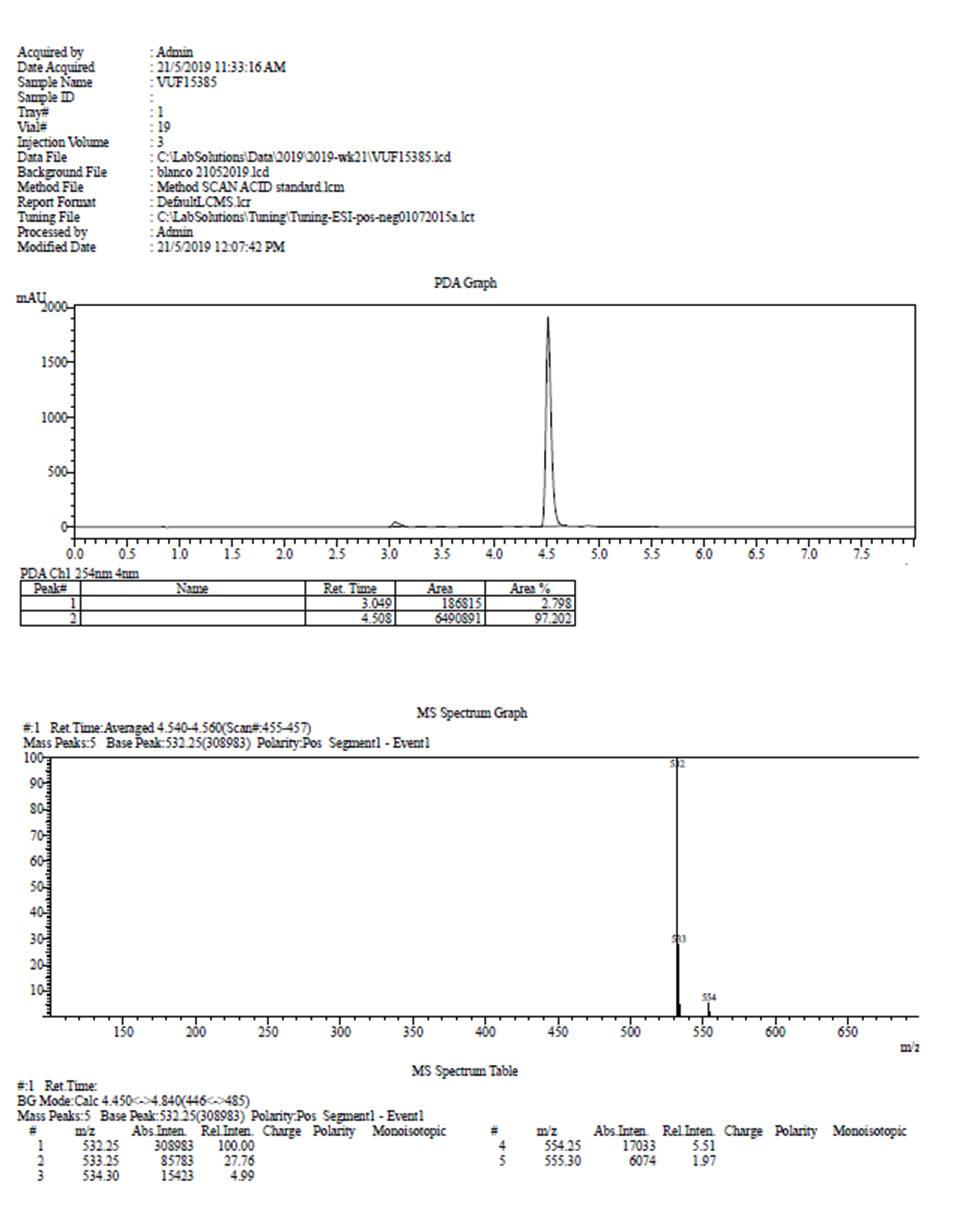


Figure S1. LCMS spectrum of compound **11**


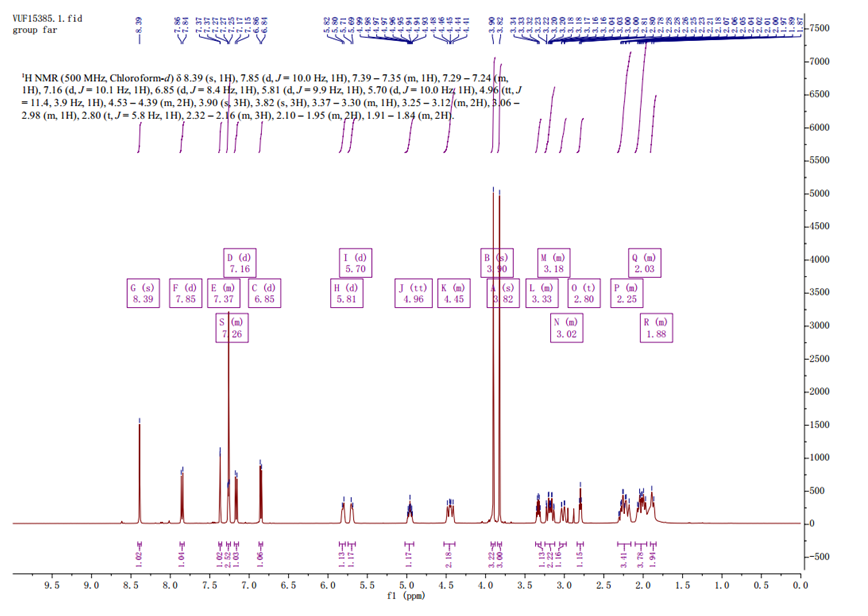


Figure S2. ^1^H NMR spectrum of compound **11** in CDCl_3_


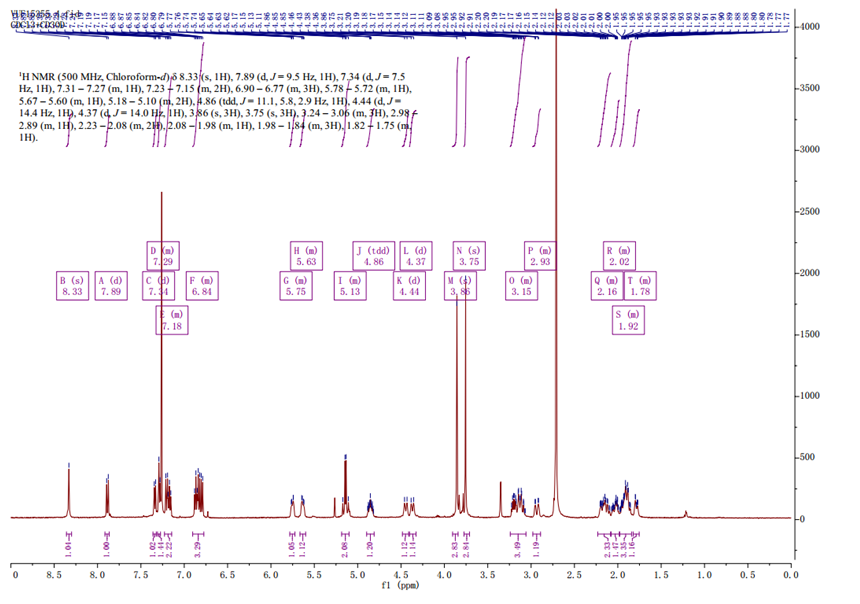


Figure S3. ^1^H NMR spectrum of compound **11** in CDCl_3_ + CD_3_OD

**
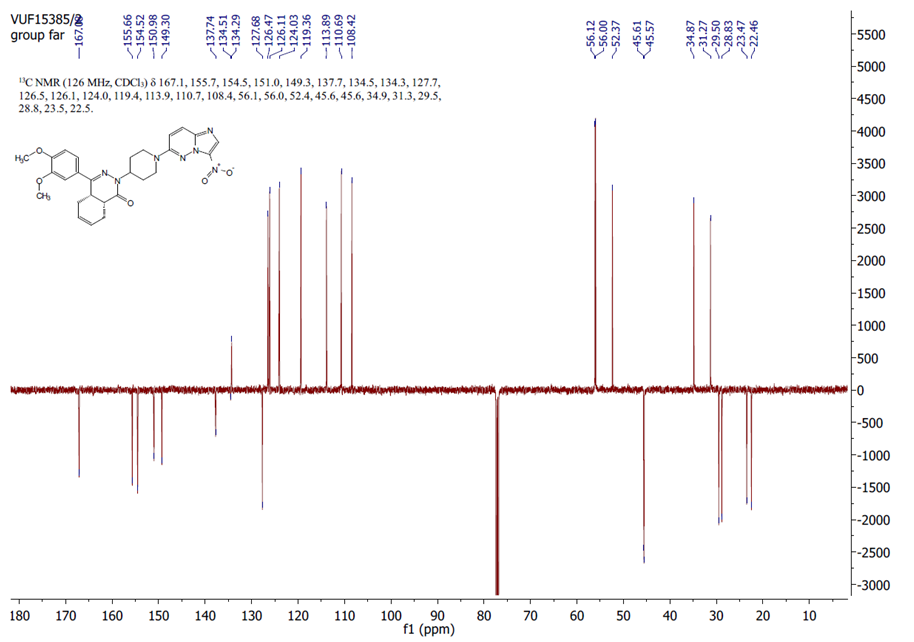
**

Figure S4. ^13^C NMR spectrum of compound **11**


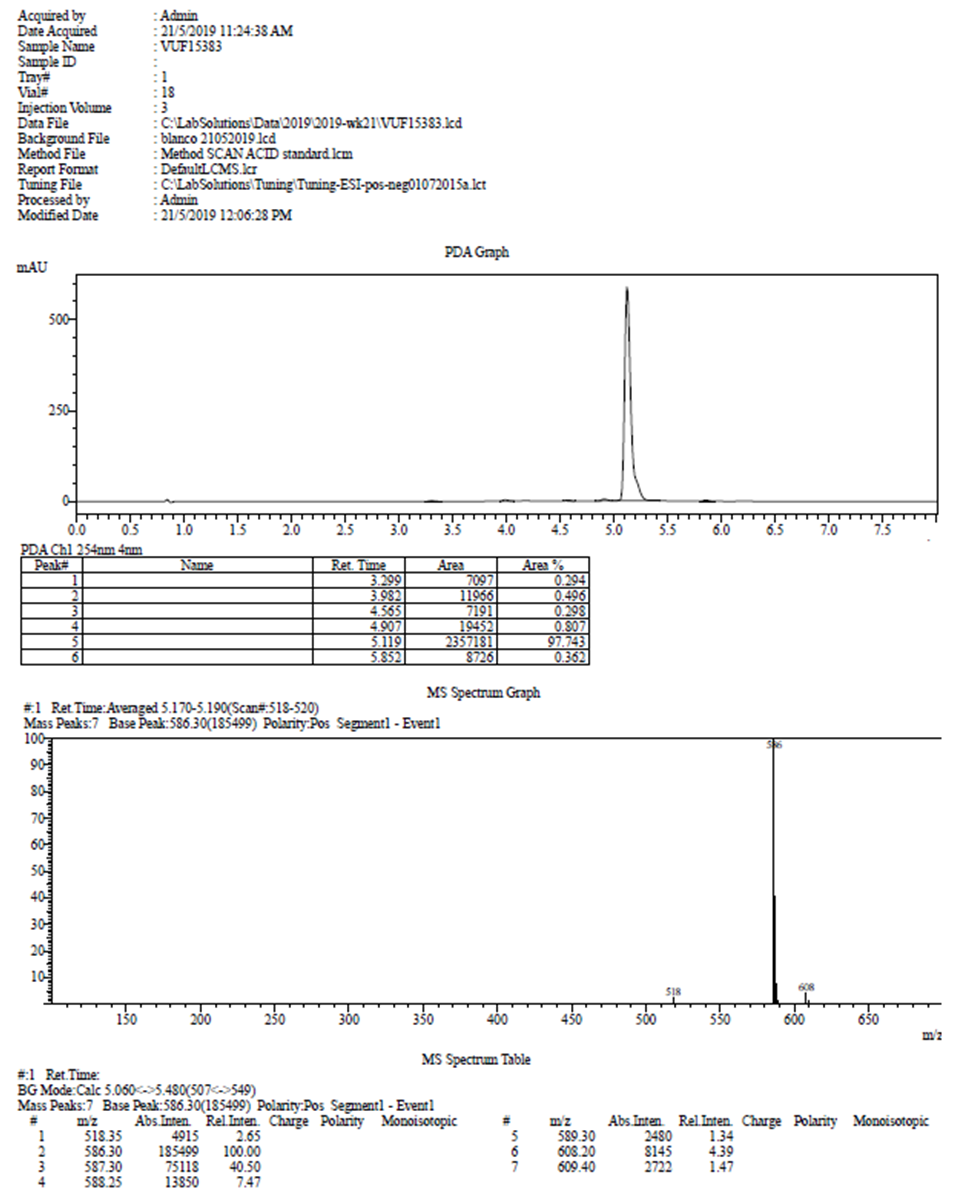


Figure S5. LCMS spectrum of compound **12**


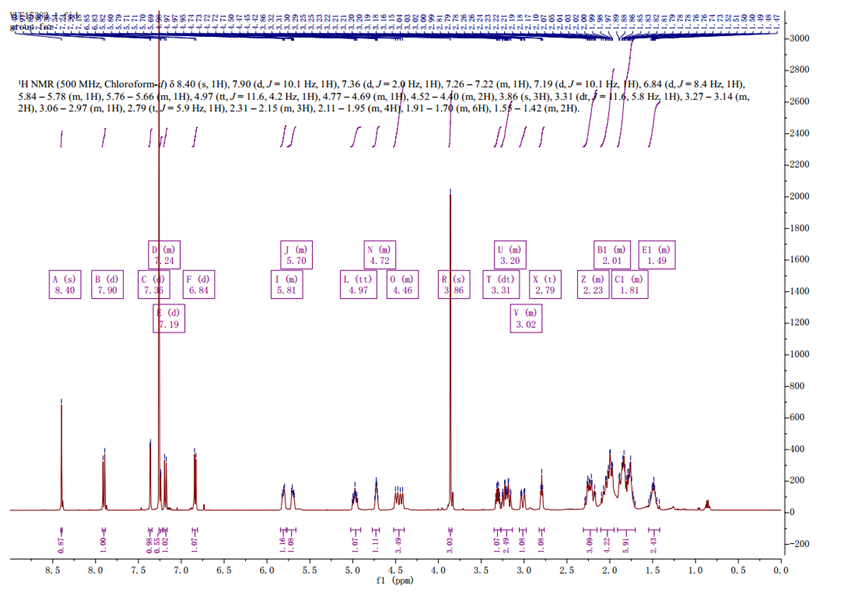


Figure S6. ^1^H NMR spectrum of compound **12**

**
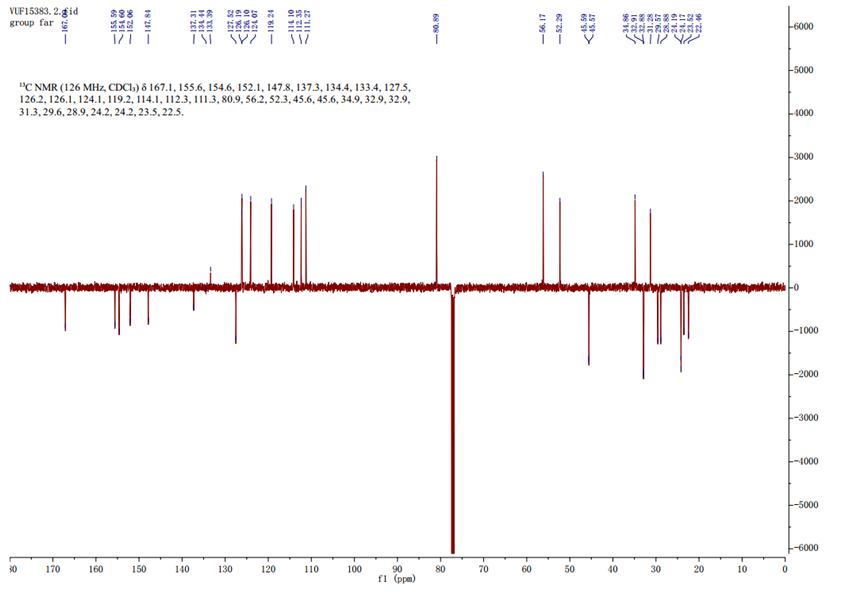
**

Figure S7. ^13^C NMR spectrum of compound **12**


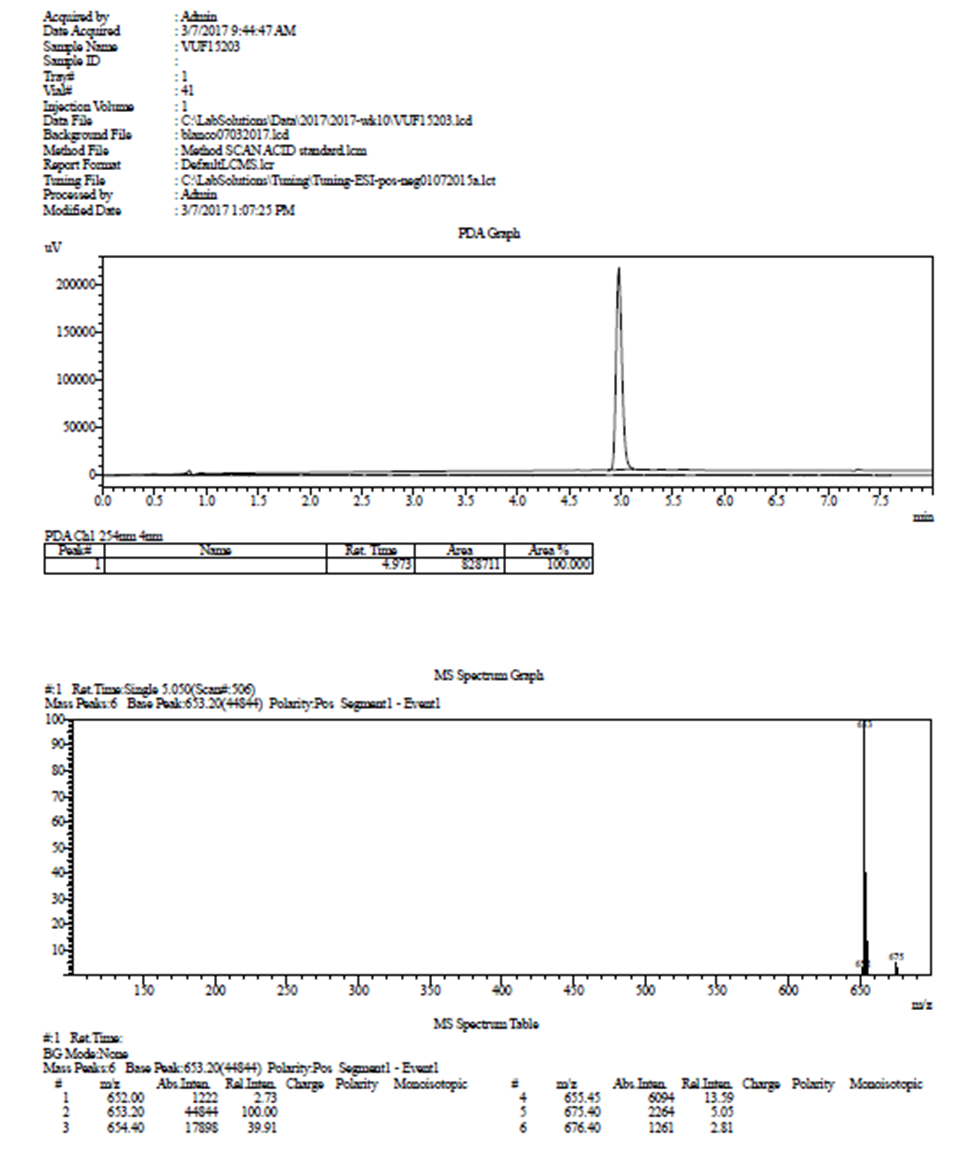
Figure S8. LCMS spectrum of compound **13**


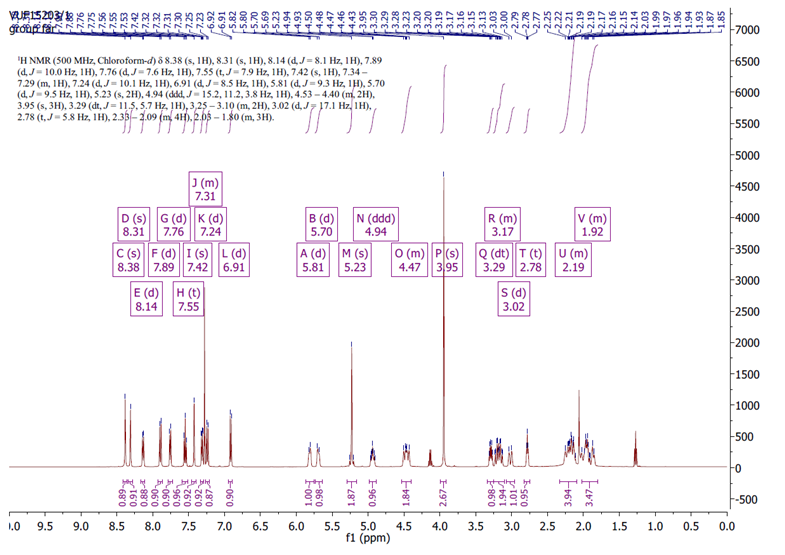


Figure S9. ^1^H NMR spectrum of compound **13**

**
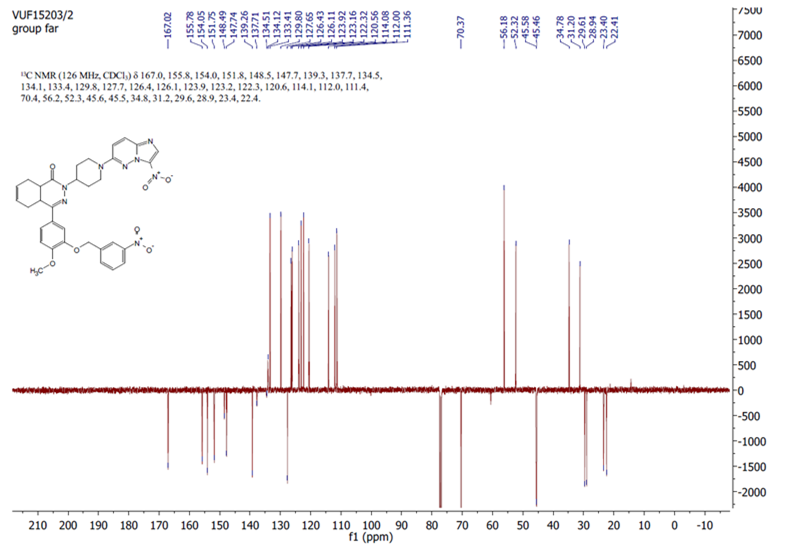
**

Figure S10. ^13^C NMR spectrum of compound **13**


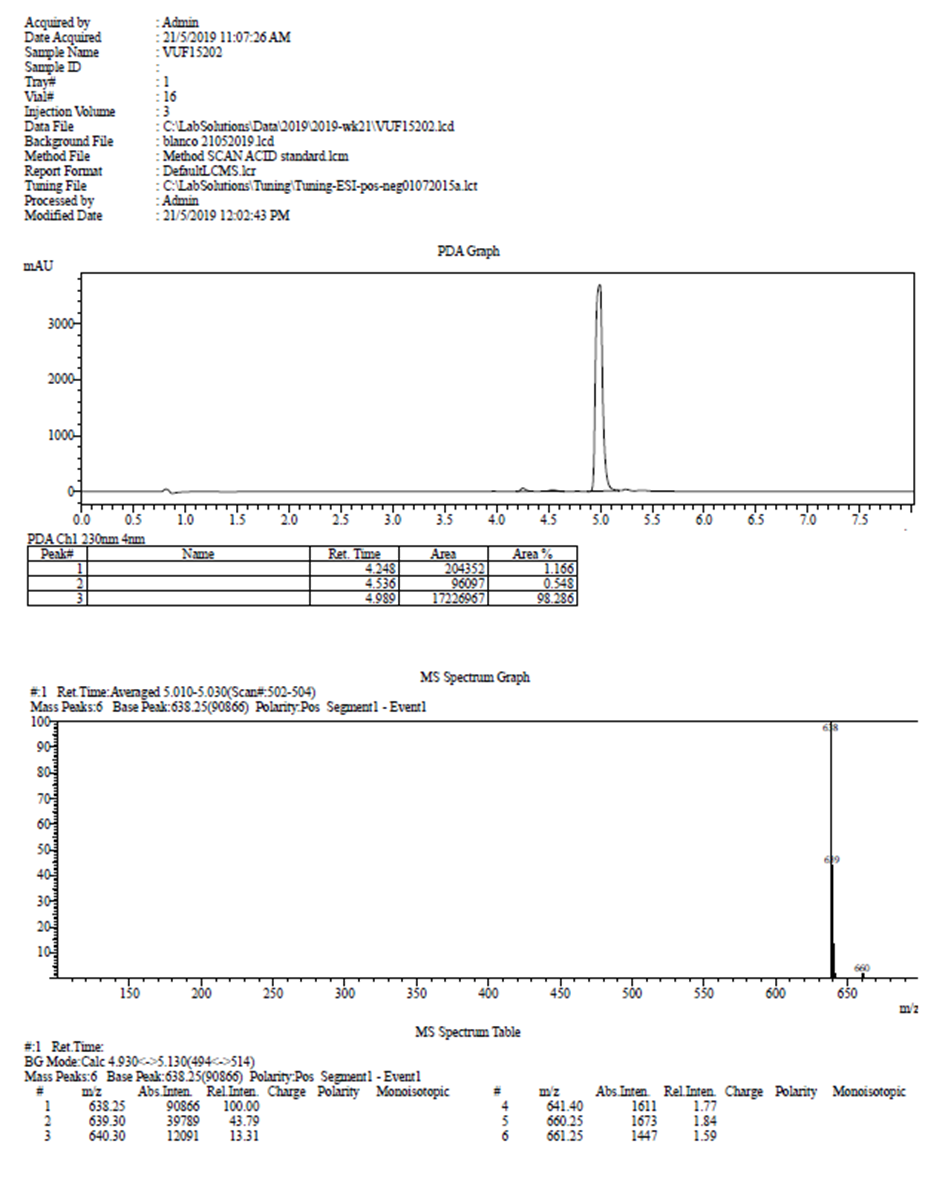


Figure S11. LCMS spectrum of compound **14**


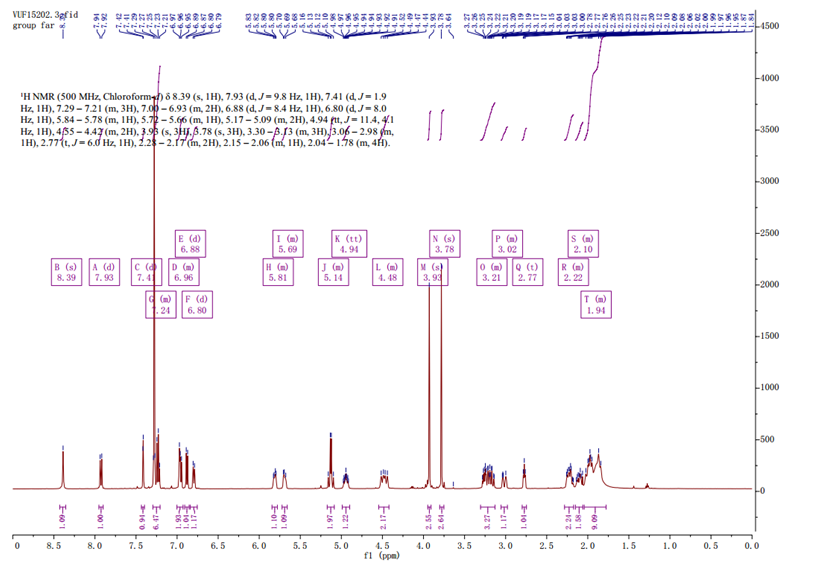


Figure S12. ^1^H NMR spectrum of compound **14** in CDCl_3_

**
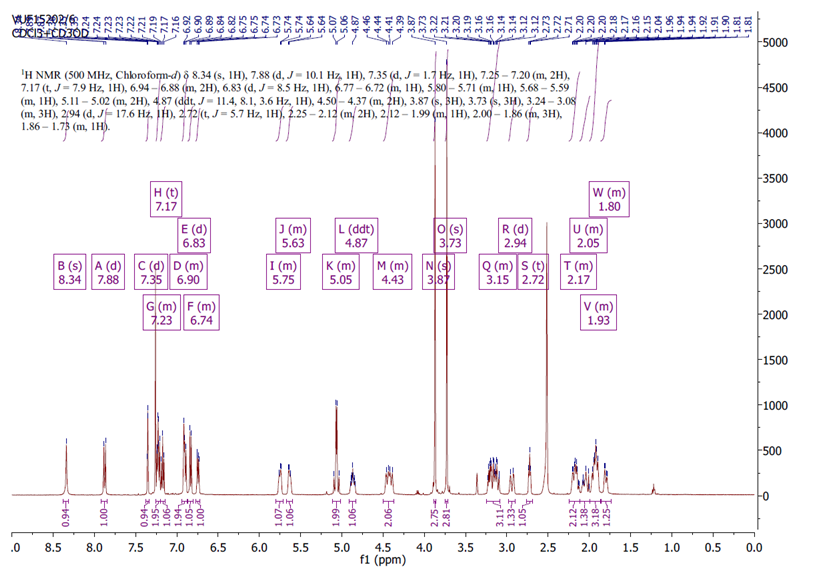
**

Figure S13. ^1^H NMR spectrum of compound **14** in CDCl_3_ + CD_3_OD

**
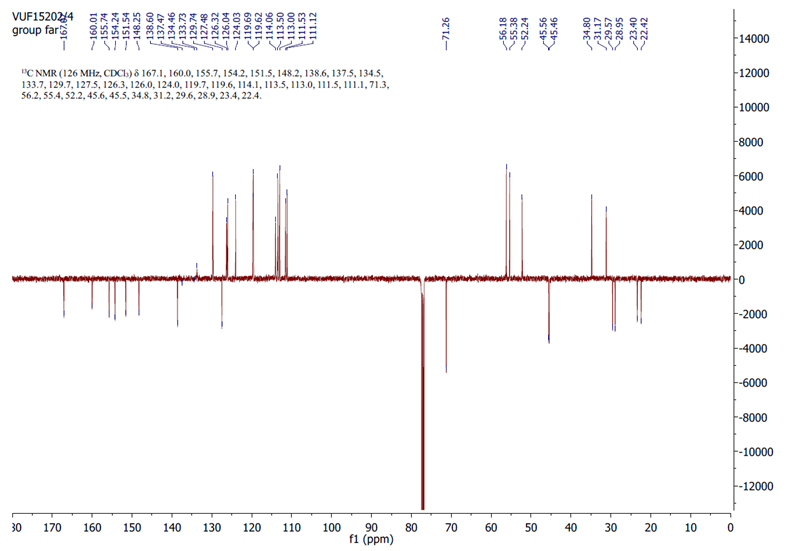
**

Figure S14. ^13^C NMR spectrum of compound **14**


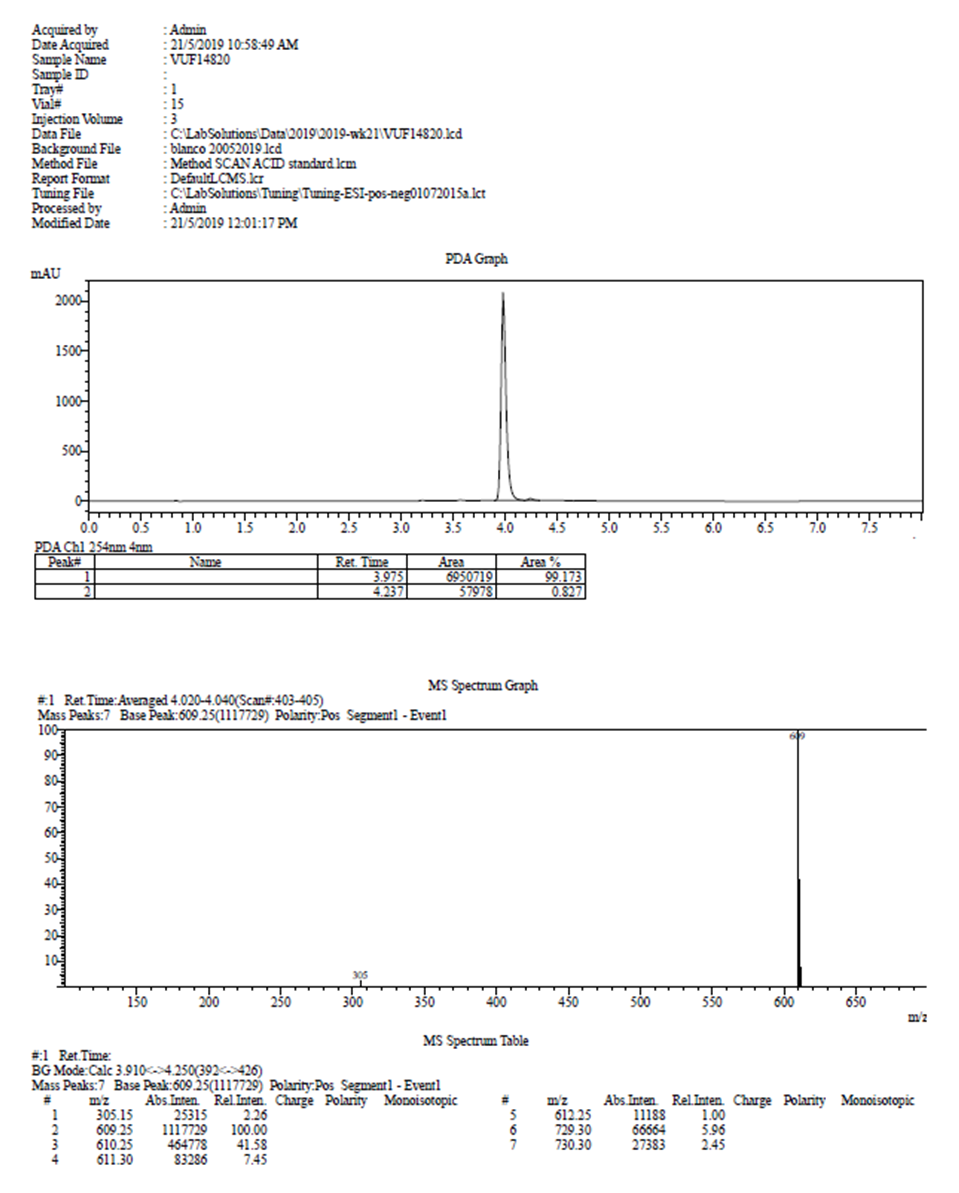


Figure S15. LCMS spectrum of compound **15**

**
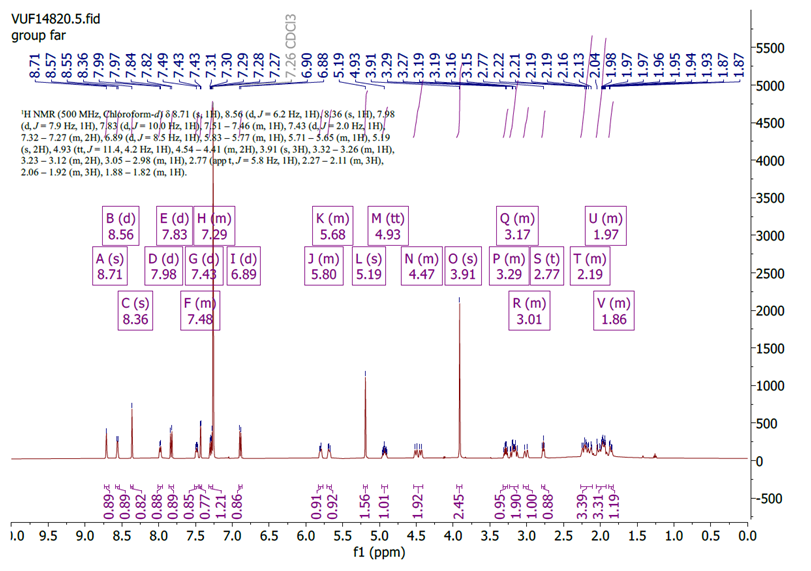
**

Figure S16. ^1^H NMR spectrum of compound **15**

**
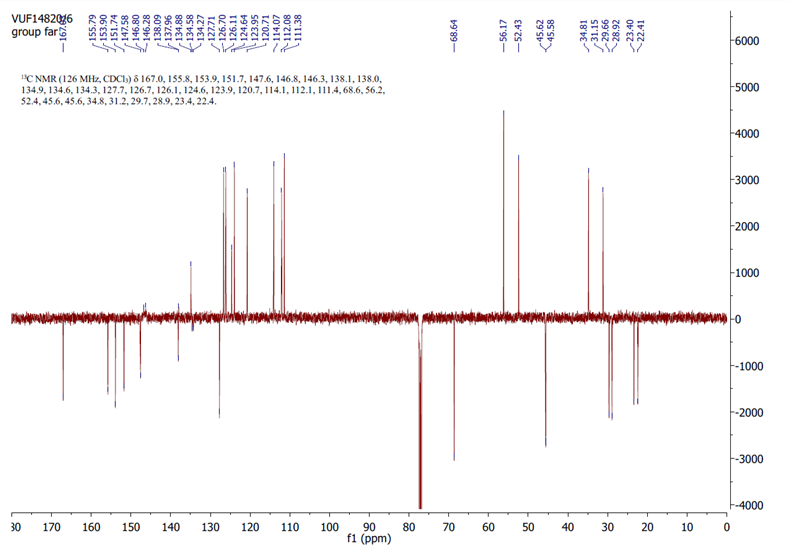
**

Figure S17. ^13^C NMR spectrum of compound **15**


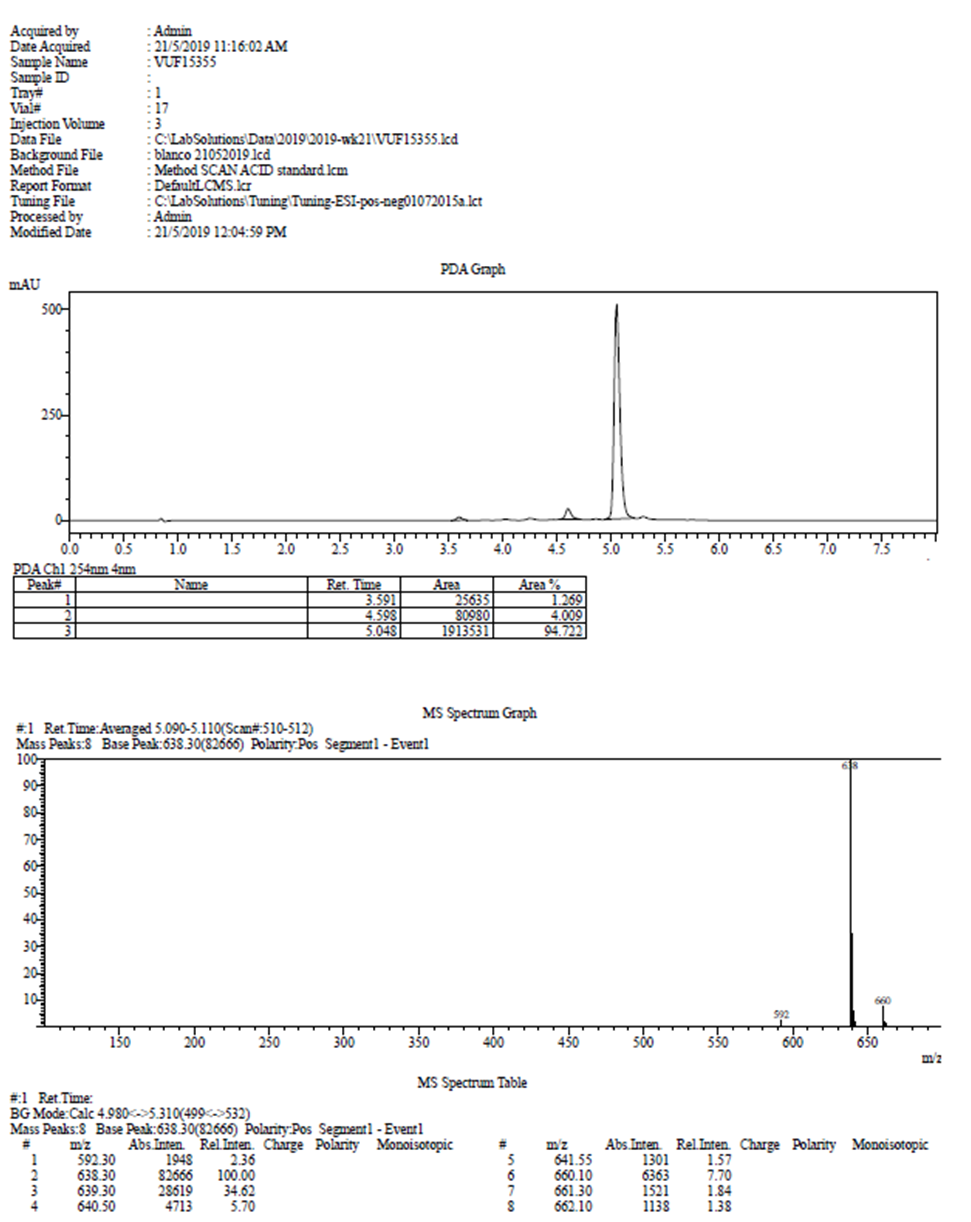


Figure S18. LCMS spectrum of compound **16**

**
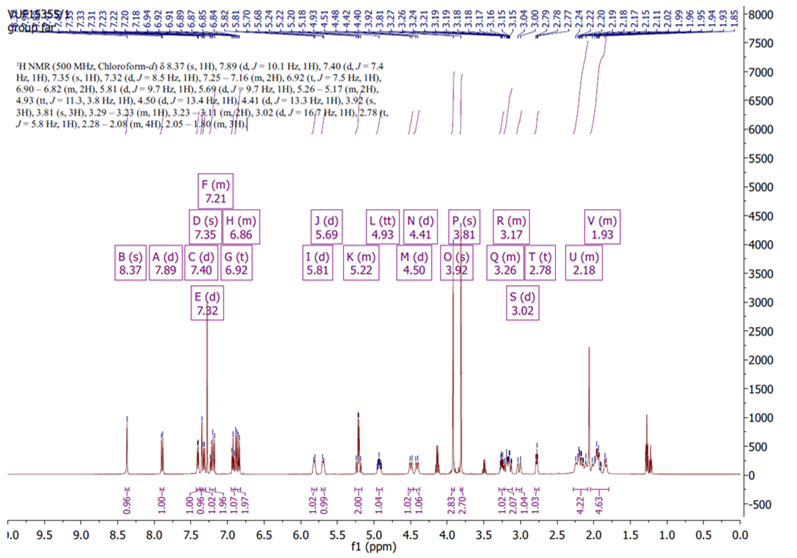
**

Figure S19. ^1^H NMR spectrum of compound **16** in CDCl_3_

**
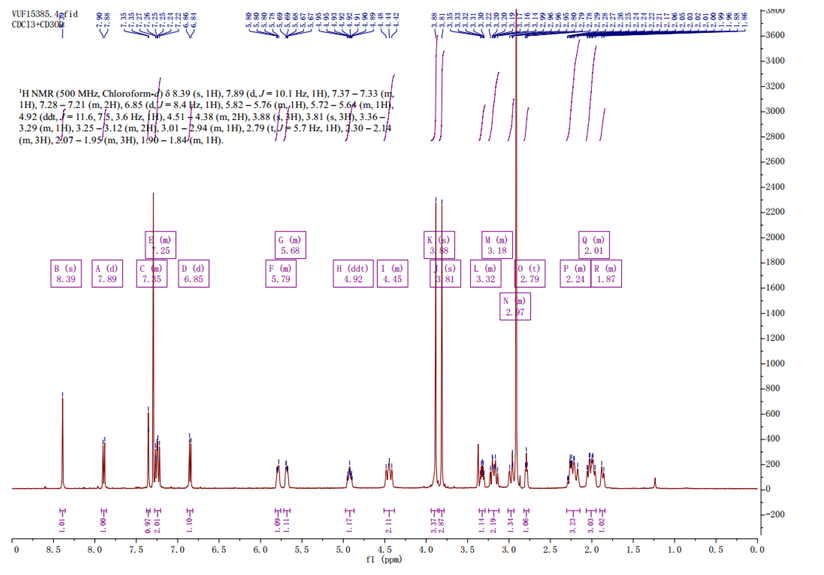
**

Figure S20. ^1^H NMR spectrum of compound **16** in CDCl_3_ + CD_3_OD

**
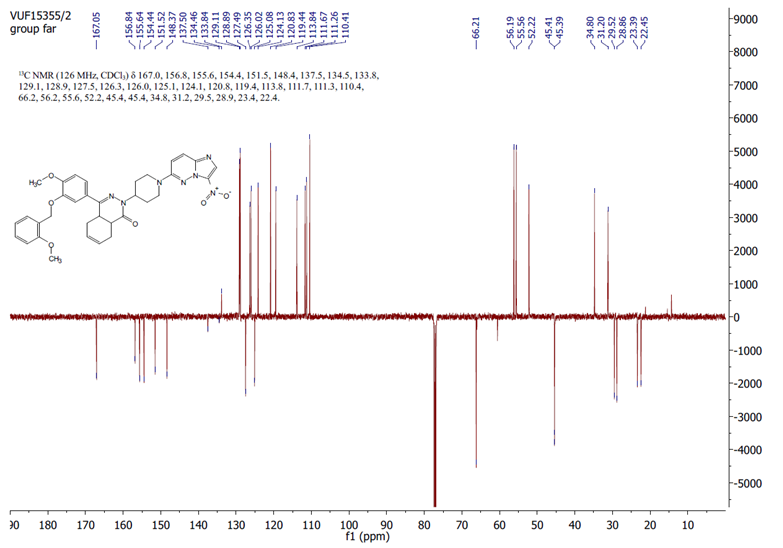
**

Figure S21. ^13^C NMR spectrum of compound **16**


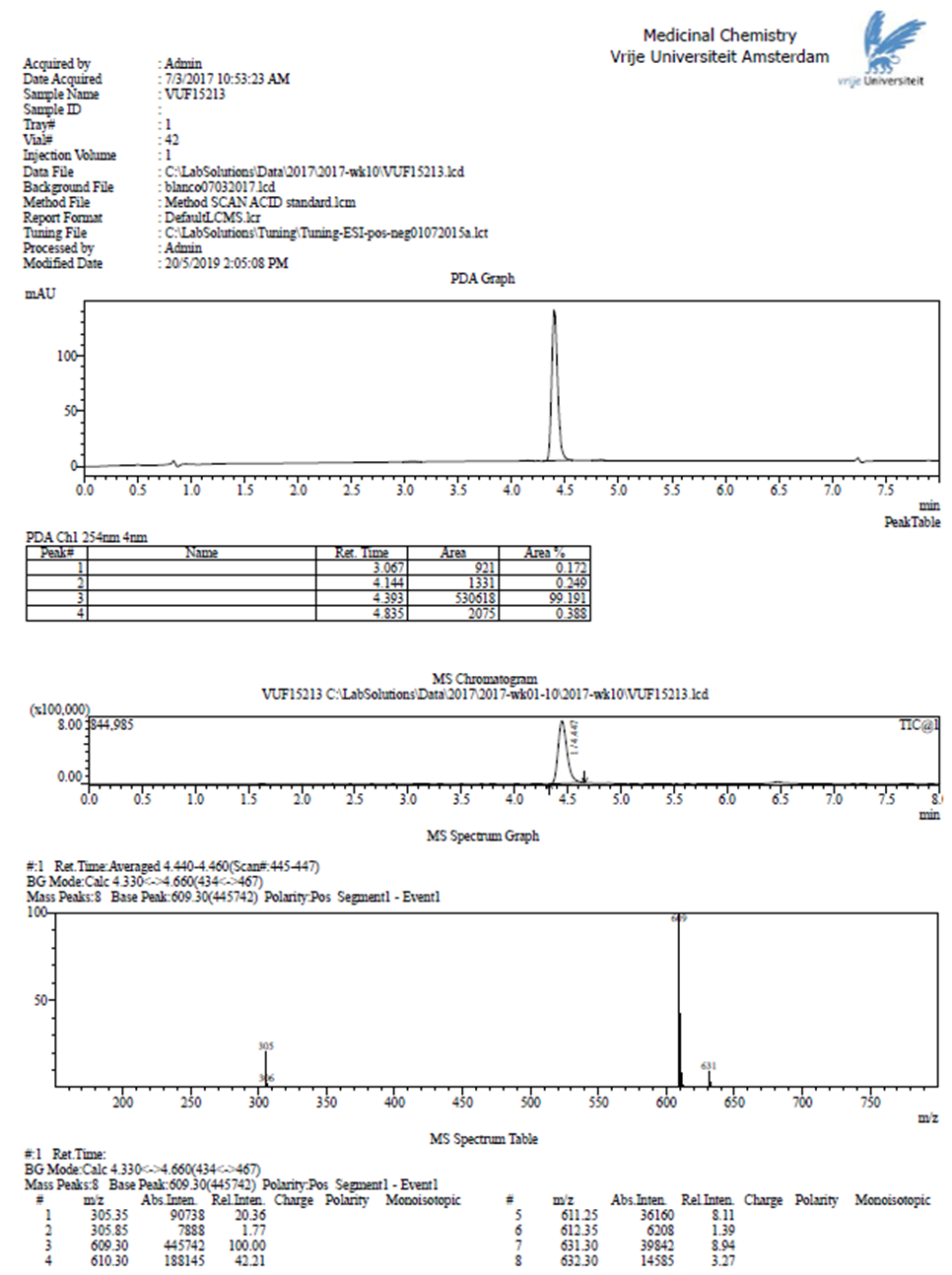


Figure S22. LCMS spectrum of compound **17**


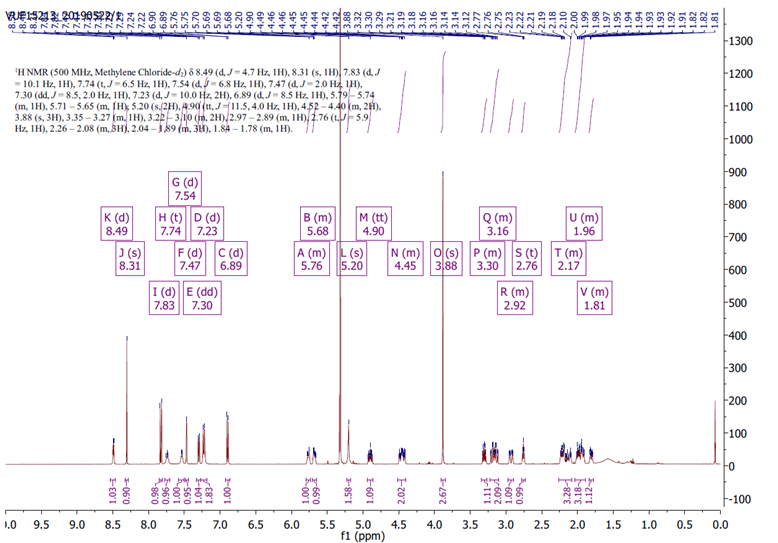


Figure S23. ^1^H NMR spectrum of compound **17**

**
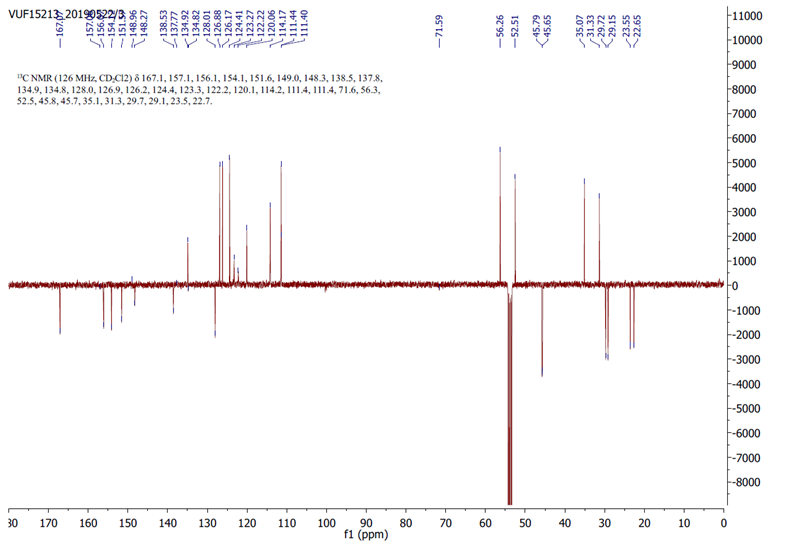
**

Figure S24. ^13^C NMR spectrum of compound **17**

**
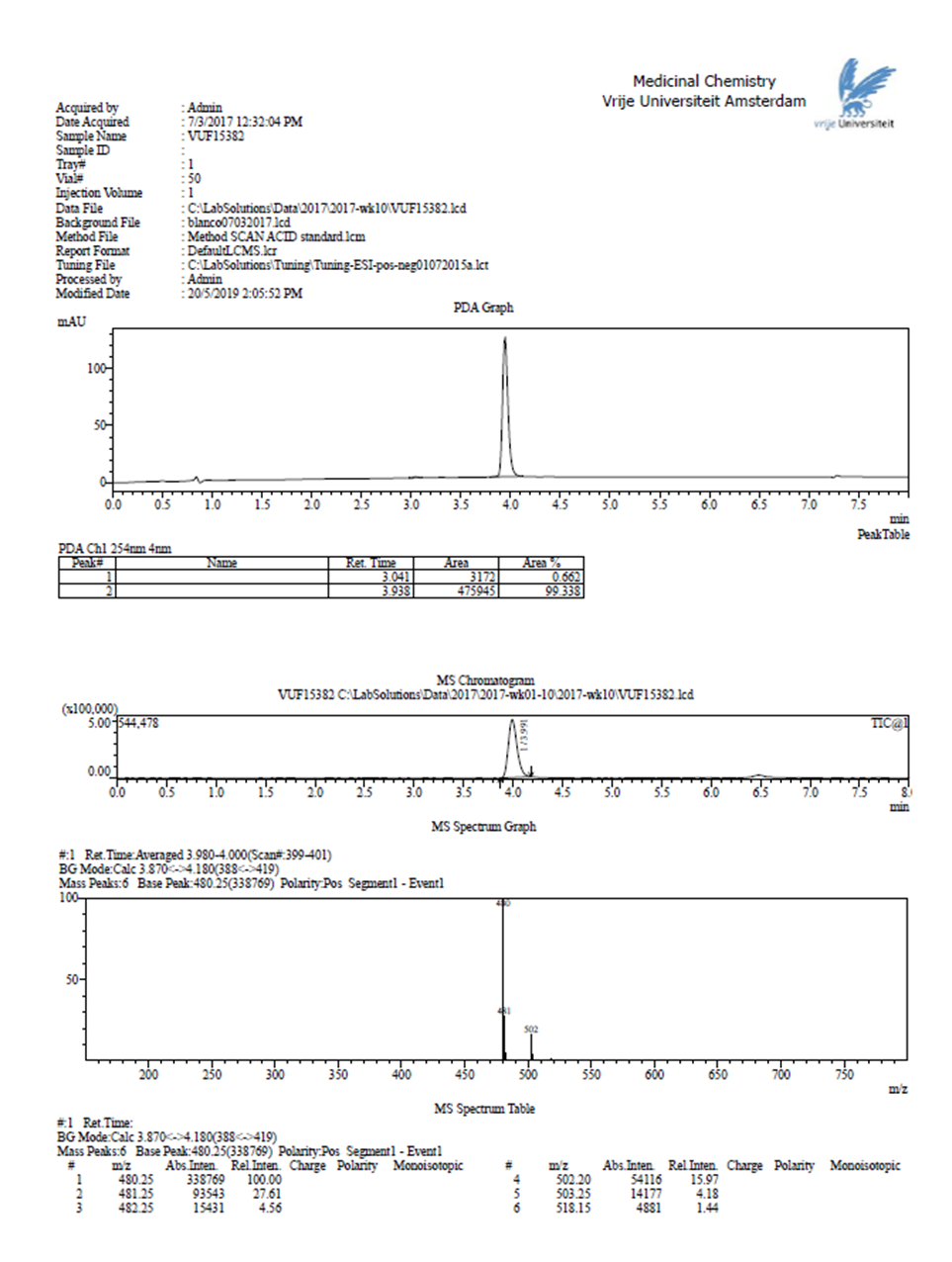
**

Figure S25. LCMS spectrum of compound **18**

**
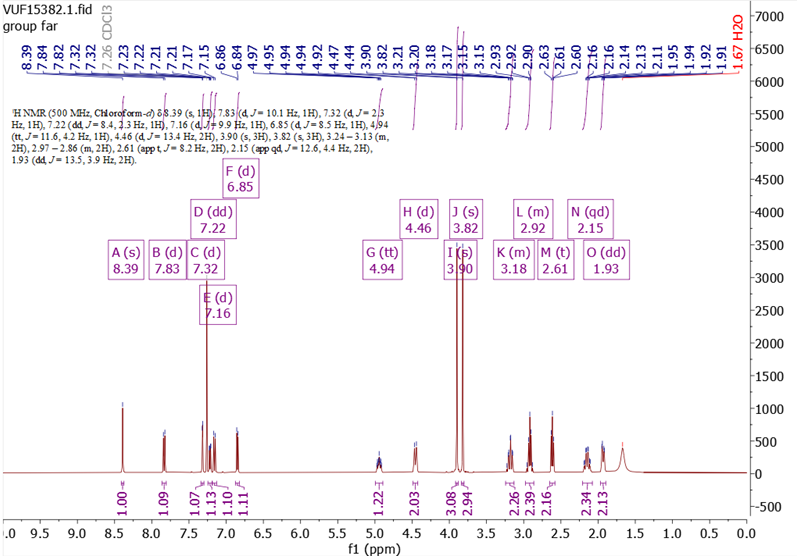
**

Figure S26. ^1^H NMR spectrum of compound **18**

**
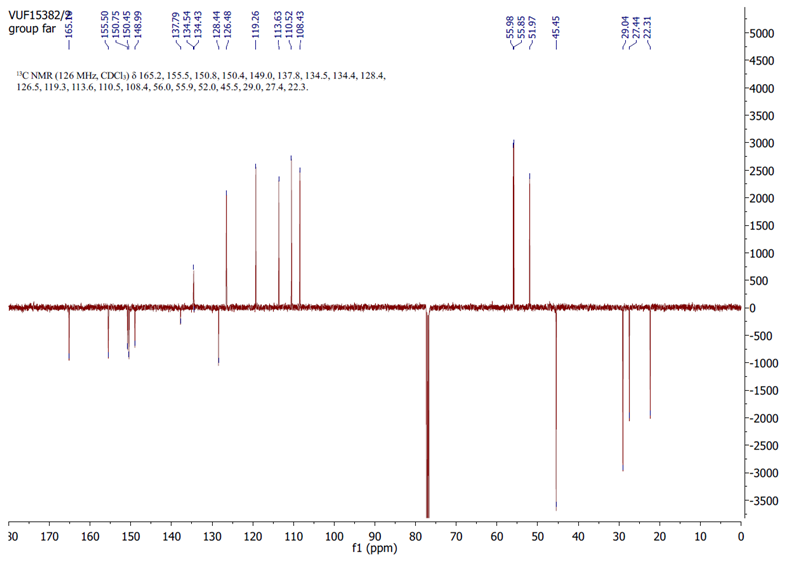
**

Figure S27. ^13^C NMR spectrum of compound **18**


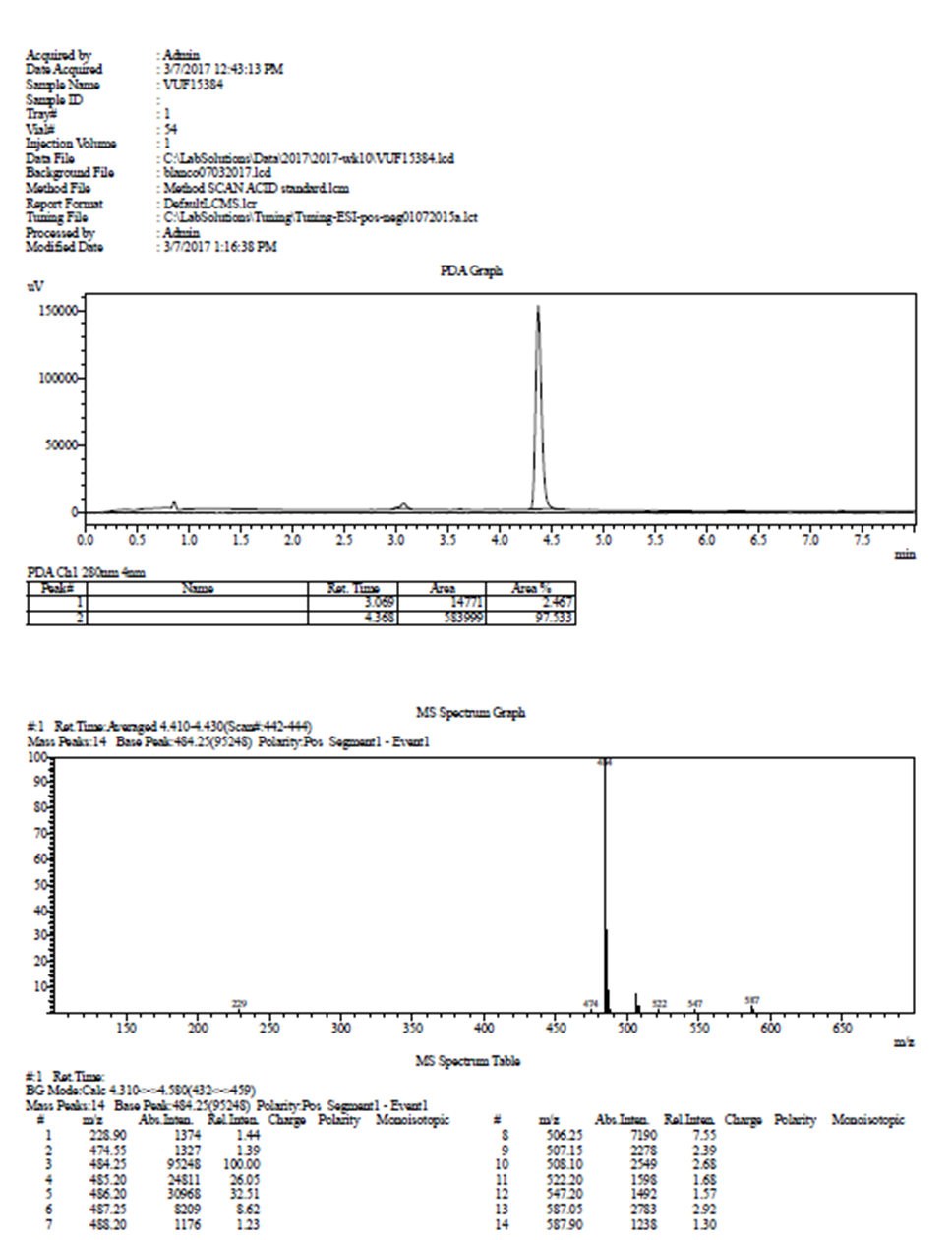


Figure S28. LCMS spectrum of compound **19**


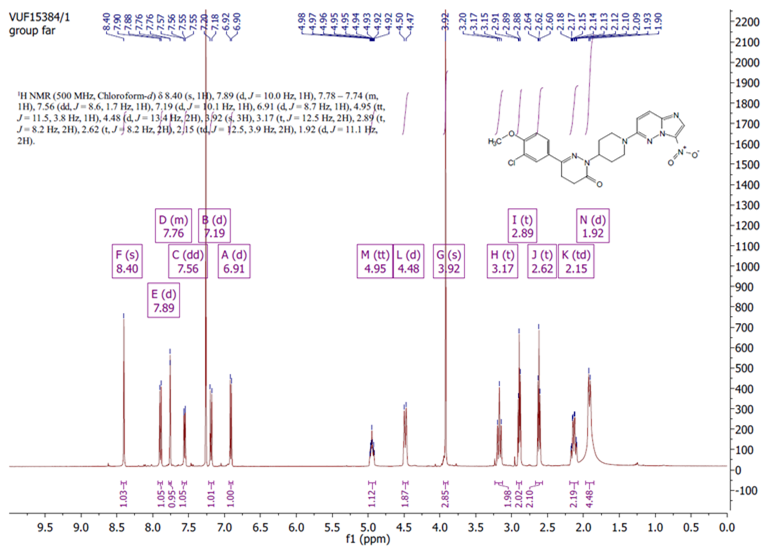


Figure S29. ^1^H NMR spectrum of compound **19**

**
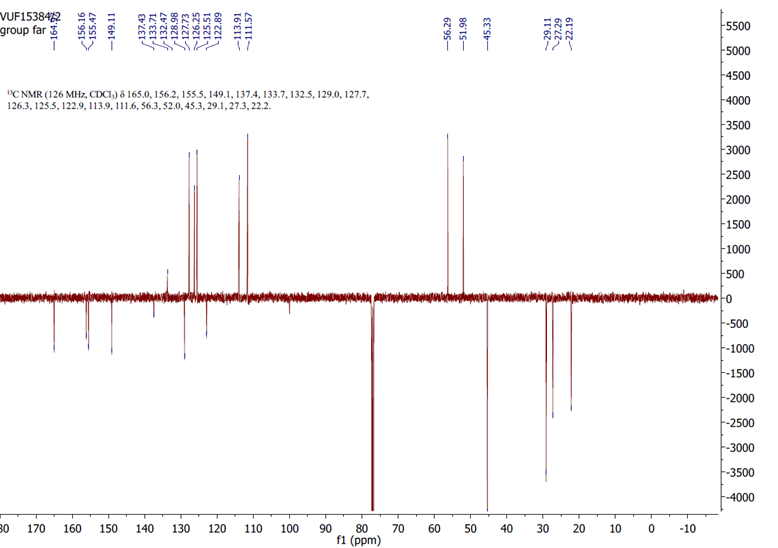
**

Figure S30. ^13^C NMR spectrum of compound **19**


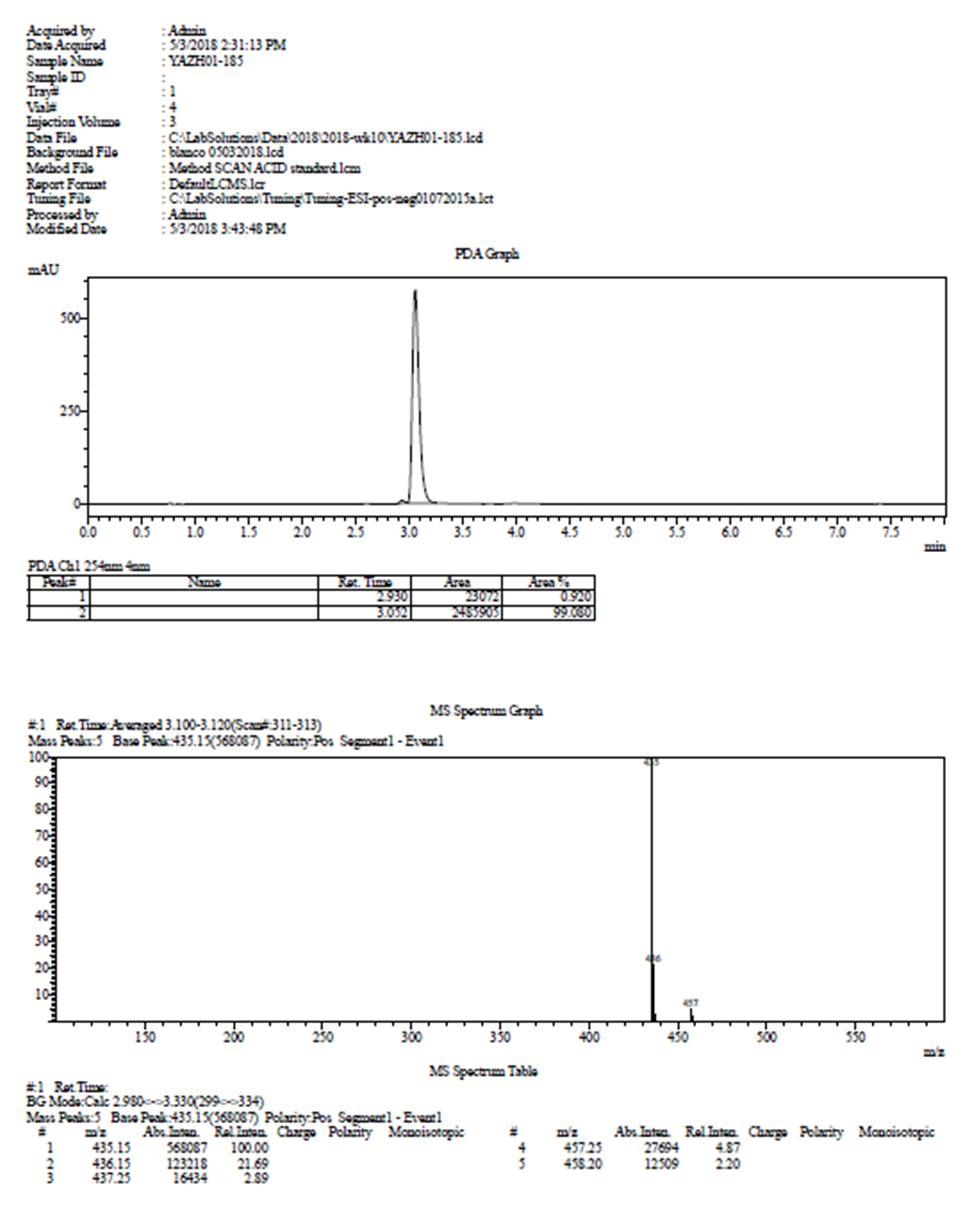


Figure S31. LCMS spectrum of compound **20**


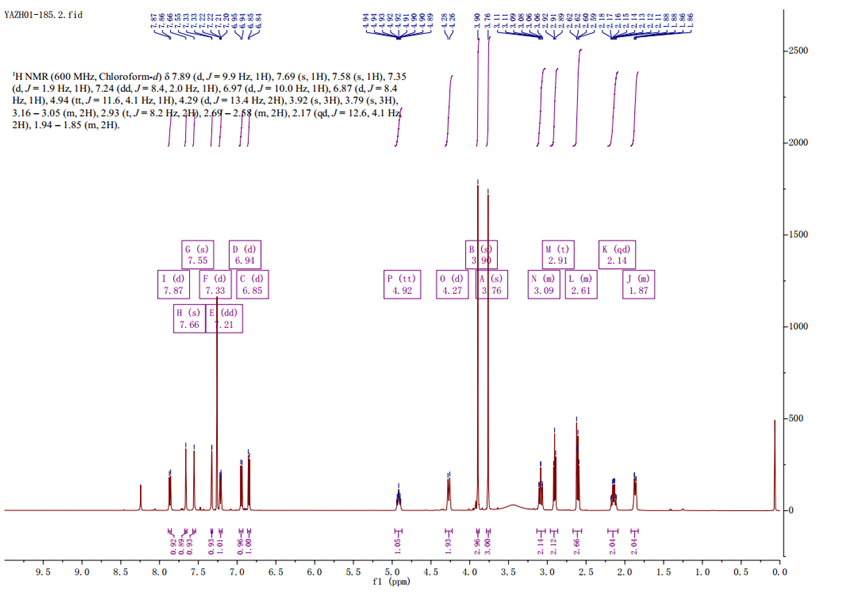


Figure S32. ^1^H NMR spectrum of compound **20**

**
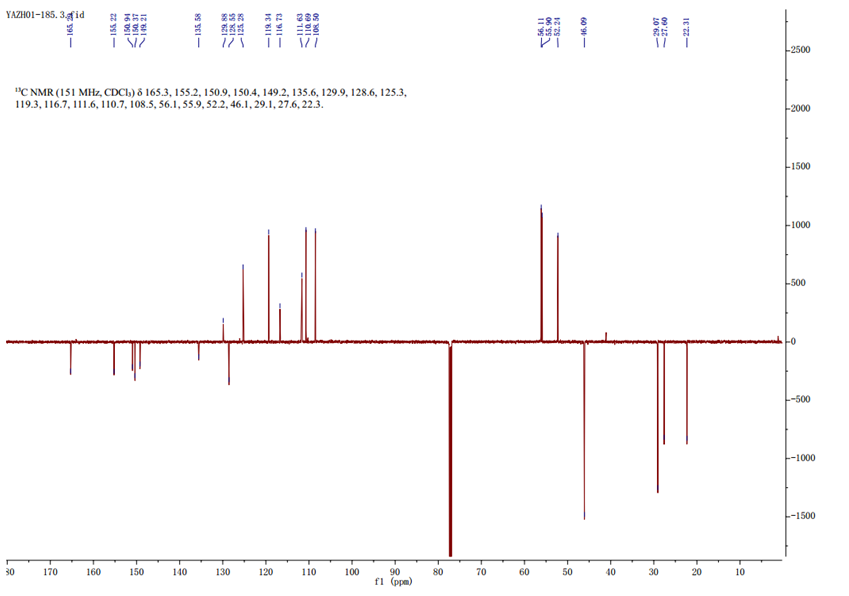
**

Figure S33. ^13^C NMR spectrum of compound **20**


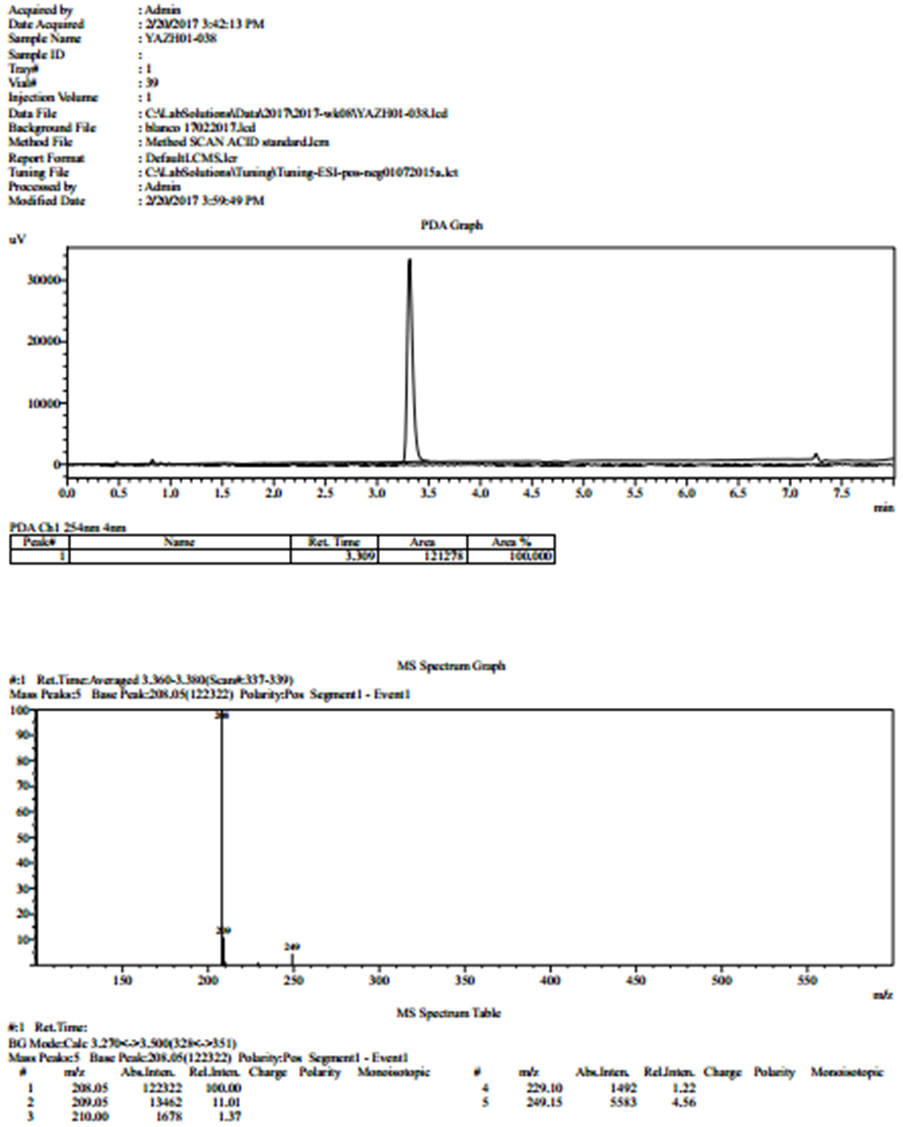


Figure S34. LCMS spectrum of compound **21**


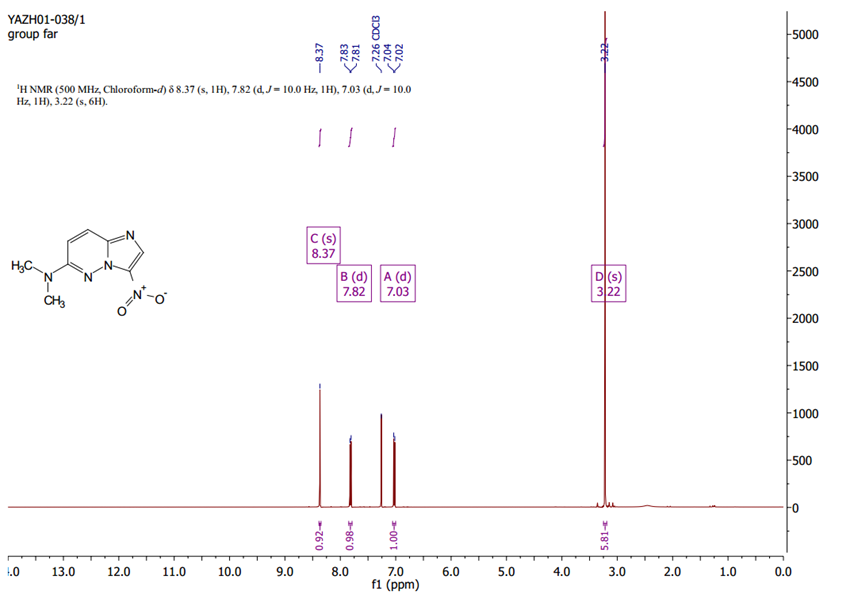


Figure S35. ^1^H NMR spectrum of compound **21**

**
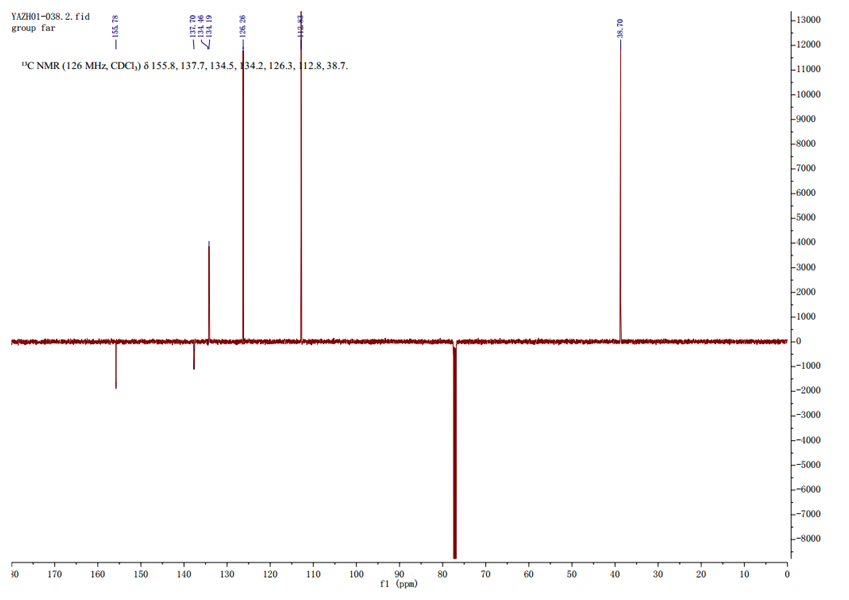
**

Figure S36. ^13^C NMR spectrum of compound **21**


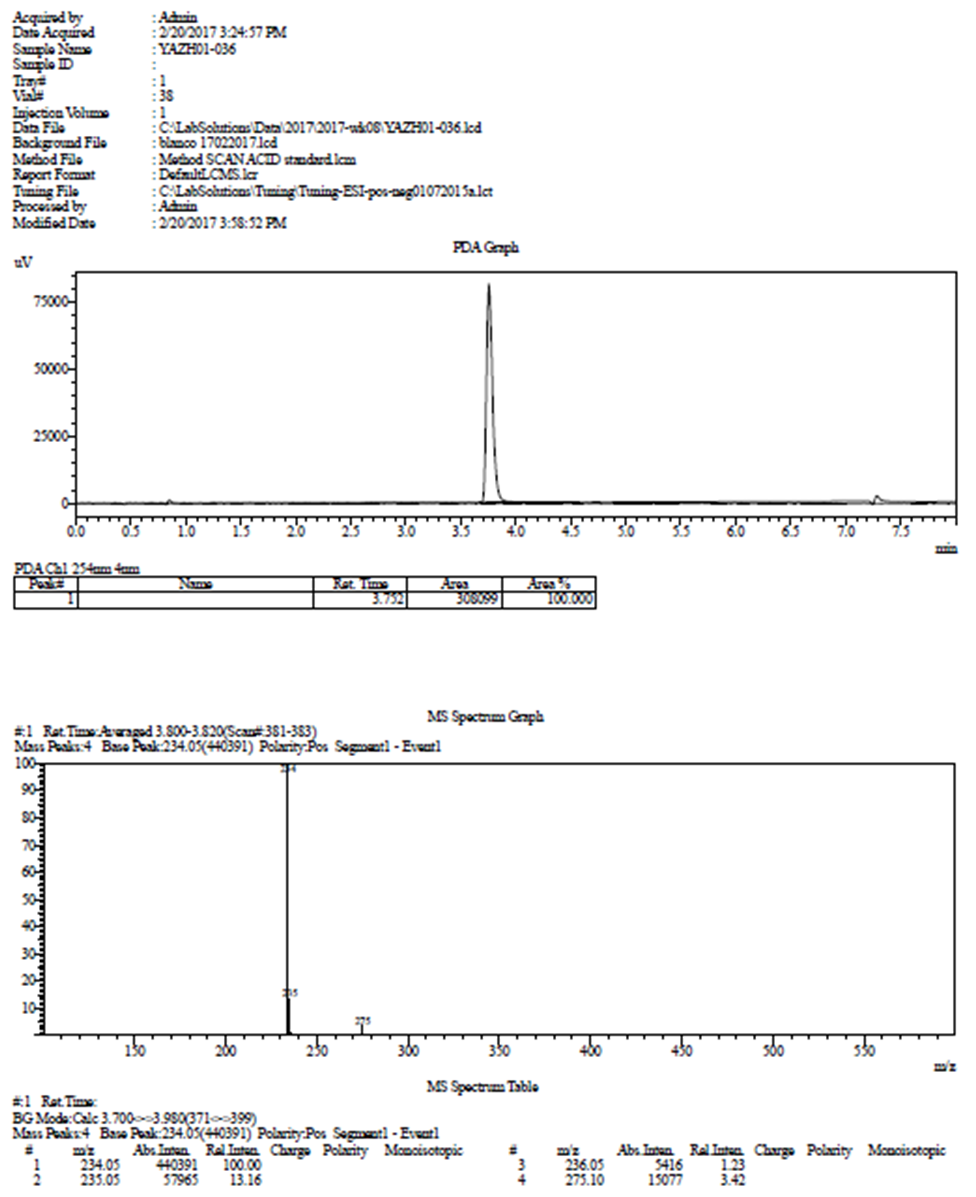


Figure S37. LCMS spectrum of compound **22**


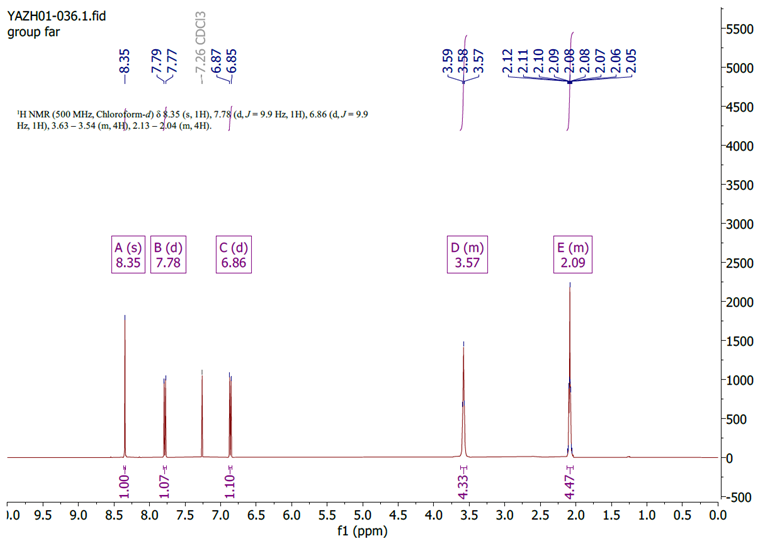


Figure S38. ^1^H NMR spectrum of compound **22**

**
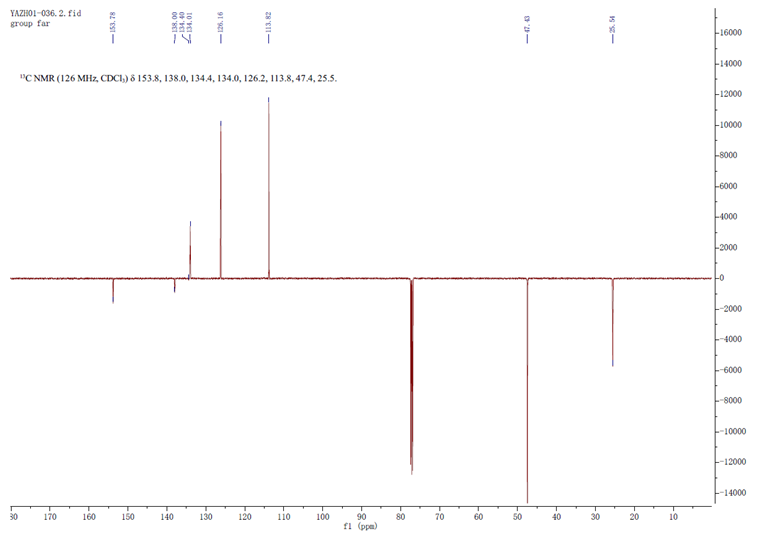
**

Figure S39. ^13^C NMR spectrum of compound **22**


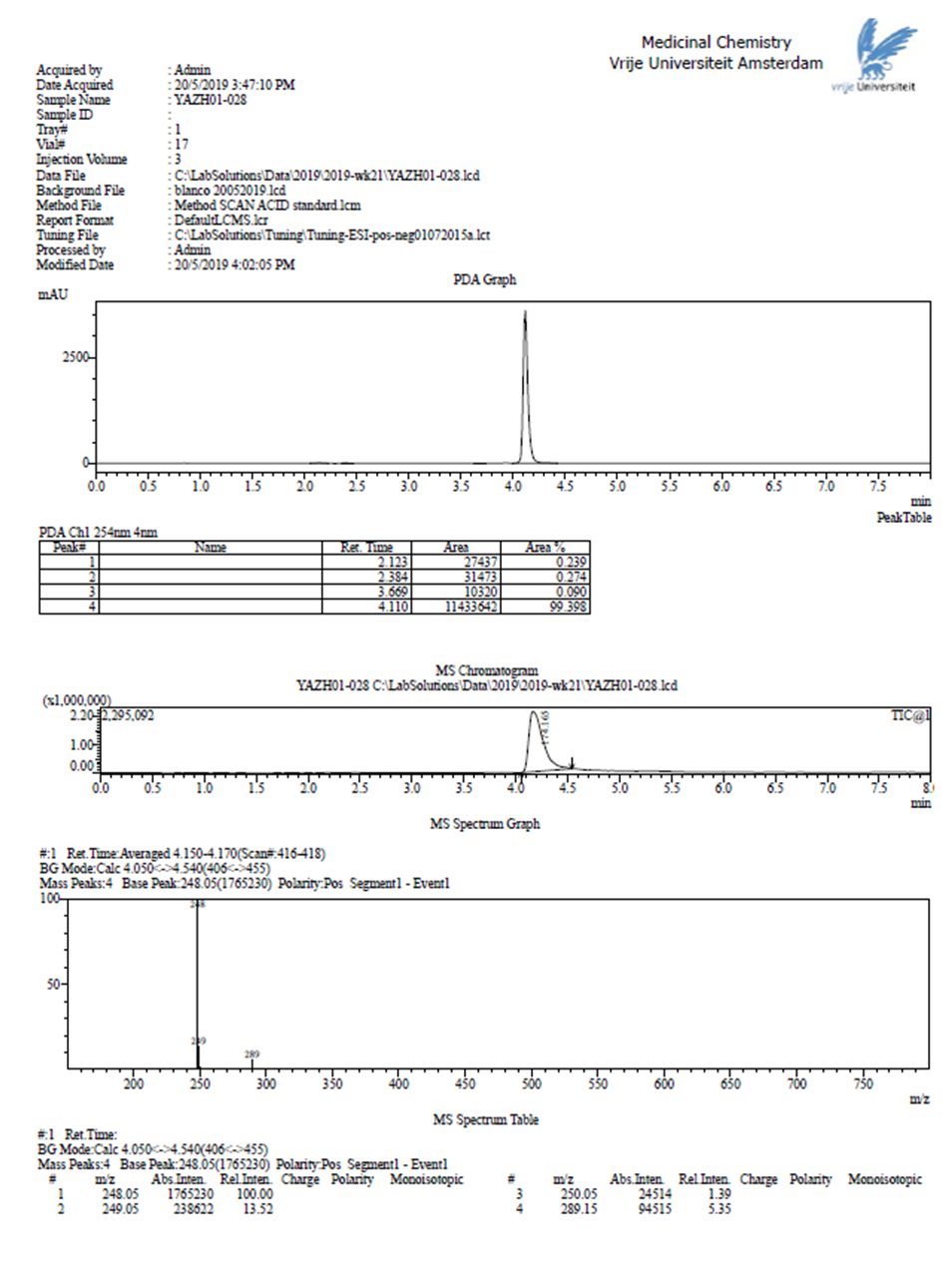


Figure S40. LCMS spectrum of compound **23**


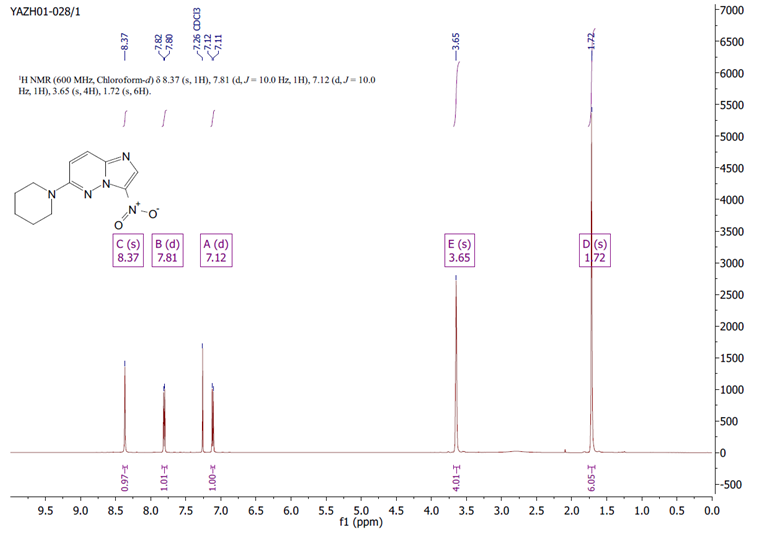


Figure S41. ^1^H NMR spectrum of compound **23**

**
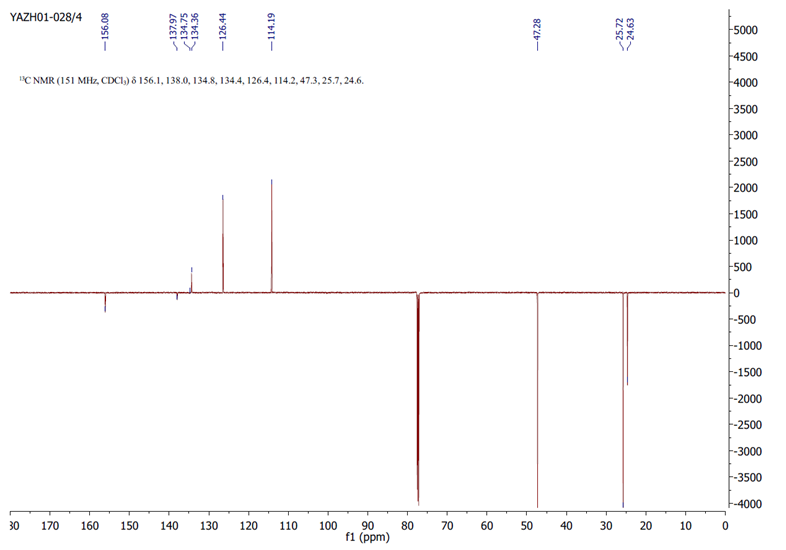
**

Figure S42. ^13^C NMR spectrum of compound **23**


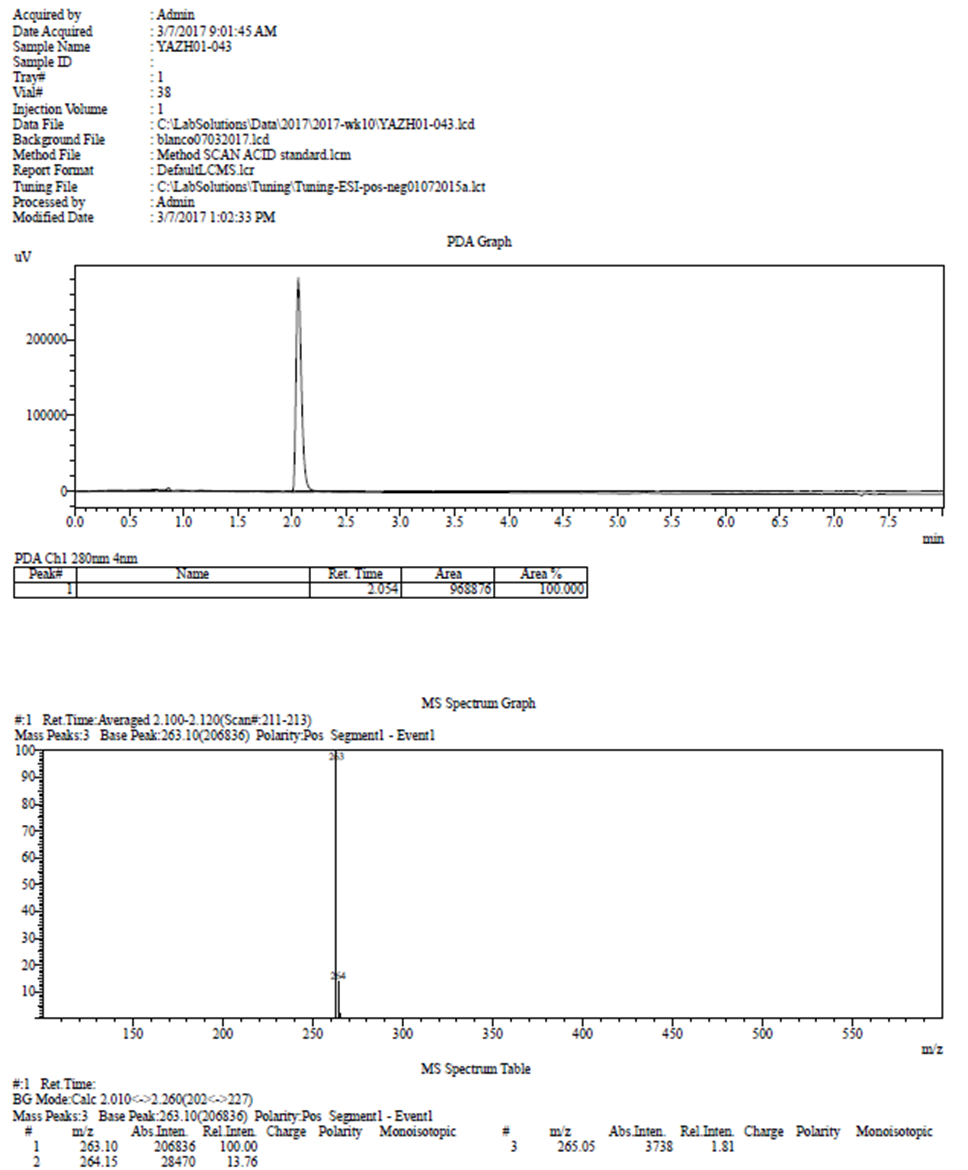


Figure S43. LCMS spectrum of compound **24**


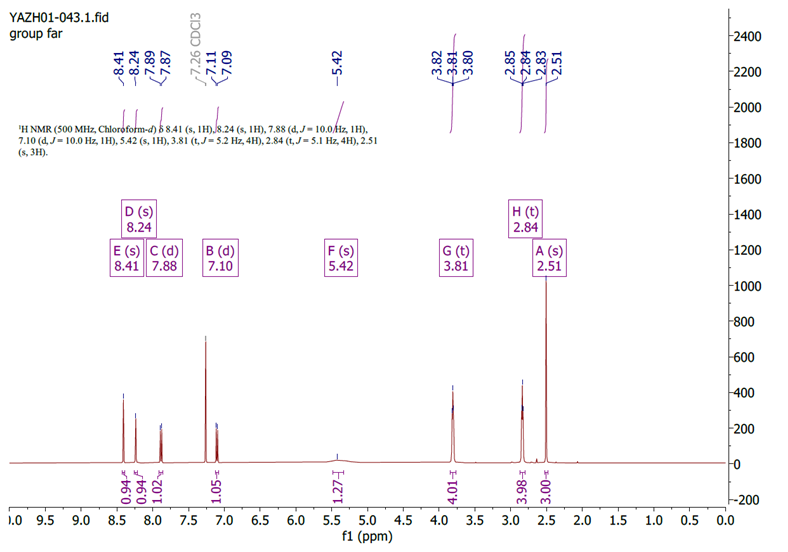


Figure S44. ^1^H NMR spectrum of compound **24**

**
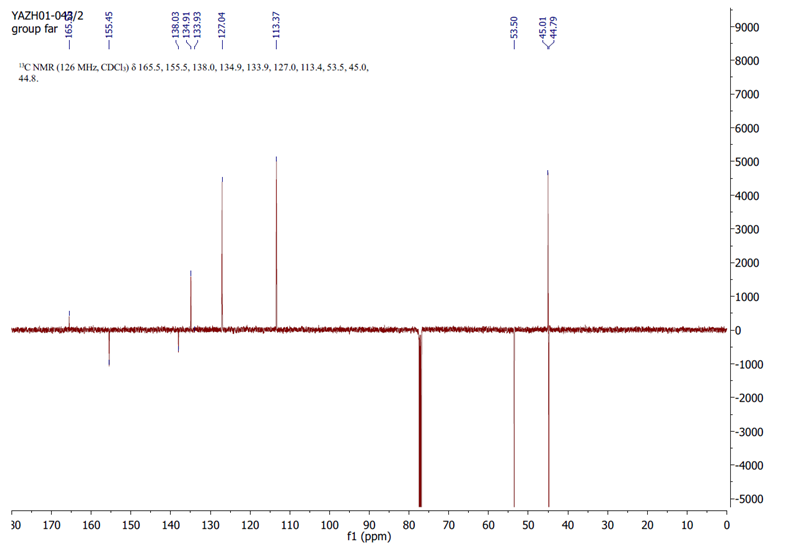
**

Figure S45. ^13^C NMR spectrum of compound **24**


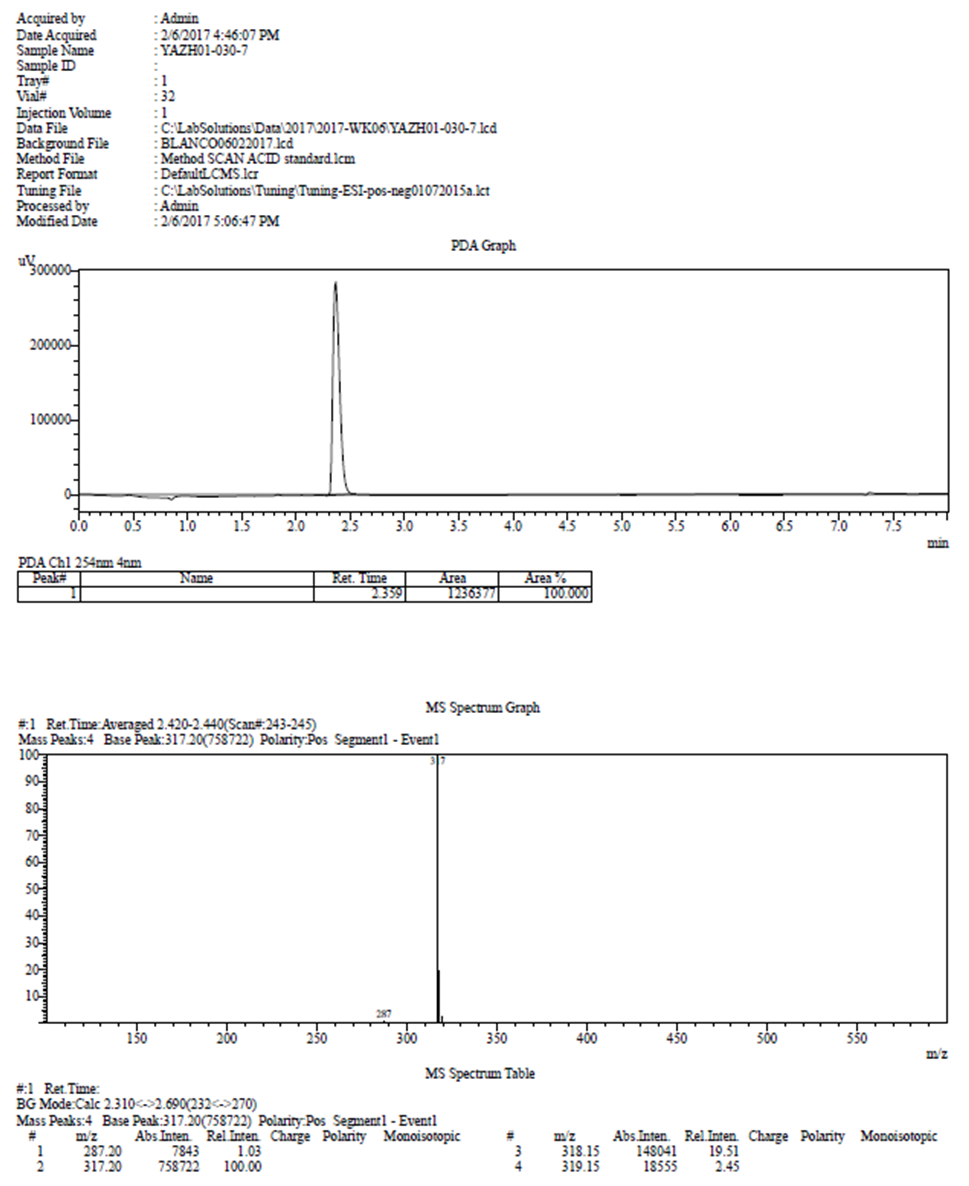


Figure S46. LCMS spectrum of compound **25**


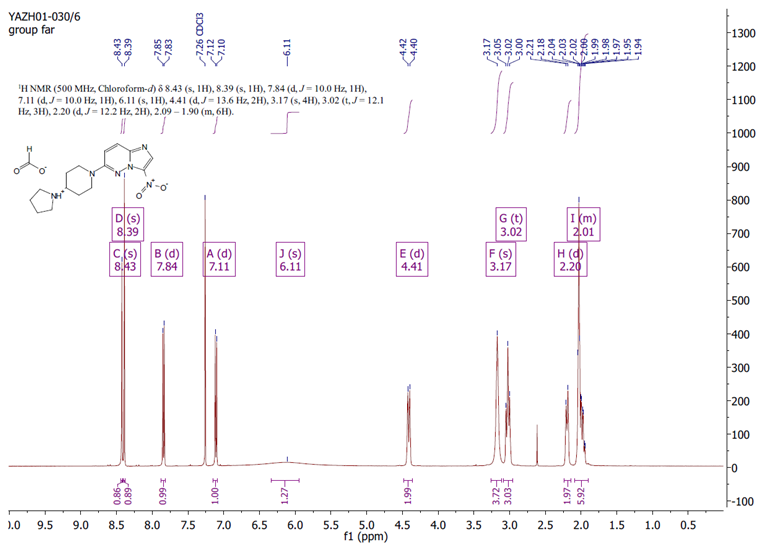


Figure S47. ^1^H NMR spectrum of compound **25**

**
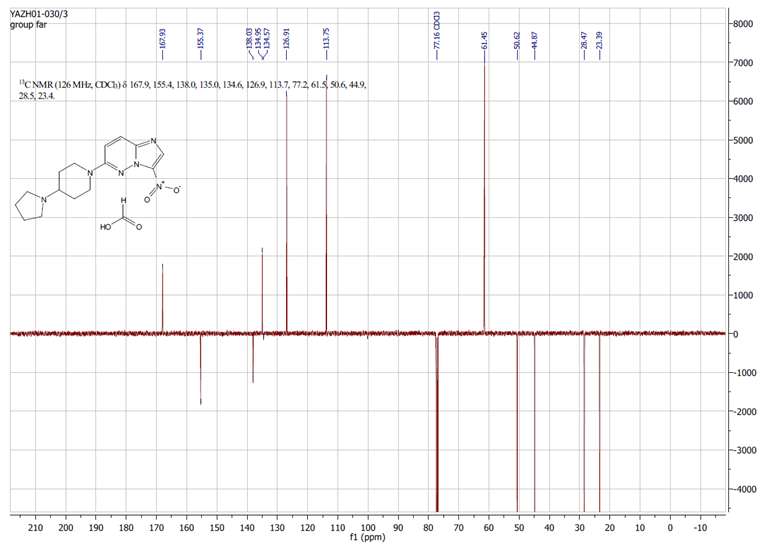
**

Figure S48. ^13^C NMR spectrum of compound **25**


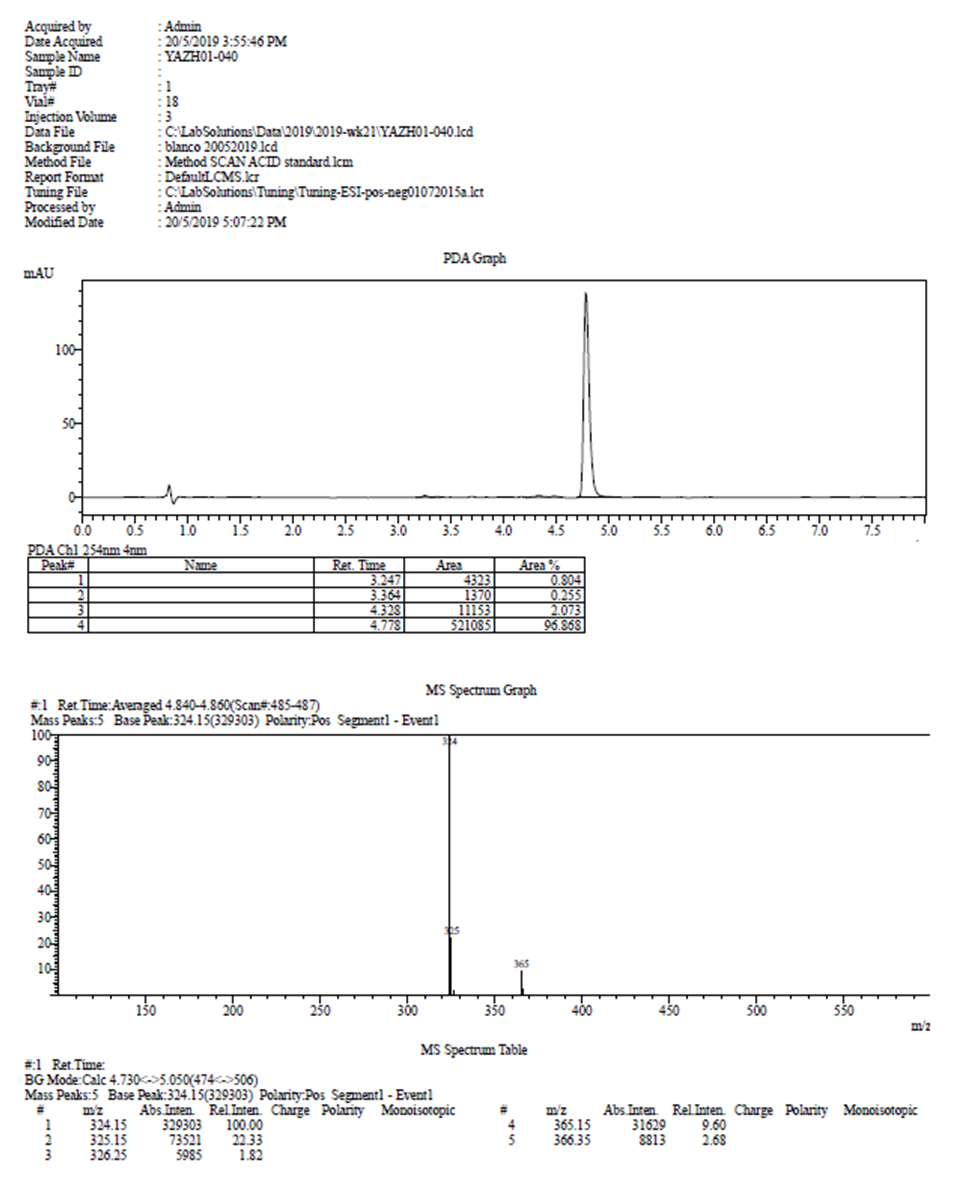


Figure S49. LCMS spectrum of compound **26**


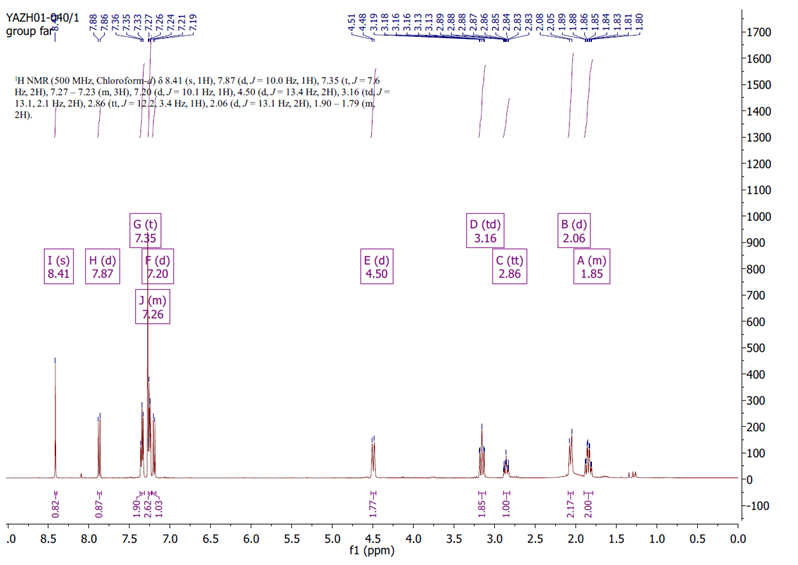


Figure S50. ^1^H NMR spectrum of compound **26**

**
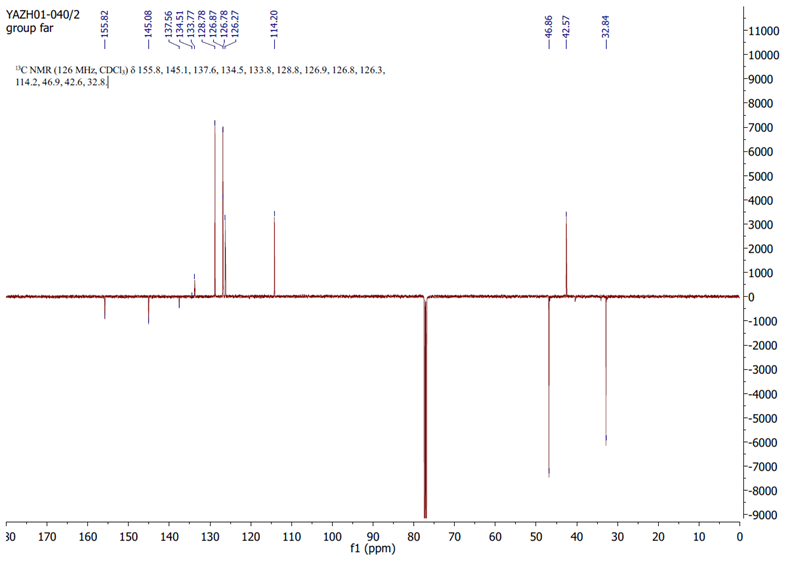
**

Figure S51. ^13^C NMR spectrum of compound **26**


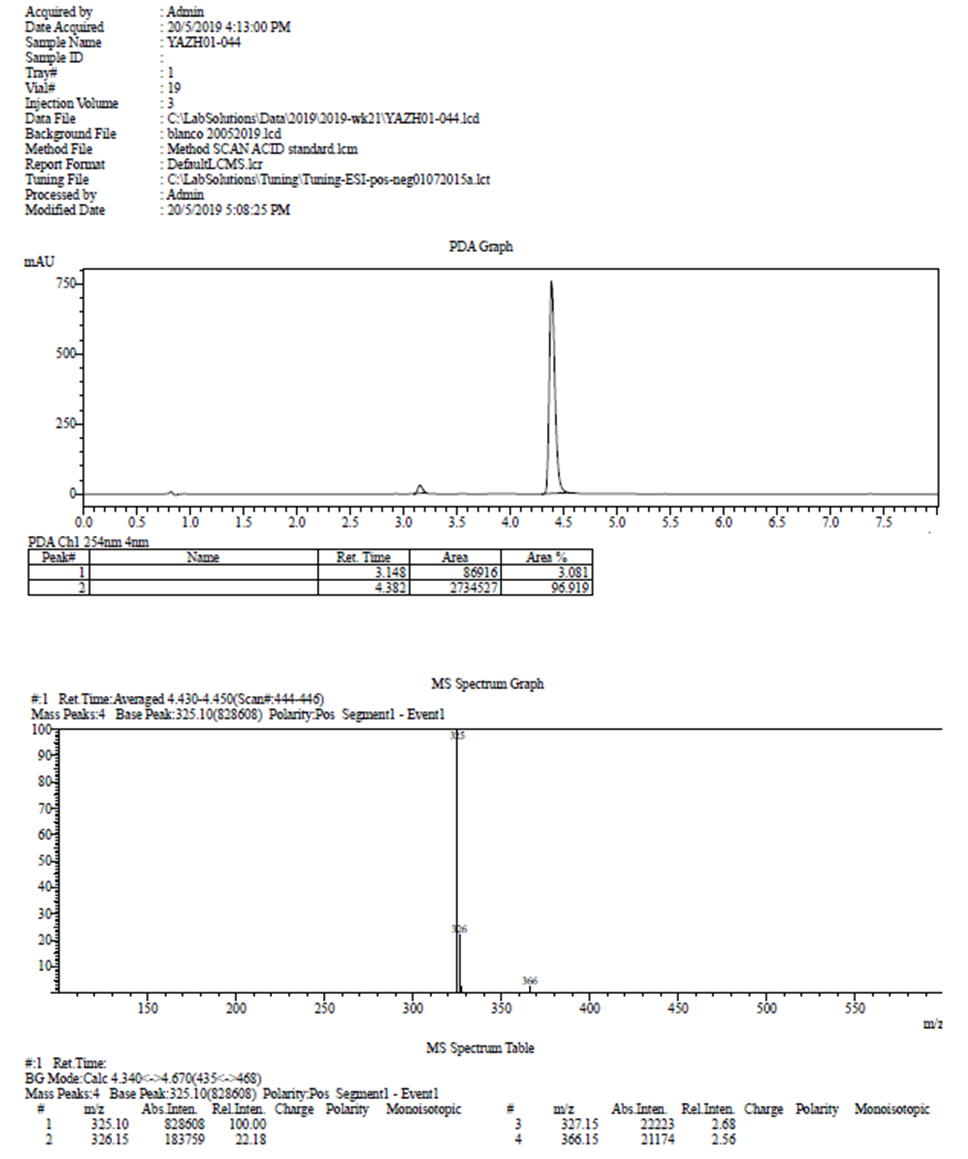


Figure S52. LCMS spectrum of compound **27**


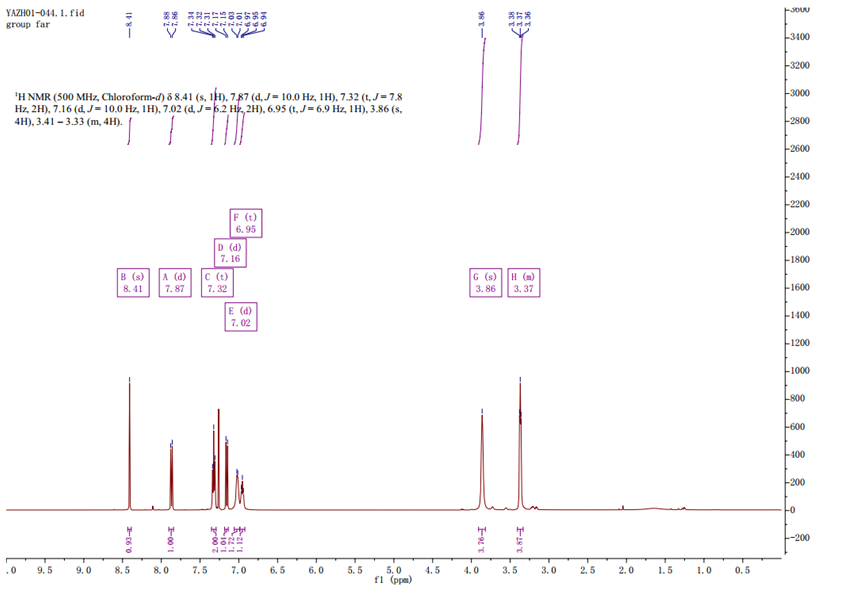


Figure S53. ^1^H NMR spectrum of compound **27**

**
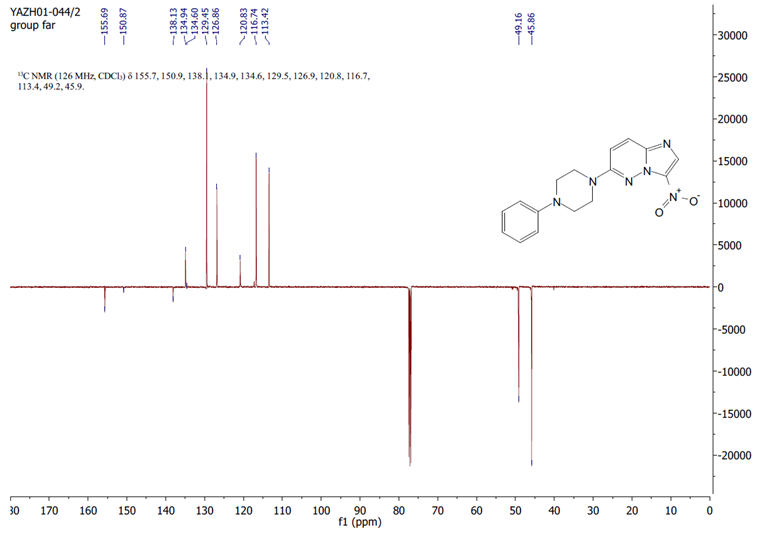
**

Figure S54. ^13^C NMR spectrum of compound **27**

**
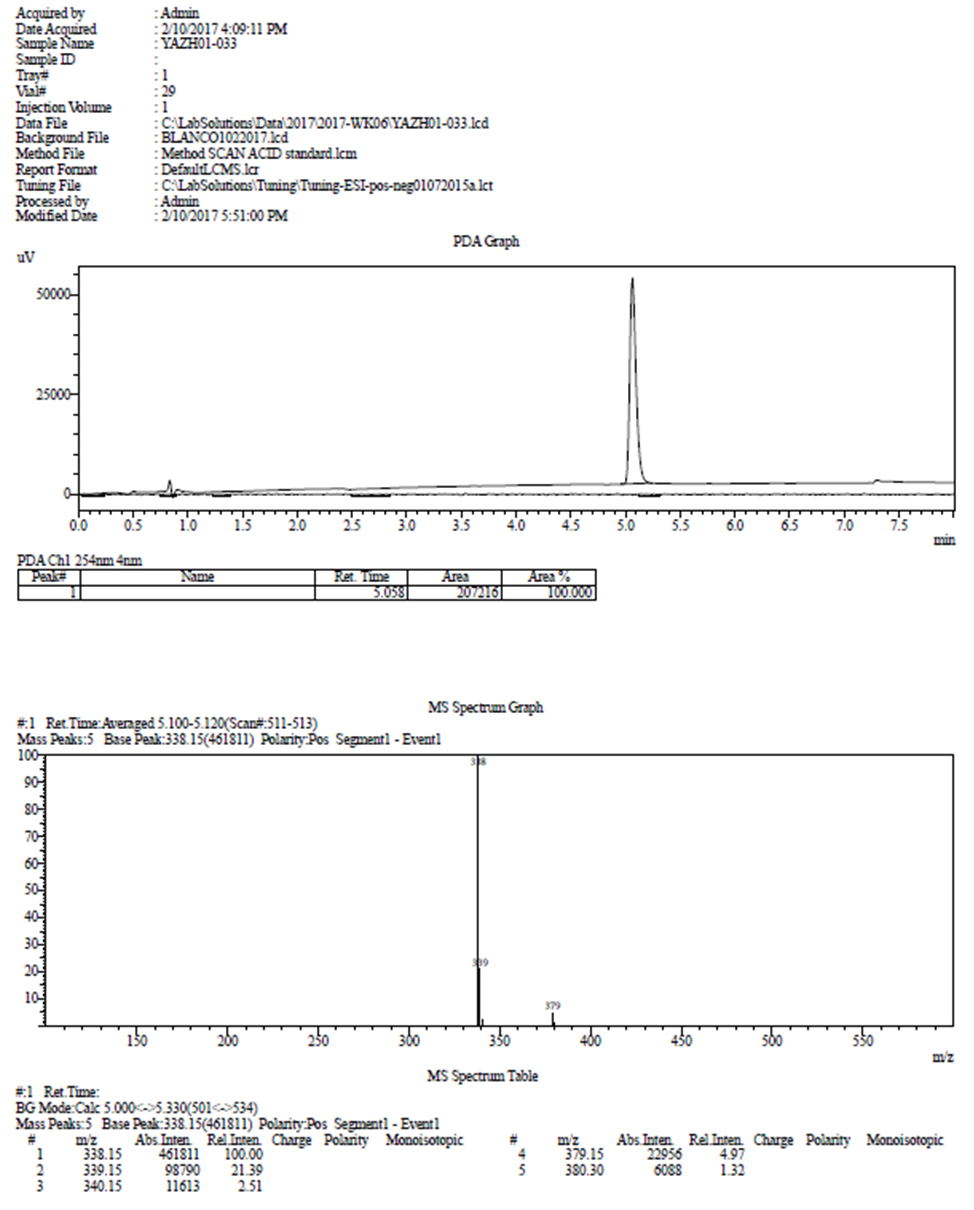
**

Figure S55. LCMS spectrum of compound **28**


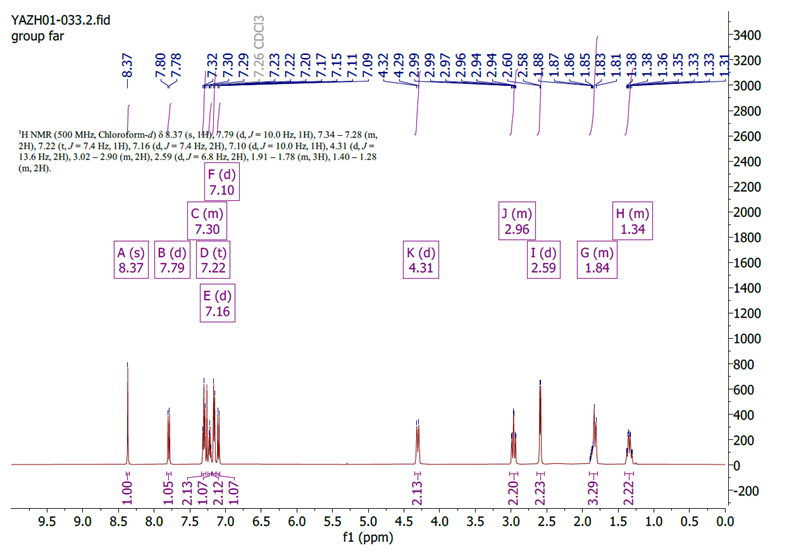


Figure S56. ^1^H NMR spectrum of compound **28**

**
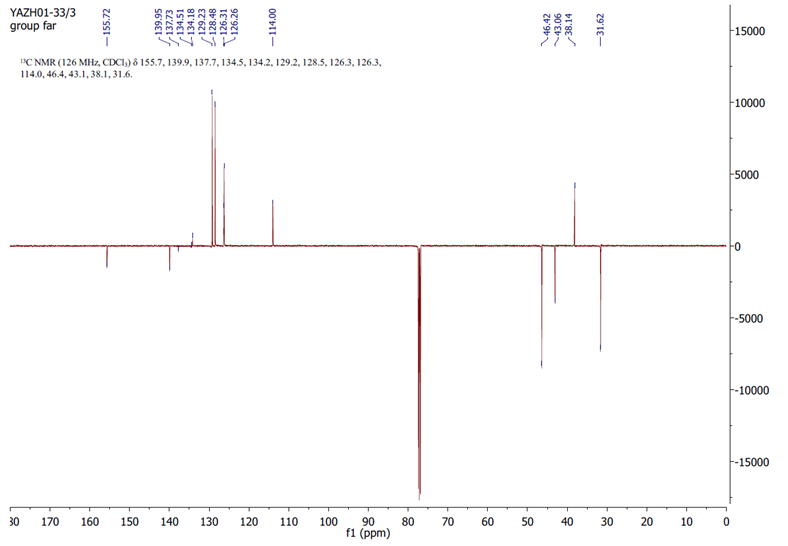
**

Figure S57. ^13^C NMR spectrum of compound **28**


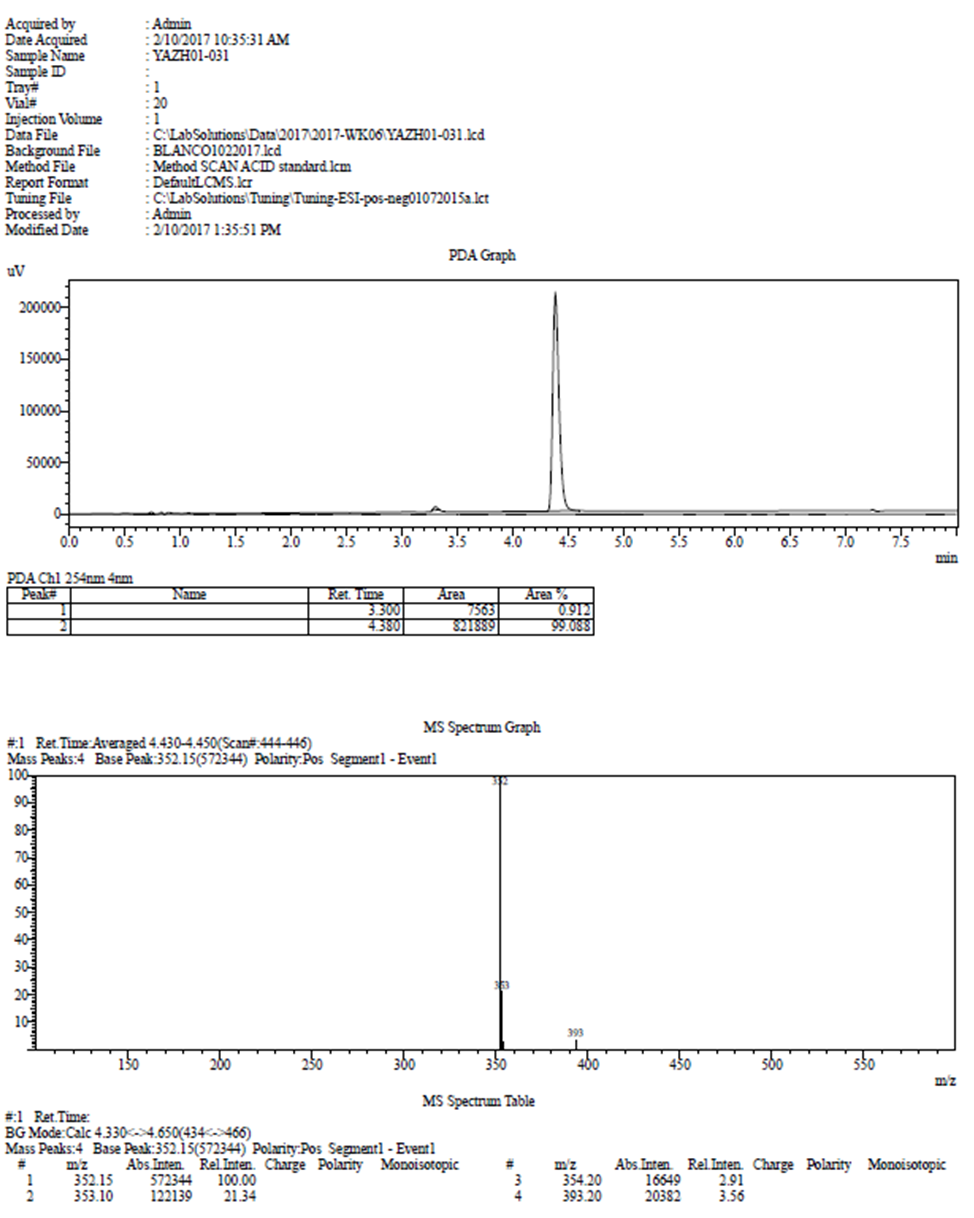


Figure S58. LCMS spectrum of compound **29**


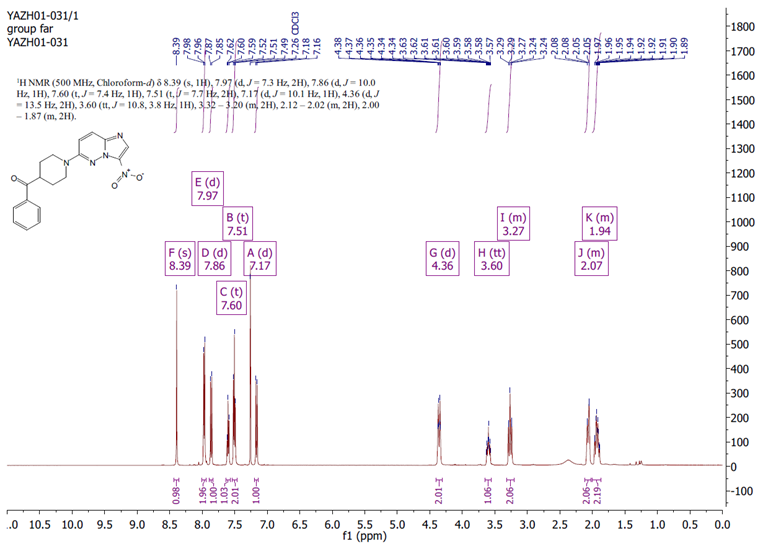


Figure S59. ^1^H NMR spectrum of compound **29**

**
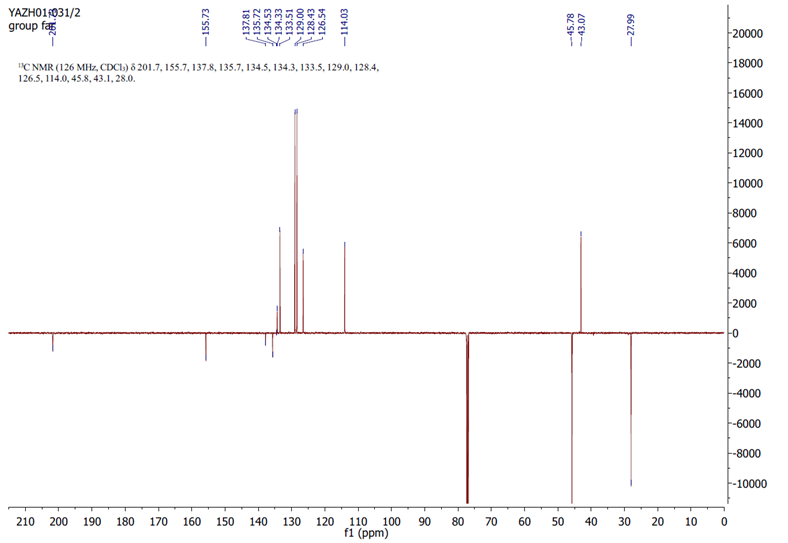
**

Figure S60. ^13^C NMR spectrum of compound **29**

**
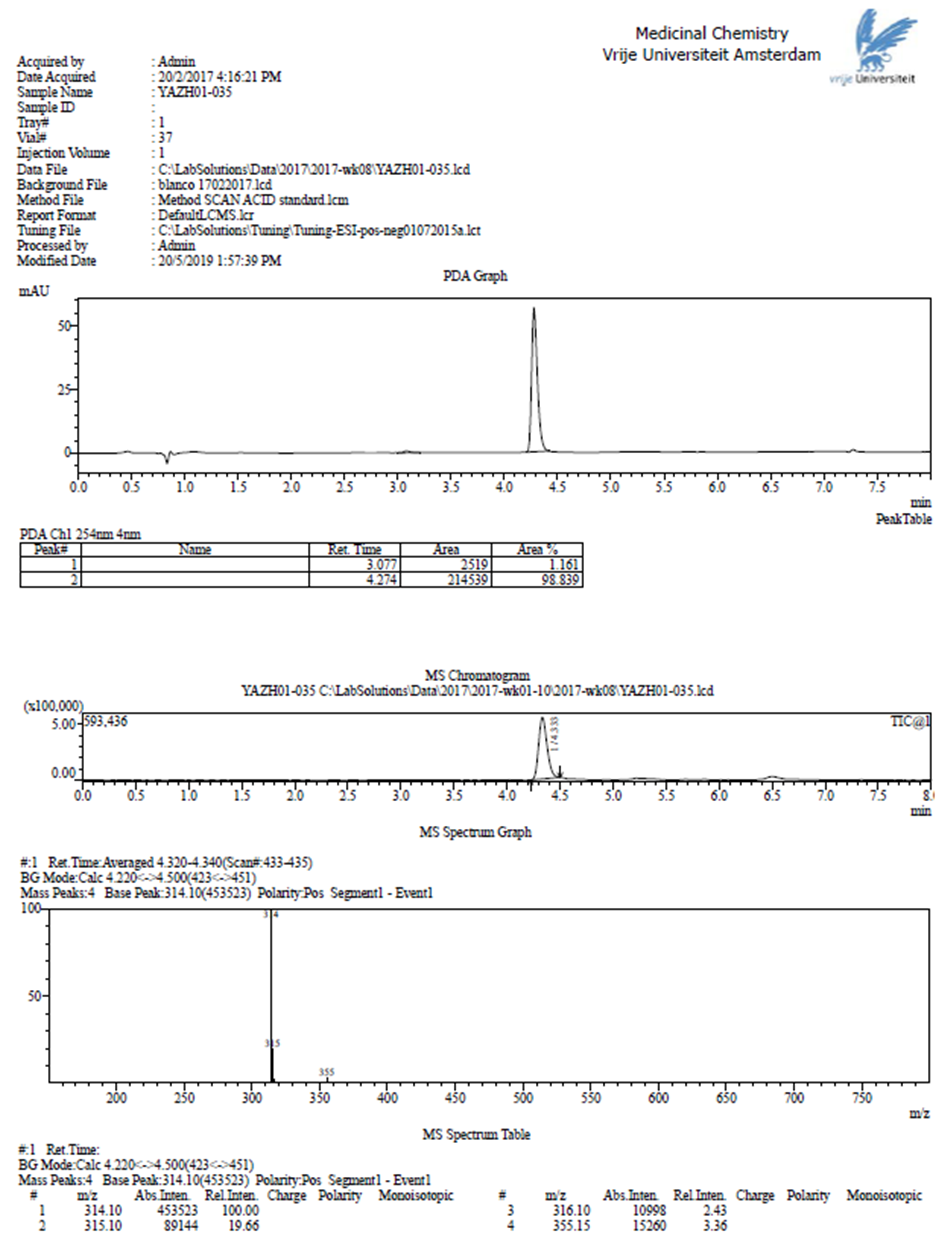
**

Figure S61. LCMS spectrum of compound **30**

**
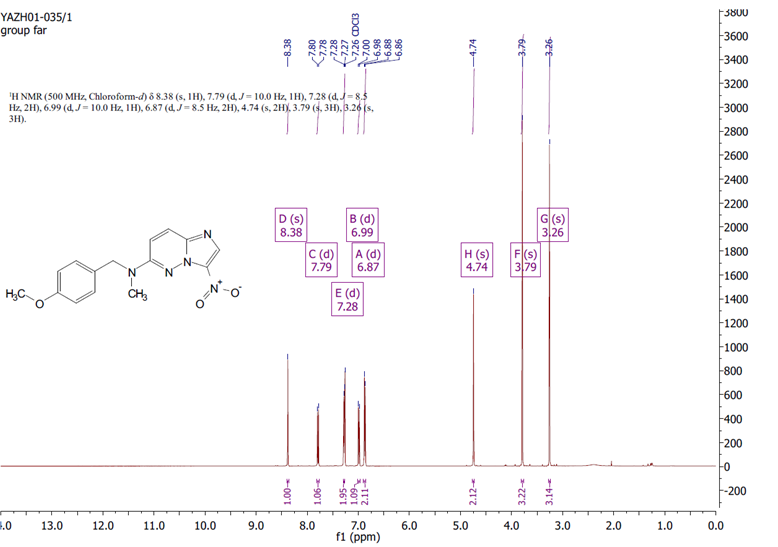
**

Figure S62. ^1^H NMR spectrum of compound **30**

**
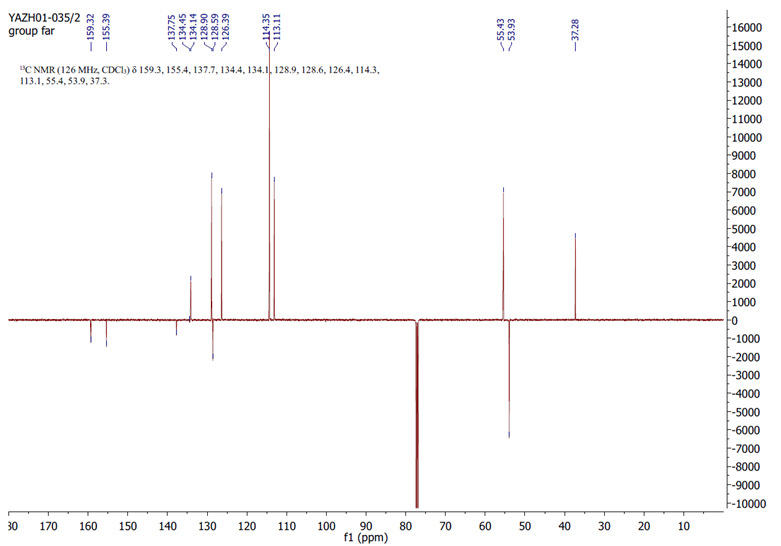
**

Figure S63. ^13^C NMR spectrum of compound **30**


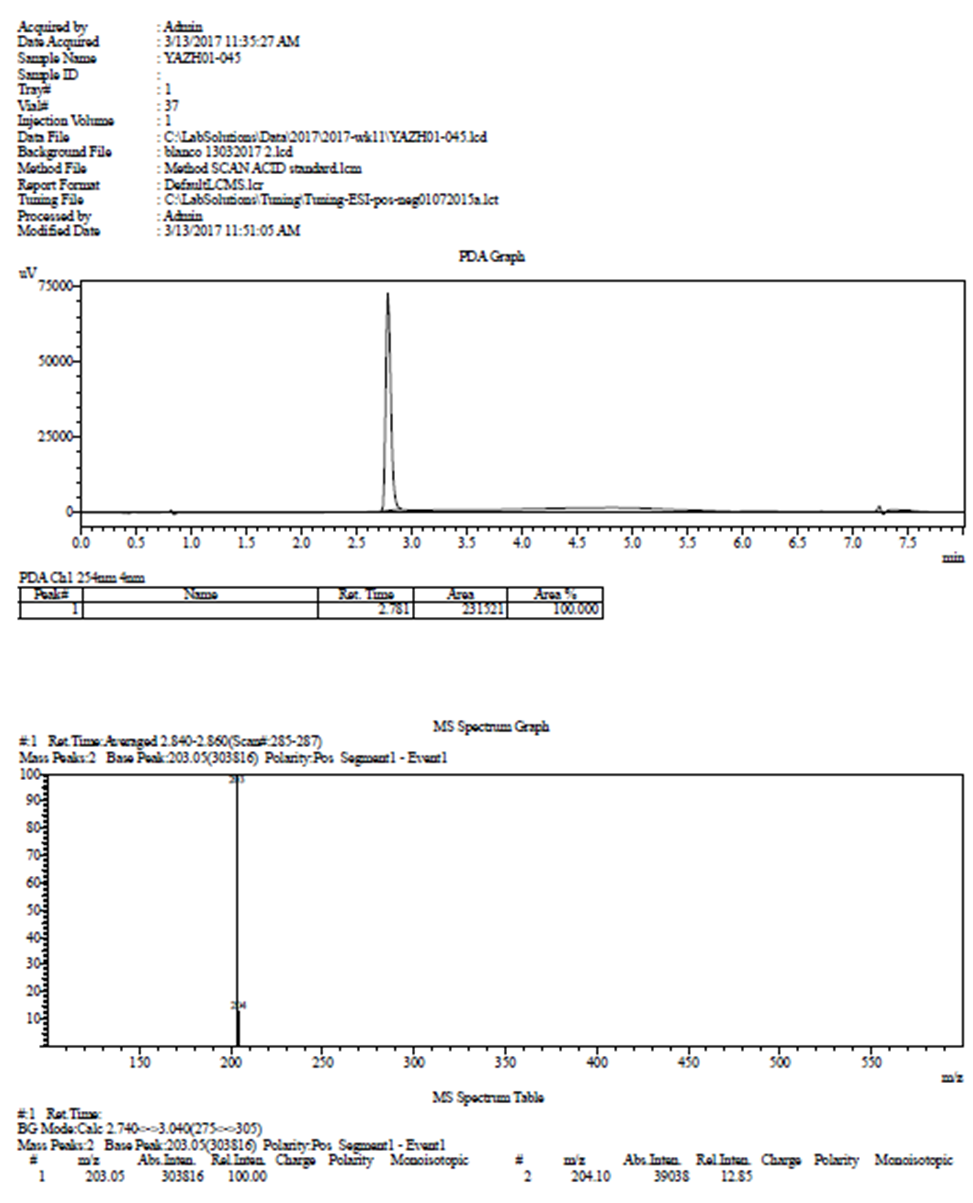


Figure S64. LCMS spectrum of compound **31**

**
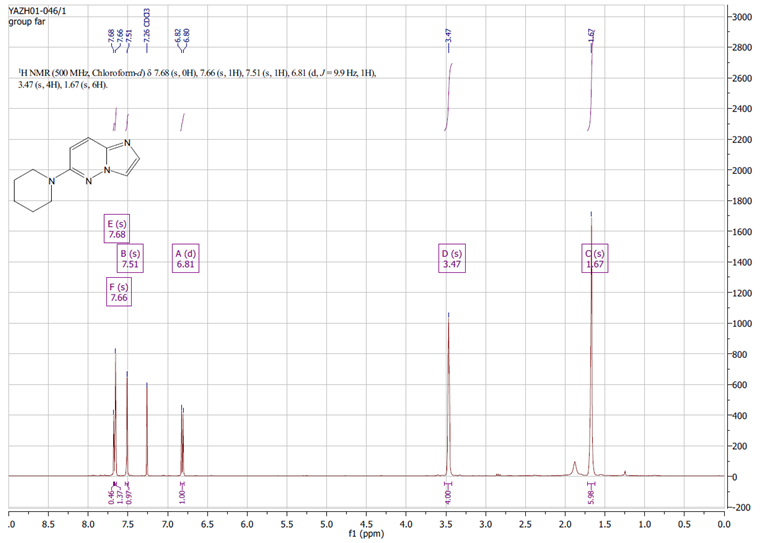
**

Figure S65. ^1^H NMR spectrum of compound **31**

**
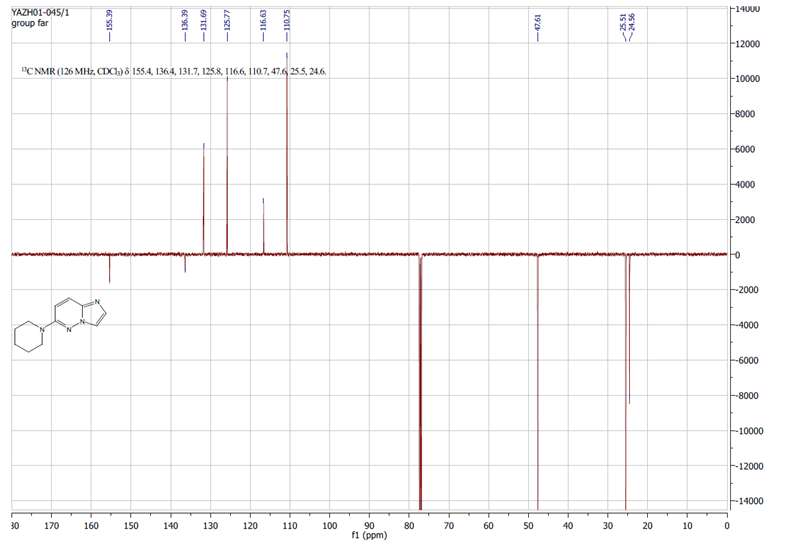
**

Figure S66. ^13^C NMR spectrum of compound **31**


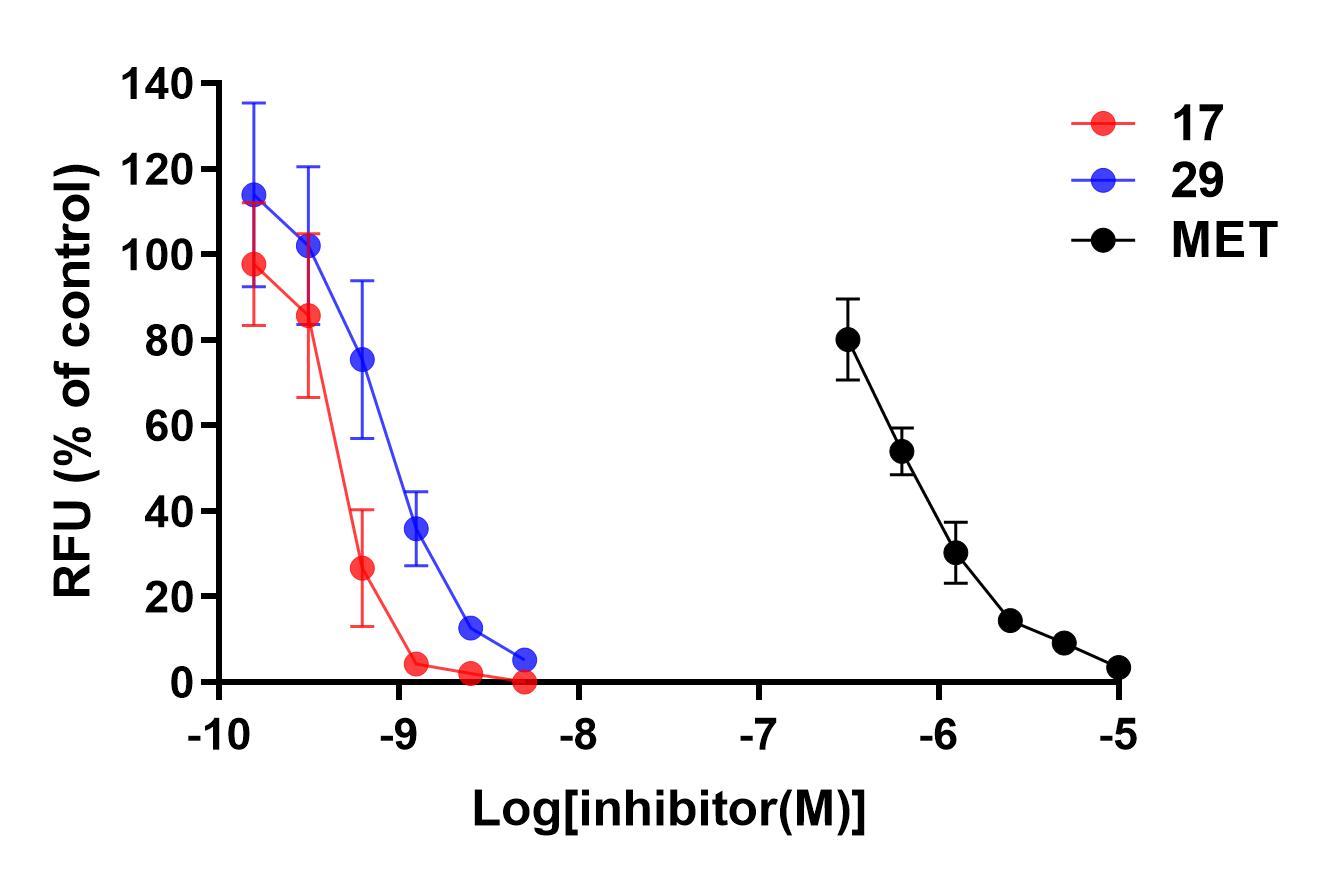


Figure S67. Representative drug susceptibility curves of *G. lamblia* WBC6. Trophozoites were grown anaerobically in the presence of various concentrations of **17** (red), **29** (blue) and the reference compound metronidazole (**MET**, black). After 72 h, growth of cells was monitored by a cell viability assay based on the reduction of resazurin to a pink fluorescent product. Measured fluorescence units were normalized to the DMSO control. Data points and error bars represent mean and standard error of 4 replicates.


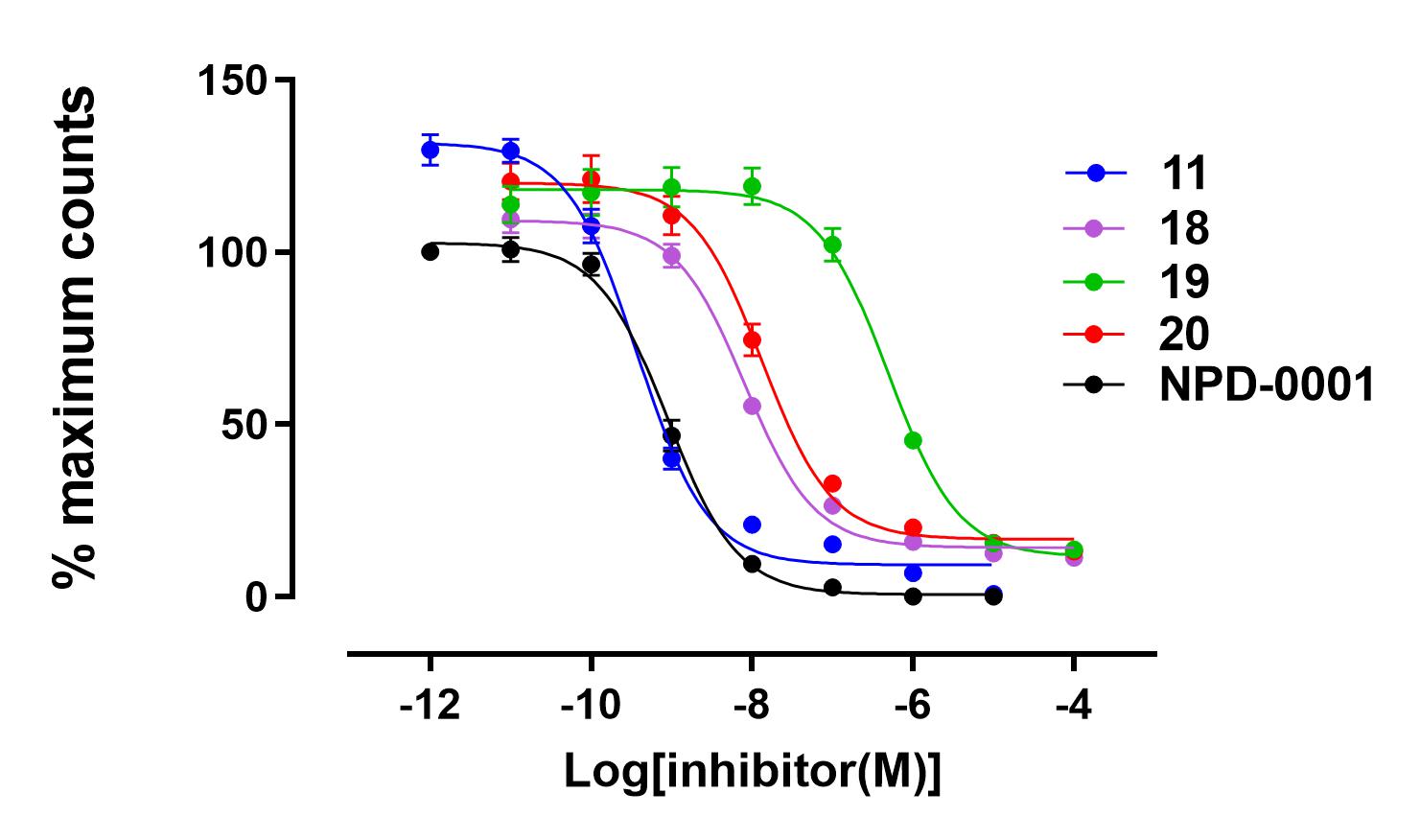


Figure S68. Representative dose-response curves of **11** (blue), **18** (purple), **19** (green) and **20** (red) for inhibition of the enzymatic activity of hPDE4 catalytic domain with **NPD-0001** (black) as a reference compound.


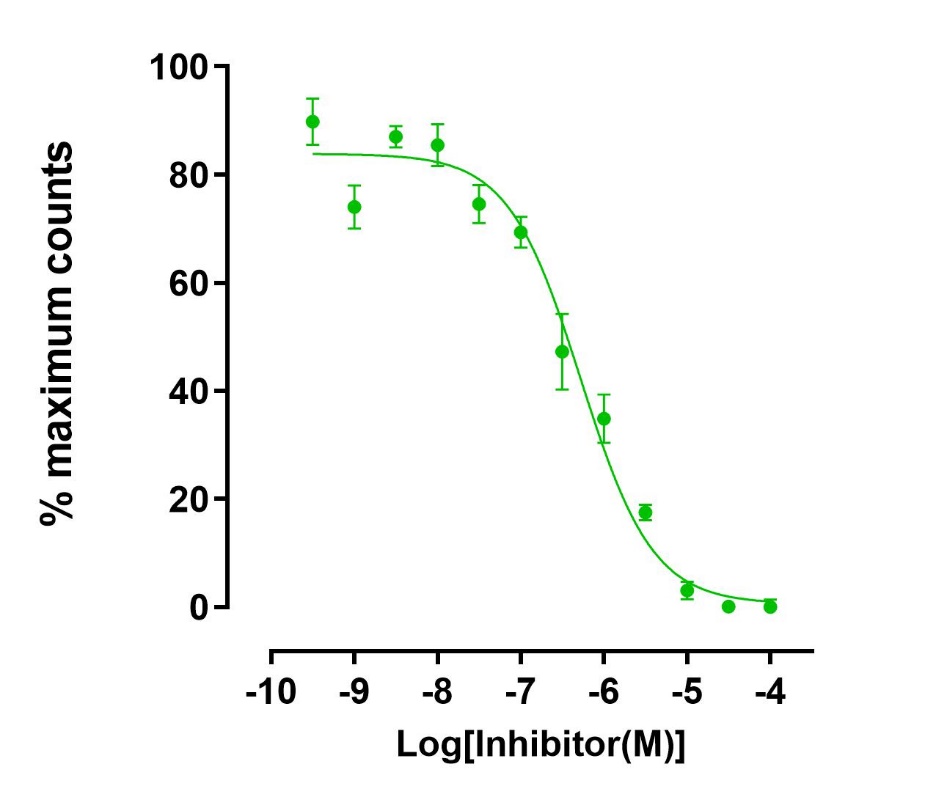


Figure S69. Representative dose-response curve of **11** against GlPDE (aa588-1371) enzyme (substrate concentration: 1 μM).

Table S1: Anti-protozoal activity and toxicity of pyridazines and tetrahydrophthalazinones.

| Cmpd | IC_50_ (µM)^a^ | | | | |
| --- | --- | --- | --- | --- | --- |
|  | *T.b.* | *T.c.* | *L.i.* | *P.f.* | MRC-5 |
| **11** | 1.0 ± 0.7 | 5.9 ± 4.5 | 27.4 ± 25.3 | 6.3 ± 0.4 | >64.0 |
| **12** | 2.1 ± 0.0 | 2.2 ± 0.0 | 25.9 ± 24.4 | 3.0 ± 1.9 | >64.0 |
| **13** | >64.0 | >64.0 | 32.5 ± 0.0 | 1.6 ± 0.7 | >64.0 |
| **14** | >64.0 | 31.8 ± 12.1 | 32.5 ± 0.0 | 0.7 ± 0.5 | >64.0 |
| **15** | 2.2 ± 0.0 | 2.1 ± 0.0 | 8.1 ± 0.0 | 2.1 ± 0.8 | 14.9 ± 11.5 |
| **16** | 1.4 ± 0.4 | 11.2 ± 7.9 | 30.6 ± 2.3 | 1.1 ± 0.5 | 48.9 ± 21.4 |
| **17** | 4.92 ± 1.60 | 2.1 ± 0.1 | 11.8 ± 7.5 | 2.3 ± 1.4 | >64.0 |
| **18** | >64.0 | 50.9 ± 4.7 | 26.4 ± 8.6 | 33.8 ± 37.0 | >64.0 |
| **19** | 22.2 ± 15.9 | 5.4 ± 4.1 | 28.9 ± 31.0 | 7.2 ± 0.4 | >64.0 |
| **20** | >64.0 | >64.0 | >64.0 | >64.0 | >64.0 |
| **21** | >64.0 | >64.0 | >64.0 | >64.0 | >64.0 |
| **22** | >64.0 | >64.0 | >64.0 | >64.0 | >64.0 |
| **23** | >64.0 | 32.3 ± 1.1 | >64.0 | >64.0 | 28.1 ± 1.4 |
| **24** | >64.0 | 33.5 ± 1.1 | >64.0 | >64.0 | >64.0 |
| **25** | >64.0 | 39.9 ± 3.7 | >64.0 | >64.0 | >64.0 |
| **26** | >64.0 | >64.0 | >64.0 | >64.0 | >64.0 |
| **27** | >64.0 | >64.0 | 35.3 ± 4.0 | >64.0 | >64.0 |
| **28** | 27.6 ± 4.2 | 8.6 ± 0.2 | >64.0 | 23.4 ± 7.2 | 8.0 ± 0.2 |
| **29** | 34.4 ± 2.4 | 18.9 ± 13.9 | 11.7 ± 1.4 | >64.0 | >64.0 |
| **30** | >64.0 | 29.7 ± 5.7 | 46.9 ± 5.5 | >64.0 | >64.0 |
| **31** | >64.0 | 38.4 ± 3.2 | >64.0 | >64.0 | >64.0 |
| **1** (**BZN**) | - | 2.5 ± 0.4 | - | - | - |
| **Chloroquine** | - | - | - | 0.1 ± 0.03 | - |
| **Miltefosine** | *-* | - | 10.3 ± 1.3 | - | - |
| **Suramine** | 0.05 ± 0.02 | - | - | - | - |
| **Tamoxifen** | - | - | - | - | 10.7 ± 0.5 |

BZN: benznidazole; *T.b.*: *Trypanosoma brucei brucei*; *T.c.*: *Trypanosoma cruzi*; *L.i.*: *Leishmania infantum*; *P.f.*: *Plasmodium falciparum*; ^a^: the mean ± standard deviation of the mean, n ≥ 2.

1. Note: Supplementary data associated with this article. [↑](#footnote-ref-2)
